# 3D seismic analysis of mine planning using Aczel–Alsina aggregation operators based on T-spherical fuzzy information

**DOI:** 10.1038/s41598-024-54422-0

**Published:** 2024-02-18

**Authors:** Lijun Ma, Kinza Javed, Zeeshan Ali, Tehreem Tehreem, Shi Yin

**Affiliations:** 1 College of Land and Resources, Hebei Agricultural University, Baoding, 071000 China; 2 Department of Mathematics and Statistics, Riphah International University, Islamabad, 44000 Pakistan; 3 Department of Mathematics, Faculty of Basic and Applied Sciences, Air University PAF Complex, E-9, Islamabad, 44000 Pakistan; 4 College of Economics and Management, Hebei Agriculture University, Baoding, 071001 China

**Keywords:** Complex T-spherical hesitant fuzzy sets, Aczel–Alsina power aggregation operators, Analysis of goldmines, Decision-making problems, Engineering, Civil engineering, Applied mathematics

## Abstract

3D seismic attributes analysis can help geologists and mine developers associate subsurface geological features, structures, faults, and ore bodies more precisely and accurately. The major influence of this application is to evaluate the usage of the 3D seismic attributes analysis in gold mine planning. For this, we evaluate the novel theory of complex T-spherical hesitant fuzzy (CTSHF) sets and their operational laws. Furthermore, we derive the CTSHF Aczel–Alsina weighted power averaging (CTSHFAAWPA) operator, CTSHF Aczel–Alsina ordered weighted power averaging (CTSHFAAOWPA) operator, CTSHF Aczel–Alsina weighted power geometric (CTSHFAAWPG) operator, and CTSHF Aczel–Alsina ordered.com weighted power geometric (CTSHFAAOWPG) operator. Some properties are also investigated for the above operators. Additionally, we evaluate the problems of 3D seismic attributes analysis to mine planning under the consideration of the proposed operators, for this, we illustrate the problem of the multi-attribute decision-making (MADM) technique for the above operators. Finally, we demonstrate some examples for making the comparison between prevailing and proposed information to improve the worth of the derived operators.

## Introduction

The application of 3D seismic attributes analysis to mine planning can be valuable and dominant in the mining industry and many organizations and it plays an essential role in the environment of the economy of the considered country. Many companies and industries are working on it under the consideration of some classical or crip set theory. Where crisp set theory has failed to persuade the unknown data in decision-making. To overcome this slot in 1965, Zadeh^1^ represented the idea of the fuzzy set (FS) with a positive grade under the range of [0,1]. Zadeh showed that FS is a dominant technique for depicting unpredictable data or problems^2^. Furthermore, Atanassov^3,4^ introduced the technique of intuitionistic FS (IFS), where the technique of IFS is the modified version of the old FS, because the FS has only one grade such as a positive grade, but the IFS has two grades such as positive and negative with a prominent characteristic, such as the sum of the pair will be contained in the unit interval. Furthermore, many kinds of extensions of IFS have been proposed by different scholars, for instance, Pythagorean FS (PyFS)^5^, and q-rung orthopair FS (QROFS)^6^. But these kinds of theories are not enough for depicting unreliable and vague kind of data, because in many cases, we noticed that these all existing theories are not working effectively, therefore, the novel theory of picture FS (PFS) was initiated by Cuong^7^, where the PFS has three different functions, such as positive, abstinence, and negative with a condition that is the sum of triplet will be containing in unit interval. We observed that the theory of PFs is the modified version of the IFS but not the PyFS and QROFS. For managing such kind of problems, the novel theory of spherical FS (SFS) and T-spherical FS (TSFS) was initiated by Mahmood et al.^8^. The theory of Mahmood et al. is very reliable and superior because many existing ideas are the special cases of the TSFSs, for instance, aggregation operators^9,10^, similarity measures^11,12^, and decision-making^13,14^ problems.

With time, many problems have occurred in fuzzy set theory, where many questions circulate among mathematicians, such as when we change the codomain [0,1] with a complex number what happens? To solve this problem Ramot et al.^15^ gave the theory of complex FS (CFS) in 2002, which initiates the positive grade in the shape of a complex number instead of a real number from interval [0,1]. Moreover, Salleh and Alkouri^16^ expanded the concept of complex IFS (CIFS) by including the negative grade in CFS. CIFS is very reliable but in many situations, it is not working dominantly because of its limitations, therefore, many scholars have proposed different kinds of techniques such as complex PyFS (CPyFS)^17^ and complex QROFS (CQROFS)^18^. Furthermore, utilizing the grade of abstinence in the shape of the complex number is very complex and complicated, therefore, Akram et al.^19^ initiated the theory of complex PFS (CPFS), where the CPFS contained the grade of positive, abstinence, negative with a condition that is the sum of the real part of the triplet will be containing in unit interval. Furthermore, Ali et al.^20^ initiated the theory of complex SFS (CSFS), where the CSFS is the modified version of the PyFS and their related ideas, but still, the problem of QROFS has needed to be evaluated, for this, Ali et al.^21^ derived the theory of complex TSFS (CTSFS), which is more superior, and dominant is compared to existing techniques.

Furthermore, the hesitant FS (HFS) was initiated by Torra^22^ in 2010, which is the most preferable and most dominant theory because it is the modified version of the FS, where the positive grade in HFS is computed in the shape of the collection of membership grades, where the complex HFS (CHFS) was initiated by Mahmood et al.^23^ Furthermore, for evaluating any kind of operators we have required some strong results for computing some aggregation operators, for this, we revised the theory of Aczel and Alsina^24^, called Aczel–Alsina t-norm and t-conorm, proposed in 1982. Furthermore, Yager^25^ initiated the novel theory of power-averaging operators based on classical set theory which is beneficial for aggregating the collection of data. Additionally, many peoples have derived different kind of operators, such as Aczel–Alsina operators (AAOs) for HFSs^26^, AAOs for IFSs^27^, AAOs for PFSs^28,29^, AAOs for CPFSs^30^, AAOs for IFSs^31^, geometric AAOs for IFSs^32^, AAOs for PyFSs^33^, AAOs for QROFSs^34^, AAOs for TSFSs^35^, and AAOs for CTSFSs^36^. Further, different kinds of operators, methods, and measures have been proposed for their special cases, for instance, MARCOS techniques^37^, decision-making techniques for PFSs^38^, LOPCOW-ARAS techniques^39^, and TOPSIS techniques^40^. After a brief analysis, we observed that during the decision-making process, every decision-maker faced the following problems, such as:

1. How do we evaluate a superior and novel concept?
2. How do we calculate the new operational laws?
3. How do we derive any kind of operators?
4. How do we rank all alternatives?

These problems are the essential parts of every decision-making procedure and for this, we concentrate on initiating the Aczel–Alsina power operators based on a novel idea of CTSHF sets. The major problem with existing techniques is that when an expert provides the truth, abstinence, falsity, and refusal information are in the shape of hesitant fuzzy numbers. Furthermore, we observed that the power operators based on Aczel–Alsina t-norm and t-conorms based on the novel theory of the CTSHF set have not been proposed yet, because of complicated problems. It is very awkward and complicated to compute the idea of the CTSHF set and it is also very ambiguous to compute the Aczel–Alsina power operators for it. Anyhow, some special cases of the proposed theory are listed below:

1. Averaging operator for FSs and their extensions.
2. Geometric operator for FSs and their extensions.
3. Aczel–Alsina averaging operator for FSs and their extensions.
4. Aczel–Alsina geometric operator for FSs and their extensions.
5. Power averaging operator for FSs and their extensions.
6. Power geometric operator for FSs and their extensions.
7. Aczel–Alsina power averaging operator for FSs and their extensions.
8. Aczel–Alsina power geometric operator for FSs and their extensions.

The above concepts are the advantages of the initiated techniques, and due to this reason, it is much different from others and also superior them many existing ideas. Inspired by the above information, the major contribution of this manuscript is listed below:

1. To evaluate the novel theory of the CTSHF set and its operational laws.
2. To investigate Aczel–Alsina operational laws for CTSHF information.
3. To derive the CTSHFAAWPA operator, CTSHFAAOWPA operator, CTSHFAAWPG operator, and CTSHFAAOWPG operator. Some properties are also investigated for the above operators.
4. To evaluate the problems of 3D seismic attributes analysis to mine planning under the consideration of the proposed operators, we illustrate the problem of the MADM technique for the above operators.
5. To demonstrate some examples for making the comparison between prevailing and proposed information to improve the worth of the derived operators.

**Figure 1:**
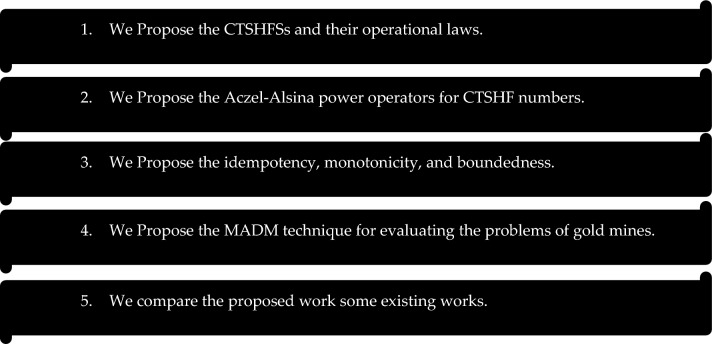
Graphical abstract of the proposed information.

This manuscript is arranged in the shape: In Section “Preliminaries”, we discussed the HFSs, CTSFSs, Aczel–Alsina norms, and their related properties. In “CTSHFSs” section, we evaluated the novel theory of the CTSHF set and their operational laws. In “Aczel–Alsina power aggregation operators for CTSHFSs” section, we derived the CTSHFAAWPA operator, CTSHFAAOWPA operator, CTSHFAAWPG operator, and CTSHFAAOWPG operator. Some properties are also investigated for the above operators. In “Decision-making procedure based on proposed operators” section, we evaluated the problems of 3D seismic attributes analysis to mine planning under the consideration of the proposed operators, for this, we illustrated the problem of the MADM technique for the above operators. In “Comparative analysis” section, we demonstrated some examples for making the comparison between prevailing and proposed information to improve the worth of the derived operators. Some concluding remarks are discussed in “Conclusion”. The geometrical representation of this manuscript is listed in the shape of Fig. 1.

## Preliminaries

In this section, we discussed the HFSs, CTSFSs, and Aczel–Alsina norms and their related properties.

### Definition 1

^22^ A HFS ${\widehat{\mathbb{H}}}_{h}$ is initiated by:

$${\widehat{\mathbb{H}}}_{h}=\left\{\left(x,{\overline{\overline{\mathbb{p}}}}_{{\widehat{\mathbb{H}}}_{h}}\left(x\right)\right):x\in {\psi }^{uni}\right\}$$

Observed that ${\overline{\overline{\mathbb{p}}}}_{{\widehat{\mathbb{H}}}_{h}}\left(x\right)=\left\{{\overline{\overline{\mathbb{T}}}}_{{\widehat{\mathbb{H}}}_{h}}\left(x\right):j=\mathrm{1,2},3\dots ,n\right\}$ represents the positive grade where ${\overline{\overline{\mathbb{T}}}}_{{\widehat{\mathbb{H}}}_{h}}\left(x\right)\in [{\rm O},1]$.

### Definition 2

^21^ A CTSFS ${\widehat{\mathbb{H}}}_{p}$ is computed in the form:

$${\widehat{\mathbb{H}}}_{p}=\left\{(x,{(\overline{\overline{\mathbb{T}}}}_{{\widehat{\mathbb{H}}}_{p}}\left(x\right),{\overline{\overline{\mathbb{V}}}}_{{\widehat{\mathbb{H}}}_{p}}\left(x\right),{\overline{\overline{\mathbb{U}}}}_{{\widehat{\mathbb{H}}}_{p}}\left(x\right))):x\in {\psi }^{uni}\right\}$$

We can observe that the positive grade is computed in the shape: ${\overline{\overline{\mathbb{T}}}}_{{\widehat{\mathbb{H}}}_{p}}\left(x\right)={\overline{\overline{\mathbb{T}}}}_{{\widehat{\mathbb{H}}}_{r}}\left(x\right){\mathcal{e}}^{i2\Pi ( {\overline{\overline{\mathbb{T}}}}_{{\widehat{\mathbb{H}}}_{i}\left(x\right)})}$, where the negative grade is invented by: ${\overline{\overline{\mathbb{U}}}}_{{\widehat{\mathbb{H}}}_{p}}\left(x\right)={\overline{\overline{\mathbb{U}}}}_{{\widehat{\mathbb{H}}}_{r}}\left(x\right){\mathcal{e}}^{i2\Pi ( {\overline{\overline{\mathbb{U}}}}_{{\widehat{\mathbb{H}}}_{i}\left(x\right)})}$ with ${{(\overline{\overline{\mathbb{T}}}}_{{\widehat{\mathbb{H}}}_{r}}\left(x\right) )}^{\pi }+{ {(\overline{\overline{\mathbb{V}}}}_{{\widehat{\mathbb{H}}}_{r}}\left(x\right) ) }^{\pi }+{ {(\overline{\overline{\mathbb{U}}}}_{{\widehat{\mathbb{H}}}_{r}}\left(x\right) ) }^{\pi }, { {(\overline{\overline{\mathbb{T}}}}_{{\widehat{\mathbb{H}}}_{i}}\left(x\right) ) }^{\pi }{ {(\overline{\overline{\mathbb{V}}}}_{{\widehat{\mathbb{H}}}_{a}}\left(x\right) ) }^{\pi }{ {(\overline{\overline{\mathbb{U}}}}_{{\widehat{\mathbb{H}}}_{i}}\left(x\right) ) }^{\pi } \epsilon \left[{\rm O},1\right].$ Moreover, the neutral grade is the following: $${\overline{\overline{\mathbb{r}}}}_{{\widehat{\mathbb{H}}}_{p}}\left(x\right)={\overline{\overline{\mathbb{r}}}}_{{\widehat{\mathbb{H}}}_{r}}\left(x\right){\mathcal{e}}^{i2\Pi \left( {\overline{\overline{\mathbb{r}}}}_{{\widehat{\mathbb{H}}}_{i}\left(x\right)}\right)}={\left(\begin{array}{c}{1- {((\overline{\overline{\mathbb{T}}}}_{{\widehat{\mathbb{H}}}_{r}}\left(x\right) ) }^{\pi }+\\ { {(\overline{\overline{\mathbb{V}}}}_{{\widehat{\mathbb{H}}}_{r}}\left(x\right) ) }^{\pi }+{ {(\overline{\overline{\mathbb{U}}}}_{{\widehat{\mathbb{H}}}_{r}}\left(x\right) ) }^{\pi }\end{array}\right)}^{\frac{1}{\pi }} {\mathcal{e}}^{{i2\Pi \left(1-\left({ {(\overline{\overline{\mathbb{T}}}}_{{\widehat{\mathbb{H}}}_{i}}\left(x\right) ) }^{\pi }{ {(\overline{\overline{\mathbb{V}}}}_{{\widehat{\mathbb{H}}}_{a}}\left(x\right) ) }^{\pi }{ {(\overline{\overline{\mathbb{U}}}}_{{\widehat{\mathbb{H}}}_{i}}\left(x\right) ) }^{\pi }\right)\right)}^{\frac{1}{\pi }}}$$. The mathematical shape of the CTSF number (CTSFN) ${\widehat{\mathbb{H}}}_{p}^{j}=\left({\overline{\overline{\mathbb{T}}}}_{{\widehat{\mathbb{H}}}_{r}}^{j}{\mathcal{e}}^{i2\Pi \left({\overline{\overline{\mathbb{T}}}}_{{\widehat{\mathbb{H}}}_{i}}^{j}\right)},{\overline{\overline{\mathbb{V}}}}_{{\widehat{\mathbb{H}}}_{r}}^{j}{\mathcal{e}}^{i2\Pi \left({\overline{\overline{\mathbb{V}}}}_{{\widehat{\mathbb{H}}}_{a}}^{j}\right)},{\overline{\overline{\mathbb{U}}}}_{{\widehat{\mathbb{H}}}_{r}}^{j}{\mathcal{e}}^{i2\Pi \left({\overline{\overline{\mathbb{U}}}}_{{\widehat{\mathbb{H}}}_{i}}^{j}\right)}\right),j=\mathrm{1,2},\dots ,n$, will be using the overall manuscript.

### Definition 3

^21^ Some algebraic laws for any two CTSFNs are stated below:

$${\widehat{\mathbb{H}}}_{p}^{1} \oplus{\widehat{\mathbb{H}}}_{p}^{2}=\left(\begin{array}{c}{\left({\left({\overline{\overline{\mathbb{T}}}}_{{\widehat{\mathbb{H}}}_{r}}^{1}\right)}^{\pi }+{\left({\overline{\overline{\mathbb{T}}}}_{{\widehat{\mathbb{H}}}_{r}}^{2}\right)}^{\pi }-{\left({\overline{\overline{\mathbb{T}}}}_{{\widehat{\mathbb{H}}}_{r}}^{1}\right)}^{\pi }{\left({\overline{\overline{\mathbb{T}}}}_{{\widehat{\mathbb{H}}}_{r}}^{2}\right)}^{\pi }\right)}^{\frac{1}{\pi }} {\mathcal{e}}^{{i2\Pi \left({\left({\overline{\overline{\mathbb{T}}}}_{{\widehat{\mathbb{H}}}_{i}}^{1}\right)}^{\pi }+{ \left({\overline{\overline{\mathbb{T}}}}_{{\widehat{\mathbb{H}}}_{i}}^{2} \right)}^{\pi }- {\left({\overline{\overline{\mathbb{T}}}}_{{\widehat{\mathbb{H}}}_{i}}^{1}\right)}^{\pi }{\left({\overline{\overline{\mathbb{T}}}}_{{\widehat{\mathbb{H}}}_{i}}^{1}\right)}^{\pi } \right)}^{\frac{1}{\pi }}},\\ \left( {\overline{\overline{\mathbb{V}}}}_{{\widehat{\mathbb{H}}}_{r}}^{1}{\overline{\overline{\mathbb{V}}}}_{{\widehat{\mathbb{H}}}_{r},}^{2}\right){\mathcal{e}}^{i2\Pi \left({\overline{\overline{\mathbb{V}}}}_{{\widehat{\mathbb{H}}}_{a}}^{1}{\overline{\overline{\mathbb{V}}}}_{{\widehat{\mathbb{H}}}_{a},}^{2}\right)},\left( {\overline{\overline{f}}}_{{\widehat{\mathbb{H}}}_{r}}^{1}{\overline{\overline{f}}}_{{\widehat{\mathbb{H}}}_{r},}^{2}\right){\mathcal{e}}^{i2\Pi \left({\overline{\overline{f}}}_{{\widehat{\mathbb{H}}}_{i} }^{1}{\overline{\overline{f}}}_{{\widehat{\mathbb{H}}}_{i} }^{2}\right)}\end{array}\right)$$

$${\widehat{\mathbb{H}}}_{p}^{1}\otimes {\widehat{\mathbb{H}}}_{p}^{2}=\left(\begin{array}{c}\left({\overline{\overline{\mathbb{T}}}}_{{\widehat{\mathbb{H}}}_{r}}^{1}{\overline{\overline{\mathbb{T}}}}_{{\widehat{\mathbb{H}}}_{r},}^{2}\right){\mathcal{e}}^{i2\Pi \left({\overline{\overline{\mathbb{T}}}}_{{\widehat{\mathbb{H}}}_{i}}^{1}{\overline{\overline{\mathbb{T}}}}_{{\widehat{\mathbb{H}}}_{i},}^{2} \right)}, \begin{array}{c}{\left({\left({\overline{\overline{\mathbb{V}}}}_{{\widehat{\mathbb{H}}}_{r}}^{1}\right)}^{\pi }+{\left({\overline{\overline{\mathbb{V}}}}_{{\widehat{\mathbb{H}}}_{r}}^{2}\right)}^{\pi }-{\left({\overline{\overline{\mathbb{V}}}}_{{\widehat{\mathbb{H}}}_{r}}^{1}\right)}^{\pi }{\left({\overline{\overline{\mathbb{V}}}}_{{\widehat{\mathbb{H}}}_{r}}^{1}\right)}^{\pi }\right)}^{\frac{1}{\pi }} {\mathcal{e}}^{{i2\Pi \left({\left({\overline{\overline{\mathbb{V}}}}_{{\widehat{\mathbb{H}}}_{a}}^{1}\right)}^{\pi }\left.+{ \left({\overline{\overline{\mathbb{V}}}}_{{\widehat{\mathbb{H}}}_{a}}^{2} \right)}^{\pi }- {\left({\overline{\overline{\mathbb{V}}}}_{{\widehat{\mathbb{H}}}_{a}}^{1}\right)}^{\pi }{\left({\overline{\overline{\mathbb{V}}}}_{{\widehat{\mathbb{H}}}_{a}}^{1}\right)}^{\pi }\right) \right)}^{\frac{1}{\pi }}},\\ {\left({\left({\overline{\overline{\mathbb{U}}}}_{{\widehat{\mathbb{H}}}_{r}}^{1}\right)}^{\pi }+{\left({\overline{\overline{\mathbb{U}}}}_{{\widehat{\mathbb{H}}}_{r}}^{2}\right)}^{\pi }-{\left({\overline{\overline{\mathbb{U}}}}_{{\widehat{\mathbb{H}}}_{r}}^{1}\right)}^{\pi }{\left({\overline{\overline{\mathbb{U}}}}_{{\widehat{\mathbb{H}}}_{r}}^{1}\right)}^{\pi }\right)}^{\frac{1}{\pi }} {\mathcal{e}}^{{i2\Pi \left({\left({\overline{\overline{\mathbb{U}}}}_{{\widehat{\mathbb{H}}}_{i}}^{1}\right)}^{\pi }+{ \left({\overline{\overline{\mathbb{U}}}}_{{\widehat{\mathbb{H}}}_{i}}^{2} \right)}^{\pi }- {\left({\overline{\overline{\mathbb{U}}}}_{{\widehat{\mathbb{H}}}_{i}}^{1}\right)}^{\pi }{\left({\overline{\overline{\mathbb{U}}}}_{{\widehat{\mathbb{H}}}_{i}}^{2}\right)}^{\pi } \right)}^{\frac{1}{\pi }}} \end{array}\end{array}\right)$$

$${\widehat{\Xi }}_{s}{\widehat{\mathbb{H}}}_{p}^{1}=\left({\left(1-{\left(1-{\left({\overline{\overline{\mathbb{T}}}}_{{\widehat{\mathbb{H}}}_{r}}^{1}\right)}^{\pi }\right)}^{{\widehat{\Xi }}_{s}}\right)}^{\frac{1}{\pi }}{\mathcal{e}}^{i2\Pi {\left(1-{\left(1-{\left({\mathbb{T}}_{{\widehat{\mathbb{H}}}_{i}}^{1}\right)}^{\pi }\right)}^{{\widehat{\Xi }}_{s}}\right)}^{\frac{1}{\pi }}},{\left({\overline{\overline{\mathbb{V}}}}_{{\widehat{\mathbb{H}}}_{r}}^{1}\right)}^{{\widehat{\Xi }}_{s}}{\mathcal{e}}^{i2\Pi {\left({\overline{\overline{\mathbb{V}}}}_{{\widehat{\mathbb{H}}}_{a}}^{1}\right)}^{{\widehat{\Xi }}_{s}}},{\left({\overline{\overline{\mathbb{U}}}}_{{\widehat{\mathbb{H}}}_{r}}^{1}\right)}^{{\widehat{\Xi }}_{s}}{\mathcal{e}}^{i2\Pi {\left({\overline{\overline{\mathbb{U}}}}_{{\widehat{\mathbb{H}}}_{i}}^{1}\right)}^{{\widehat{\Xi }}_{s}}}\right)$$

$${{(\widehat{\mathbb{H}}}_{p}^{1})}^{{\widehat{\Xi }}_{s}}=\left(\genfrac{}{}{0pt}{}{{ \left({\overline{\overline{\mathbb{U}}}}_{{\widehat{\mathbb{H}}}_{r}}^{1}\right)}^{{\widehat{\Xi }}_{s}}{\mathcal{e}}^{i2\Pi {\left({\overline{\overline{\mathbb{T}}}}_{{\widehat{\mathbb{H}}}_{a}}^{1}\right)}^{{\widehat{\Xi }}_{s}}},{\left(1-{\left(1-{\left({\overline{\overline{\mathbb{V}}}}_{{\widehat{\mathbb{H}}}_{r}}^{1}\right)}^{\pi }\right)}^{{\widehat{\Xi }}_{s}}\right)}^{\frac{1}{\pi }}{\mathcal{e}}^{i2\Pi {\left(1-{\left(1-{\left({\overline{\overline{\mathbb{V}}}}_{{\widehat{\mathbb{H}}}_{a}}^{1}\right)}^{\pi }\right)}^{{\widehat{\Xi }}_{s}}\right)}^{\frac{1}{\pi }}},}{ {\left(1-{\left(1-{\left({\overline{\overline{\mathbb{U}}}}_{{\widehat{\mathbb{H}}}_{r}}^{1}\right)}^{\pi }\right)}^{{\widehat{\Xi }}_{s}}\right)}^{\frac{1}{\pi }}{\mathcal{e}}^{i2\Pi {\left(1-{\left(1-{\left({\overline{\overline{\mathbb{U}}}}_{{\widehat{\mathbb{H}}}_{i}}^{1}\right)}^{\pi }\right)}^{{\widehat{\Xi }}_{s}}\right)}^{\frac{1}{\pi }}}}\right)$$

### Definition 4

^36^ Some Aczel–Alsina operational laws for any two CTSFNs are evaluated below:

$${\widehat{\mathbb{H}}}_{p}^{1}\oplus {\widehat{\mathbb{H}}}_{p}^{2}=\left(\begin{array}{c}\genfrac{}{}{0pt}{}{{\left(1-{\mathcal{e}}^{-{\left({\left(-{\text{ln}}\left(1-{\left({\overline{\overline{\mathbb{T}}}}_{{\widehat{\mathbb{H}}}_{r}}^{1}\right)}^{\pi }\right)\right)}^{\varnothing }+{\left(-{\text{ln}}\left(1-{\left({\overline{\overline{\mathbb{T}}}}_{{\widehat{\mathbb{H}}}_{r}}^{2}\right)}^{\pi }\right)\right)}^{\varnothing }\right)}^{\frac{1}{\varnothing }}}\right)}^{\frac{1}{\pi }}{\mathcal{e}}^{i2\Pi {\left(1-{\mathcal{e}}^{-{\left({\left(-{\text{ln}}\left(1-{\left({\overline{\overline{\mathbb{T}}}}_{{\widehat{\mathbb{H}}}_{i}}^{1}\right)}^{\pi }\right)\right)}^{\varnothing }+{\left(-{\text{ln}}\left(1-{\left({\overline{\overline{\mathbb{T}}}}_{{\widehat{\mathbb{H}}}_{i}}^{2}\right)}^{\pi }\right)\right)}^{\varnothing }\right)}^{\frac{1}{\varnothing }}}\right)}^\frac{1}{2}},}{{\left({\mathcal{e}}^{-{\left({\left(-{\text{ln}}\left({\left({\overline{\overline{\mathbb{V}}}}_{{\widehat{\mathbb{H}}}_{r}}^{1}\right)}^{\pi }\right)\right)}^{\varnothing }+{\left(-{\text{ln}}\left({\left({\overline{\overline{\mathbb{V}}}}_{{\widehat{\mathbb{H}}}_{r}}^{2}\right)}^{\pi }\right)\right)}^{\varnothing }\right)}^{\frac{1}{\varnothing }}}\right)}^{\frac{1}{\pi }} {\mathcal{e}}^{i2\Pi {\left({\mathcal{e}}^{-{\left({\left(-{\text{ln}}\left({\left({\overline{\overline{\mathbb{V}}}}_{{\widehat{\mathbb{H}}}_{a}}^{1}\right)}^{\pi }\right)\right)}^{\varnothing }+{\left(-{\text{ln}}\left({\left({\overline{\overline{\mathbb{V}}}}_{{\widehat{\mathbb{H}}}_{a}}^{2}\right)}^{\pi }\right)\right)}^{\varnothing }\right)}^{\frac{1}{\varnothing }}}\right)}^{\frac{1}{\pi }}},}\\ {\left({\mathcal{e}}^{-{\left({\left(-{\text{ln}}\left({\left({\overline{\overline{\mathbb{U}}}}_{{\widehat{\mathbb{H}}}_{r}}^{1}\right)}^{\pi }\right)\right)}^{\varnothing }+{\left(-{\text{ln}}\left({\left({\overline{\overline{\mathbb{U}}}}_{{\widehat{\mathbb{H}}}_{r}}^{2}\right)}^{\pi }\right)\right)}^{\varnothing }\right)}^{\frac{1}{\varnothing }}}\right)}^{\frac{1}{\pi }} {\mathcal{e}}^{i2\Pi {\left({\mathcal{e}}^{-{\left({\left(-{\text{ln}}\left({\left({\overline{\overline{\mathbb{U}}}}_{{\widehat{\mathbb{H}}}_{i}}^{1}\right)}^{\pi }\right)\right)}^{\varnothing }+{\left(-{\text{ln}}\left({\left({\overline{\overline{\mathbb{U}}}}_{{\widehat{\mathbb{H}}}_{i}}^{2}\right)}^{\pi }\right)\right)}^{\varnothing }\right)}^{\frac{1}{\varnothing }}}\right)}^{\frac{1}{\pi }}}\end{array}\right)$$

$${\widehat{\mathbb{H}}}_{p}^{1}\otimes {\widehat{\mathbb{H}}}_{p}^{2}=\left(\begin{array}{c}\genfrac{}{}{0pt}{}{{\left({\mathcal{e}}^{-{\left({\left(-{\text{ln}}\left({\left({\overline{\overline{\mathbb{T}}}}_{{\widehat{\mathbb{H}}}_{r}}^{1}\right)}^{\pi }\right)\right)}^{\varnothing }+{\left(-{\text{ln}}\left({\left({\overline{\overline{\mathbb{T}}}}_{{\widehat{\mathbb{H}}}_{r}}^{2}\right)}^{\pi }\right)\right)}^{\varnothing }\right)}^{\frac{1}{\varnothing }}}\right)}^{\frac{1}{\pi }}{\mathcal{e}}^{i2\Pi {\left({\mathcal{e}}^{-{\left({\left(-{\text{ln}}\left({\left({\overline{\overline{\mathbb{T}}}}_{{\widehat{\mathbb{H}}}_{i}}^{1}\right)}^{\pi }\right)\right)}^{\varnothing }+{\left(-{\text{ln}}\left({\left({\overline{\overline{\mathbb{T}}}}_{{\widehat{\mathbb{H}}}_{i}}^{2}\right)}^{\pi }\right)\right)}^{\varnothing }\right)}^{\frac{1}{\varnothing }}}\right)}^{\frac{1}{\pi }}},}{{\left(1-{\mathcal{e}}^{-{\left({\left(-{\text{ln}}\left(1-{\left({\overline{\overline{\mathbb{V}}}}_{{\widehat{\mathbb{H}}}_{r}}^{1}\right)}^{\pi }\right)\right)}^{\varnothing }+{\left(-{\text{ln}}\left(1-{\left({\overline{\overline{\mathbb{V}}}}_{{\widehat{\mathbb{H}}}_{r}}^{2}\right)}^{\pi }\right)\right)}^{\varnothing }\right)}^{\frac{1}{\varnothing }}}\right)}^{\frac{1}{\pi }} {\mathcal{e}}^{i2\Pi {\left(1-{\mathcal{e}}^{-{\left({\left(-{\text{ln}}\left(1-{\left({\overline{\overline{\mathbb{V}}}}_{{\widehat{\mathbb{H}}}_{i}}^{1}\right)}^{\pi }\right)\right)}^{\varnothing }+{\left(-{\text{ln}}\left({1-\left({\overline{\overline{\mathbb{V}}}}_{{\widehat{\mathbb{H}}}_{a}}^{2}\right)}^{\pi }\right)\right)}^{\varnothing }\right)}^{\frac{1}{\varnothing }}}\right)}^{\frac{1}{\pi }}},}\\ {\left({1-\mathcal{e}}^{-{\left({\left(-{\text{ln}}\left(1-{\left({\overline{\overline{\mathbb{U}}}}_{{\widehat{\mathbb{H}}}_{r}}^{1}\right)}^{\pi }\right)\right)}^{\varnothing }+{\left(-{\text{ln}}\left({1-\left({\overline{\overline{\mathbb{U}}}}_{{\widehat{\mathbb{H}}}_{r}}^{2}\right)}^{\pi }\right)\right)}^{\varnothing }\right)}^{\frac{1}{\varnothing }}}\right)}^{\frac{1}{\pi }} {\mathcal{e}}^{i2\Pi {\left(1-{\mathcal{e}}^{-{\left({\left(-{\text{ln}}\left(1-{\left({\overline{\overline{\mathbb{U}}}}_{{\widehat{\mathbb{H}}}_{i}}^{1}\right)}^{\pi }\right)\right)}^{\varnothing }+{\left(-{\text{ln}}\left(1-{\left({\overline{\overline{\mathbb{U}}}}_{{\widehat{\mathbb{H}}}_{i}}^{2}\right)}^{\pi }\right)\right)}^{\varnothing }\right)}^{\frac{1}{\varnothing }}}\right)}^{\frac{1}{\pi }}}\end{array}\right)$$

$${\widehat{\Xi }}_{s}{\widehat{\mathbb{H}}}_{p}^{1}=\left(\begin{array}{c}\genfrac{}{}{0pt}{}{\begin{array}{c}{\left(1-{\mathcal{e}}^{-{\left({\widehat{\Xi }}_{s}{\left(-{\text{ln}}\left(1-{\left({\overline{\overline{\mathbb{T}}}}_{{\widehat{\mathbb{H}}}_{r}}^{1}\right)}^{\pi }\right)\right)}^{\varnothing }\right)}^{\frac{1}{\varnothing }}}\right)}^{\frac{1}{\pi }}{\mathcal{e}}^{i2\Pi {\left(1-{\mathcal{e}}^{-{\left({\widehat{\Xi }}_{s}{\left(-{\text{ln}}\left(1-{\left({\overline{\overline{\mathbb{T}}}}_{{\widehat{\mathbb{H}}}_{i}}^{1}\right)}^{\pi }\right)\right)}^{\varnothing }\right)}^{\frac{1}{\varnothing }}}\right)}^{\frac{1}{\pi }}},\\ {\left({\mathcal{e}}^{-{\left({\widehat{\Xi }}_{s}{\left(-{\text{ln}}\left({\left({\overline{\overline{\mathbb{V}}}}_{{\widehat{\mathbb{H}}}_{r}}^{1}\right)}^{\pi }\right)\right)}^{\varnothing }\right)}^{\frac{1}{\varnothing }}}\right)}^{\frac{1}{\pi }} {\mathcal{e}}^{i2\Pi {\left({\mathcal{e}}^{-{\left({\widehat{\Xi }}_{s}{\left(-{\text{ln}}\left({\left({\overline{\overline{\mathbb{V}}}}_{{\widehat{\mathbb{H}}}_{a}}^{1}\right)}^{\pi }\right)\right)}^{\varnothing }\right)}^{\frac{1}{\varnothing }}}\right)}^{\frac{1}{\pi }}},\end{array}}{{\left({\mathcal{e}}^{-{\left({\widehat{\Xi }}_{s}{\left(-{\text{ln}}\left({\left({\overline{\overline{\mathbb{U}}}}_{{\widehat{\mathbb{H}}}_{r}}^{1}\right)}^{\pi }\right)\right)}^{\varnothing }\right)}^{\frac{1}{\varnothing }}}\right)}^{\frac{1}{\pi }} {\mathcal{e}}^{i2\Pi {\left({\mathcal{e}}^{-{\left({\widehat{\Xi }}_{s}{\left(-{\text{ln}}\left({\left({\overline{\overline{\mathbb{U}}}}_{{\widehat{\mathbb{H}}}_{i}}^{1}\right)}^{\pi }\right)\right)}^{\varnothing }\right)}^{\frac{1}{\varnothing }}}\right)}^{\frac{1}{\pi }}}}\end{array}\right)$$

$${\left({\widehat{\mathbb{H}}}_{p}^{1}\right)}^{{\widehat{\Xi }}_{s}}=\left(\begin{array}{c}\genfrac{}{}{0pt}{}{{\left({\mathcal{e}}^{-{\left({\widehat{\Xi }}_{s}{\left(-{\text{ln}}\left({\left({\overline{\overline{\mathbb{T}}}}_{{\widehat{\mathbb{H}}}_{r}}^{1}\right)}^{\pi }\right)\right)}^{\varnothing }\right)}^{\frac{1}{\varnothing }}}\right)}^{\frac{1}{\pi }}{\mathcal{e}}^{i2\Pi {\left({\mathcal{e}}^{-{\left({\widehat{\Xi }}_{s}{\left(-{\text{ln}}\left({\left({\overline{\overline{\mathbb{T}}}}_{{\widehat{\mathbb{H}}}_{i}}^{1}\right)}^{\pi }\right)\right)}^{\varnothing }\right)}^{\frac{1}{\varnothing }}}\right)}^{\frac{1}{\pi }}},}{{\left(1-{\mathcal{e}}^{-{\left({\widehat{\Xi }}_{s}{\left(-{\text{ln}}\left(1-{\left({\overline{\overline{\mathbb{V}}}}_{{\widehat{\mathbb{H}}}_{r}}^{1}\right)}^{\pi }\right)\right)}^{\varnothing }\right)}^{\frac{1}{\varnothing }}}\right)}^{\frac{1}{\pi }} {\mathcal{e}}^{i2\Pi {\left(1-{\mathcal{e}}^{-{\left({\widehat{\Xi }}_{s}{\left(-{\text{ln}}\left(1-{\left({\overline{\overline{\mathbb{V}}}}_{{\widehat{\mathbb{H}}}_{a}}^{1}\right)}^{\pi }\right)\right)}^{\varnothing }\right)}^{\frac{1}{\varnothing }}}\right)}^{\frac{1}{\pi }}},}\\ {\left(1-{\mathcal{e}}^{-{\left({\widehat{\Xi }}_{s}{\left(-{\text{ln}}\left(1-{\left({\overline{\overline{\mathbb{U}}}}_{{\widehat{\mathbb{H}}}_{r}}^{1}\right)}^{\pi }\right)\right)}^{\varnothing }\right)}^{\frac{1}{\varnothing }}}\right)}^{\frac{1}{\pi }} {\mathcal{e}}^{i2\Pi {\left(1-{\mathcal{e}}^{-{\left({\widehat{\Xi }}_{s}{\left(-{\text{ln}}\left(1-{\left({\overline{\overline{\mathbb{U}}}}_{{\widehat{\mathbb{H}}}_{i}}^{1}\right)}^{\pi }\right)\right)}^{\varnothing }\right)}^{\frac{1}{\varnothing }}}\right)}^{\frac{1}{\pi }}}\end{array}\right)$$

### Definition 5

^21^ Some score and accuracy values are stated below:

$${\overline{\overline{\mathcal{F}}}}_{sv\left({\widehat{\mathbb{H}}}_{p}^{1}\right)}=\frac{1}{3}\left({\overline{\overline{t}}}_{{\widehat{\mathbb{H}}}_{r}}^{1}+{\overline{\overline{t}}}_{{\widehat{\mathbb{H}}}_{i}}^{1}-{\overline{\overline{\mathbb{V}}}}_{{\widehat{\mathbb{H}}}_{r}}^{1}{-\overline{\overline{\mathbb{V}}}}_{{\widehat{\mathbb{H}}}_{f}}^{1}-{\overline{\overline{\mathbb{U}}}}_{{\widehat{\mathbb{H}}}_{r}}^{1}-{\overline{\overline{\mathbb{U}}}}_{{\widehat{\mathbb{H}}}_{i}}^{1}\right)\in \left[-\mathrm{1,1}\right]$$

$${\overline{\overline{\mathcal{F}}}}_{Av\left({\widehat{\mathbb{H}}}_{p}^{1}\right)}=\frac{1}{3}\left({\overline{\overline{t}}}_{{\widehat{\mathbb{H}}}_{r}}^{1}+{\overline{\overline{t}}}_{{\widehat{\mathbb{H}}}_{i}}^{1}+{\overline{\overline{\mathbb{V}}}}_{{\widehat{\mathbb{H}}}_{r}}^{1}{+\overline{\overline{\mathbb{V}}}}_{{\widehat{\mathbb{H}}}_{f}}^{1}-{\overline{\overline{\mathbb{U}}}}_{{\widehat{\mathbb{H}}}_{r}}^{1}-{\overline{\overline{\mathbb{U}}}}_{{\widehat{\mathbb{H}}}_{i}}^{1}\right)\in \left[{\rm O},1\right]$$

Some suitable and dominant rules are stated below:

1. When $${\overline{\overline{\mathrm{^\circ{\rm F} }}}}_{{\text{SV}}}\left({\widehat{\mathbb{H}}}_{p}^{1}\right) >{\overline{\overline{\mathrm{^\circ{\rm F} }}}}_{\mathrm{SV }}\left({\widehat{\mathbb{H}}}_{p}^{1}\right) ,$$ thus $${\widehat{\mathbb{H}}}_{p}^{1}>{\widehat{\mathbb{H}}}_{p}^{2}$$
2. When $${\overline{\overline{\mathrm{^\circ{\rm F} }}}}_{{\text{SV}}} \left({\widehat{\mathbb{H}}}_{p}^{1}\right)<{\overline{\overline{\mathrm{^\circ{\rm F} }}}}_{\mathrm{SV }}\left({\widehat{\mathbb{H}}}_{p}^{1}\right),$$ thus $${\widehat{\mathbb{H}}}_{p}^{1}<{\widehat{\mathbb{H}}}_{p}^{2}$$
3. When $${\overline{\overline{\mathrm{^\circ{\rm F} }}}}_{{\text{SV}}}\left({\widehat{\mathbb{H}}}_{p}^{1}\right) <{\overline{\overline{\mathrm{^\circ{\rm F} }}}}_{\mathrm{SV }}\left({\widehat{\mathbb{H}}}_{p}^{1}\right) ,$$ thus
4. When $${\overline{\overline{\mathrm{^\circ{\rm F} }}}}_{\mathrm{AV }} \left({\widehat{\mathbb{H}}}_{p}^{1}\right)>{\overline{\overline{\mathrm{^\circ{\rm F} }}}}_{\mathrm{AV }}\left({\widehat{\mathbb{H}}}_{p}^{2}\right),$$ thus $${\widehat{\mathbb{H}}}_{p}^{1}>{\widehat{\mathbb{H}}}_{p}^{2}$$
5. When $${\overline{\overline{\mathrm{^\circ{\rm F} }}}}_{\mathrm{AV }} \left({\widehat{\mathbb{H}}}_{p}^{1}\right) < {\overline{\overline{\mathrm{^\circ{\rm F} }}}}_{\mathrm{AV }}\left({\widehat{\mathbb{H}}}_{p}^{2}\right),$$ thus $${\widehat{\mathbb{H}}}_{p}^{1}<{\widehat{\mathbb{H}}}_{p}^{2}$$
6. When $${\overline{\overline{\mathrm{^\circ{\rm F} }}}}_{\mathrm{AV }} \left({\widehat{\mathbb{H}}}_{p}^{1}\right)={\overline{\overline{\mathrm{^\circ{\rm F} }}}}_{\mathrm{AV }}\left({\widehat{\mathbb{H}}}_{p}^{2}\right),$$ thus $${\widehat{\mathbb{H}}}_{p}^{1}={\widehat{\mathbb{H}}}_{p}^{2}$$

### Definition

^25^ The PAO is initiated in the shape:

$$PA\left({\widehat{\mathbb{H}}}_{p}^{1},{\widehat{\mathbb{H}}}_{p}^{2},\dots ,{\widehat{\mathbb{H}}}_{p}^{n}\right)=\sum_{i=1}^{n}{\widehat{\Xi }}_{i}{\widehat{\mathbb{H}}}_{p}^{i}$$

where,

$${\widehat{\Xi }}_{i}=\frac{1+T\left({\mathbb{A}}_{i}\right)}{\sum_{i=1}^{n}\left(1+T\left({\mathbb{A}}_{i}\right)\right)}$$

with $$T\left({\mathbb{A}}_{j}\right)=\sum_{\begin{array}{c}i=1\\ i\ne j\end{array}}^{n}Sup\left({\mathbb{A}}_{i},{\mathbb{A}}_{j}\right)$$ and $Sup\left({\mathbb{A}}_{i},{\mathbb{A}}_{j}\right)=1-Dis\left({\mathbb{A}}_{i},{\mathbb{A}}_{j}\right)$, such as

- $$ Sup\left({\mathbb{A}}_{i},{\mathbb{A}}_{j}\right)\in \left[\mathrm{0,1}\right]$$
- $$Sup\left({\mathbb{A}}_{i},{\mathbb{A}}_{j}\right)=Sup\left({\mathbb{A}}_{j},{\mathbb{A}}_{i}\right)$$
- If $$Sup\left({\mathbb{A}}_{i},{\mathbb{A}}_{j}\right)\le Sup\left({\mathbb{A}}_{k},{\mathbb{A}}_{l}\right)$$, then $$Dis\left({\mathbb{A}}_{i},{\mathbb{A}}_{j}\right)\ge Dis\left({\mathbb{A}}_{k},{\mathbb{A}}_{l}\right)$$.

## CTSHFSs

In this section, we investigate the theory of CTSHFS and their related information such as algebraic laws, and Aczel–Alsina operational laws.

### Definition 6

A CTSHFS ${\widehat{\mathbb{H}}}_{p}$ is initiated in the form:

$${\widehat{\mathbb{H}}}_{p}=\left\{\left(x,{(\overline{\overline{\mathbb{T}}}}_{{\widehat{\mathbb{H}}}_{p}}\left(x\right),{\overline{\overline{\mathbb{V}}}}_{{\widehat{\mathbb{H}}}_{p}}\left(x\right),{\overline{\overline{\mathbb{U}}}}_{{\widehat{\mathbb{H}}}_{p}}\left(x\right))\right):x\in {\psi }^{uni}\right\}$$

We observed in positive grade ${\overline{\overline{\mathbb{T}}}}_{{\widehat{\mathbb{H}}}_{p}}\left(x\right)=\left\{{\overline{\overline{\mathbb{T}}}}_{{\widehat{\mathbb{H}}}_{r}}^{j}\left(x\right){\mathcal{e}}^{i2\Pi \left({\overline{\overline{\mathbb{T}}}}_{{\widehat{\mathbb{H}}}_{i}}^{j}\left(x\right)\right)},j=\mathrm{1,2},\dots ,n\right\}$, abstinence grade ${\overline{\overline{\mathbb{V}}}}_{{\widehat{\mathbb{H}}}_{p}}\left(x\right)=\left\{{\overline{\overline{\mathbb{V}}}}_{{\widehat{\mathbb{H}}}_{r}}^{j}\left(x\right){\mathcal{e}}^{i2\Pi \left({\overline{\overline{\mathbb{V}}}}_{{\widehat{\mathbb{H}}}_{i}}^{j}\left(x\right)\right)},j=\mathrm{1,2},\dots ,n\right\}$, and a negative grade ${\overline{\overline{\mathbb{U}}}}_{{\widehat{\mathbb{H}}}_{p}}\left(x\right)=\left\{{\overline{\overline{\mathbb{U}}}}_{{\widehat{\mathbb{H}}}_{r}}^{j}\left(x\right){\mathcal{e}}^{i2\Pi \left({\overline{\overline{\mathbb{U}}}}_{i}^{j}\left(x\right)\right)},j=\mathrm{1,2},\dots ,n\right\}$ with the following characteristics: $\left({\left({\text{sup}}({\overline{\overline{\mathbb{T}}}}_{{\widehat{\mathbb{H}}}_{r}}^{j}\left(x\right))\right)}^{\pi }+{\left({\text{sup}}({\overline{\overline{\mathbb{V}}}}_{{\widehat{\mathbb{H}}}_{r}}^{j}\left(x\right))\right)}^{\pi }+{\left({\text{sup}}({\overline{\overline{\mathbb{U}}}}_{{\widehat{\mathbb{H}}}_{r}}^{j}\left(x\right))\right)}^{\pi },{\left({\text{sup}}({\overline{\overline{\mathbb{T}}}}_{{\widehat{\mathbb{H}}}_{i}}^{j}\left(x\right))\right)}^{\pi }+{\left({\text{sup}}{(\overline{\overline{\mathbb{V}}}}_{{\widehat{\mathbb{H}}}_{a}}^{j}\left(x\right))\right)}^{\pi }+{\left({\text{sup}}({\overline{\overline{\mathbb{U}}}}_{{\widehat{\mathbb{H}}}_{i}}^{j}\left(x\right))\right)}^{\pi }\right)\in \left[{\rm O},1\right]$, Further, we describe the main purpose of neutral grade such as: ${\overline{\overline{\mathbb{r}}}}_{{\widehat{\mathbb{H}}}_{p}}\left(x\right)=\left\{{\overline{\overline{\mathbb{r}}}}_{{\widehat{\mathbb{H}}}_{r}}^{j}\left(x\right){\mathcal{e}}^{i2\Pi \left({\overline{\overline{\mathbb{r}}}}_{{\widehat{\mathbb{H}}}_{i}}^{j}\left(x\right)\right)},j=\mathrm{1,2},\dots ,n\right\}=$

$\left\{{\left({1- (({\overline{\overline{\mathbb{T}}}}_{{\widehat{\mathbb{H}}}_{r}}^{j}\left(x\right) ) }^{\pi }+{ ({\overline{\overline{\mathbb{V}}}}_{{\widehat{\mathbb{H}}}_{r}}^{j}\left(x\right) ) }^{\pi }+{ ({\overline{\overline{\mathbb{U}}}}_{{\widehat{\mathbb{H}}}_{r}}^{j}\left(x\right) ) }^{\pi }\right)}^{\frac{1}{\pi }} {\mathcal{e}}^{{i2\Pi \left(1-\left({( {\overline{\overline{\mathbb{T}}}}_{{\widehat{\mathbb{H}}}_{i}}^{j}\left(x\right) ) }^{\pi }+({\overline{\overline{\mathbb{V}}}}_{{\widehat{\mathbb{H}}}_{a}}^{j}{\left(x\right) ) }^{\pi }+{\left({\overline{\overline{\mathbb{U}}}}_{{\widehat{\mathbb{H}}}_{i}}^{j}\left(x\right) \right)}^{\pi }\right)\right)}^{\frac{1}{\pi }} },j=\mathrm{1,2},\dots ,n\right\}$ and the purified form of the CTSHF number (CTSHFN) is deduced by : ${\widehat{\mathbb{H}}}_{p}^{j}= \left({\overline{\overline{\mathbb{T}}}}_{{\widehat{\mathbb{H}}}_{p}},{\overline{\overline{\mathbb{V}}}}_{{\widehat{\mathbb{H}}}_{p}},{\overline{\overline{\mathbb{U}}}}_{{\widehat{\mathbb{H}}}_{p}}\right)=\left(\left\{{\overline{\overline{\mathbb{T}}}}_{{\widehat{\mathbb{H}}}_{r}}^{j}{\mathcal{e}}^{i2\Pi \left({\overline{\overline{\mathbb{T}}}}_{{\widehat{\mathbb{H}}}_{i}}^{j}\right)}\right\},\left\{{\overline{\overline{\mathbb{V}}}}_{{\widehat{\mathbb{H}}}_{r}}^{j}{\mathcal{e}}^{i2\Pi \left({\overline{\overline{\mathbb{V}}}}_{{\widehat{\mathbb{H}}}_{a}}^{j}\right)}\right\},\left\{{\overline{\overline{\mathbb{U}}}}_{{\widehat{\mathbb{H}}}_{r}}^{j}{\mathcal{e}}^{i2\Pi \left({\overline{\overline{\mathbb{U}}}}_{{\widehat{\mathbb{H}}}_{i}}^{j}\right)}\right\}\right),j=\mathrm{1,2},\dots ,n.$

### Definition 7

A CTSHFN initiate

$${\widehat{\mathbb{H}}}_{p}^{j}= \left({\overline{\overline{\mathbb{T}}}}_{{\widehat{\mathbb{H}}}_{p}},{\overline{\overline{\mathbb{V}}}}_{{\widehat{\mathbb{H}}}_{p}},{\overline{\overline{\mathbb{U}}}}_{{\widehat{\mathbb{H}}}_{p}}\right)=\left(\left\{{\overline{\overline{\mathbb{T}}}}_{{\widehat{\mathbb{H}}}_{r}}^{j}{\mathcal{e}}^{i2\Pi \left({\overline{\overline{\mathbb{T}}}}_{{\widehat{\mathbb{H}}}_{i}}^{j}\right)}\right\},\left\{{\overline{\overline{\mathbb{V}}}}_{{\widehat{\mathbb{H}}}_{r}}^{j}{\mathcal{e}}^{i2\Pi \left({\overline{\overline{\mathbb{V}}}}_{{\widehat{\mathbb{H}}}_{a}}^{j}\right)}\right\},\left\{{\overline{\overline{\mathbb{U}}}}_{{\widehat{\mathbb{H}}}_{r}}^{j}{\mathcal{e}}^{i2\Pi \left({\overline{\overline{\mathbb{U}}}}_{{\widehat{\mathbb{H}}}_{i}}^{j}\right)}\right\}\right),j=\mathrm{1,2},\dots ,n.$$

we know that

$${\widehat{\upeta }}_{{\text{p}}}^{1}\oplus {\widehat{\upeta }}_{{\text{p}}}^{2}= \coprod_{\left(\begin{array}{c}{\overline{\overline{\mathbb{T}}}}_{{\widehat{\upeta }}_{{\text{r}}}}^{1},{\overline{\overline{\mathbb{T}}}}_{{\widehat{\upeta }}_{{\text{r}}}}^{2},{\overline{\overline{\mathbb{T}}}}_{{\widehat{\upeta }}_{{\text{i}}}}^{1},{\overline{\overline{\mathbb{T}}}}_{{\widehat{\upeta }}_{{\text{i}}}}^{2}\in {\overline{\overline{\mathbb{T}}}}_{{\widehat{\upeta }}_{\begin{array}{c}p\end{array}}}\\ {\overline{\overline{\mathbb{V}}}}_{{\widehat{\upeta }}_{{\text{r}}}}^{1},{\overline{\overline{\mathbb{V}}}}_{{\widehat{\upeta }}_{{\text{r}}}}^{2},{\overline{\overline{\mathbb{V}}}}_{{\widehat{\upeta }}_{{\text{a}}}}^{1},{\overline{\overline{\mathbb{V}}}}_{{\widehat{\upeta }}_{{\text{a}}}}^{2}\in {\overline{\overline{\mathbb{V}}}}_{{\widehat{\upeta }}_{\begin{array}{c}p\end{array}}}\\ {\overline{\overline{\mathbb{U}}}}_{{\widehat{\upeta }}_{{\text{r}}}}^{1},{\overline{\overline{\mathbb{U}}}}_{{\widehat{\upeta }}_{{\text{r}}}}^{2},{\overline{\overline{\mathbb{U}}}}_{{\widehat{\upeta }}_{{\text{i}}}}^{1},{\overline{\overline{\mathbb{U}}}}_{{\widehat{\upeta }}_{{\text{i}}}}^{2}\in {\overline{\overline{\mathbb{U}}}}_{{\widehat{\upeta }}_{\begin{array}{c}p\end{array}}}\end{array}\right)}\left(\begin{array}{c}{\left({ \left({\overline{\overline{\mathbb{T}}}}_{{\widehat{\upeta }}_{{\text{r}}}}^{1}\right)}^{{\text{q}}}+{ \left({\overline{\overline{\mathbb{T}}}}_{{\widehat{\upeta }}_{{\text{r}}}}^{2}\right)}^{{\text{q}}}-{ \left({\overline{\overline{\mathbb{T}}}}_{{\widehat{\upeta }}_{{\text{r}}}}^{1}\right)}^{{\text{q}}}{ \left({\overline{\overline{\mathbb{T}}}}_{{\widehat{\upeta }}_{{\text{r}}}}^{2}\right)}^{{\text{q}}}\right)}^{\frac{1}{{\text{q}}}}{\mathcal{e}}^{{{\text{i}}2\uppi \left(\left.{\left({\overline{\overline{\mathbb{T}}}}_{{\widehat{\upeta }}_{{\text{i}}}}^{1}\right) }^{{\text{q}}}+{\left({\overline{\overline{\mathbb{T}}}}_{{\widehat{\upeta }}_{{\text{i}}}}^{2}\right)}^{{\text{q}}}-{\left({\overline{\overline{\mathbb{T}}}}_{{\widehat{\upeta }}_{{\text{i}}}}^{1}\right) }^{{\text{q}}}{\left({\overline{\overline{\mathbb{T}}}}_{{\widehat{\upeta }}_{{\text{i}}}}^{2}\right) }^{{\text{q}}}\right)\right)}^{\frac{1}{{\text{q}}}} }\\ ,\left({\overline{\overline{\mathbb{V}}}}_{{\widehat{\upeta }}_{{\text{r}}}}^{1},{\overline{\overline{\mathbb{V}}}}_{{\widehat{\upeta }}_{{\text{r}}}}^{2}\right){\mathcal{e}}^{{\text{i}}2\uppi \left({\overline{\overline{\mathbb{V}}}}_{{\widehat{\upeta }}_{{\text{a}}}}^{1},{\overline{\overline{\mathbb{V}}}}_{{\widehat{\upeta }}_{{\text{a}}}}^{2}\right)},\left({\overline{\overline{\mathbb{U}}}}_{{\widehat{\upeta }}_{{\text{r}}}}^{1},{\overline{\overline{\mathbb{U}}}}_{{\widehat{\upeta }}_{{\text{r}}}}^{2}\right){\mathcal{e}}^{{\text{i}}2\uppi \left({\overline{\overline{\mathbb{U}}}}_{{\widehat{\upeta }}_{{\text{i}}}}^{1},{\overline{\overline{\mathbb{U}}}}_{{\widehat{\upeta }}_{{\text{i}}}}^{2}\right)}\end{array}\right)$$

$${\widehat{\mathbb{H}}}_{p}^{1}\otimes {\widehat{\mathbb{H}}}_{p}^{2}=\coprod_{ \left(\begin{array}{c}{\overline{\overline{\mathbb{T}}}}_{{\widehat{\mathbb{H}}}_{r}}^{1},{\overline{\overline{\mathbb{T}}}}_{{\widehat{\mathbb{H}}}_{r}}^{2},{\overline{\overline{\mathbb{T}}}}_{{\widehat{\mathbb{H}}}_{i}}^{1},{\overline{\overline{\mathbb{T}}}}_{{\widehat{\mathbb{H}}}_{i}}^{2}\in {\overline{\overline{\mathbb{T}}}}_{{\widehat{\mathbb{H}}}_{\begin{array}{c}p\end{array}}}\\ {\overline{\overline{\mathbb{V}}}}_{{\widehat{\mathbb{H}}}_{r}}^{1},{\overline{\overline{\mathbb{V}}}}_{{\widehat{\mathbb{H}}}_{r}}^{2},{\overline{\overline{\mathbb{V}}}}_{{\widehat{\mathbb{H}}}_{a}}^{1},{\overline{\overline{\mathbb{V}}}}_{{\widehat{\mathbb{H}}}_{a}}^{2}\in {\overline{\overline{\mathbb{V}}}}_{{\widehat{\mathbb{H}}}_{\begin{array}{c}p\end{array}}}\\ {\overline{\overline{\mathbb{U}}}}_{{\widehat{\mathbb{H}}}_{r}}^{1},{\overline{\overline{\mathbb{U}}}}_{{\widehat{\mathbb{H}}}_{r}}^{2},{\overline{\overline{\mathbb{U}}}}_{{\widehat{\mathbb{H}}}_{i}}^{1},{\overline{\overline{\mathbb{U}}}}_{{\widehat{\mathbb{H}}}_{i}}^{2}\in {\overline{\overline{\mathbb{U}}}}_{{\widehat{\mathbb{H}}}_{\begin{array}{c}p\end{array}}}\end{array}\right)}\left(\begin{array}{c}\left({\overline{\overline{\mathbb{T}}}}_{{\widehat{\mathbb{H}}}_{r}}^{1},{\overline{\overline{\mathbb{T}}}}_{{\widehat{\mathbb{H}}}_{r}}^{2}\right){\mathcal{e}}^{i2\Pi \left({\overline{\overline{\mathbb{T}}}}_{{\widehat{\mathbb{H}}}_{i}}^{1},{\overline{\overline{\mathbb{T}}}}_{{\widehat{\mathbb{H}}}_{i}}^{2}\right)},\\ \begin{array}{c}{\left({ \left({\overline{\overline{\overline{\mathbb{V}}}} }_{{\widehat{\mathbb{H}}}_{r}}^{1}\right)}^{\pi }+{ \left({\overline{\overline{\overline{\mathbb{V}}}} }_{{\widehat{\mathbb{H}}}_{r}}^{2}\right)}^{\pi }-{ \left({\overline{\overline{\overline{\mathbb{V}}}} }_{{\widehat{\mathbb{H}}}_{r}}^{1}\right)}^{\pi }{ \left({\overline{\overline{\overline{\mathbb{V}}}} }_{{\widehat{\mathbb{H}}}_{r}}^{2}\right)}^{\pi }\right)}^{\frac{1}{\pi }}{\mathcal{e}}^{{i2\Pi \left(\left.{\left({\overline{\overline{\overline{\mathbb{V}}}} }_{{\widehat{\mathbb{H}}}_{i}}^{1}\right) }^{\pi }+{\left({\overline{\overline{\overline{\mathbb{V}}}} }_{{\widehat{\mathbb{H}}}_{i}}^{2}\right)}^{\pi }-{\left({\overline{\overline{\overline{\mathbb{V}}}} }_{{\widehat{\mathbb{H}}}_{i}}^{1}\right) }^{\pi }{\left({\overline{\overline{\overline{\mathbb{V}}}} }_{{\widehat{\mathbb{H}}}_{i}}^{2}\right) }^{\pi }\right)\right)}^{\frac{1}{\pi }} },\\ {\left({ \left({\overline{\overline{\overline{\mathbb{U}}}} }_{{\widehat{\mathbb{H}}}_{r}}^{1}\right)}^{\pi }+{ \left({\overline{\overline{\overline{\mathbb{U}}}} }_{{\widehat{\mathbb{H}}}_{r}}^{2}\right)}^{\pi }-{ \left({\overline{\overline{\overline{\mathbb{U}}}} }_{{\widehat{\mathbb{H}}}_{r}}^{1}\right)}^{\pi }{ \left({\overline{\overline{\overline{\mathbb{U}}}} }_{{\widehat{\mathbb{H}}}_{r}}^{2}\right)}^{\pi }\right)}^{\frac{1}{\pi }}{\mathcal{e}}^{{i2\Pi \left(\left.{\left({\overline{\overline{\overline{\mathbb{U}}}} }_{{\widehat{\mathbb{H}}}_{i}}^{1}\right) }^{\pi }+{\left({\overline{\overline{\overline{\mathbb{U}}}} }_{{\widehat{\mathbb{H}}}_{i}}^{2}\right)}^{\pi }-{\left({\overline{\overline{\overline{\mathbb{U}}}} }_{{\widehat{\mathbb{H}}}_{i}}^{1}\right) }^{\pi }{\left({\overline{\overline{\overline{\mathbb{U}}}} }_{{\widehat{\mathbb{H}}}_{i}}^{2}\right) }^{\pi }\right)\right)}^{\frac{1}{\pi }}}\end{array}\end{array}\right)$$

$${\widehat{\Xi }}_{s}{\widehat{\mathbb{H}}}_{p}^{1}=\coprod_{\left(\begin{array}{c}{\overline{\overline{\mathbb{T}}}}_{{\widehat{\mathbb{H}}}_{r}}^{1},{\overline{\overline{\mathbb{T}}}}_{{\widehat{\mathbb{H}}}_{i}}^{1}\in {\overline{\overline{\mathbb{T}}}}_{{\widehat{\mathbb{H}}}_{\begin{array}{c}p\end{array}}}\\ {\overline{\overline{\mathbb{V}}}}_{{\widehat{\mathbb{H}}}_{r}}^{1},{\overline{\overline{\mathbb{V}}}}_{{\widehat{\mathbb{H}}}_{a}}^{1}\in {\overline{\overline{\mathbb{V}}}}_{{\widehat{\mathbb{H}}}_{\begin{array}{c}p\end{array}}}\\ {\overline{\overline{\mathbb{U}}}}_{{\widehat{\mathbb{H}}}_{r}}^{1},{\overline{\overline{\mathbb{U}}}}_{{\widehat{\mathbb{H}}}_{i}}^{1}\in {\overline{\overline{\mathbb{U}}}}_{{\widehat{\mathbb{H}}}_{\begin{array}{c}p\end{array}}}\end{array}\right)}\left(\begin{array}{c}{\left(1-{\left(1-{\left({\overline{\overline{\mathbb{T}}}}_{{\widehat{\mathbb{H}}}_{r}}^{1}\right)}^{\pi }\right)}^{{\widehat{\Xi }}_{s}}\right)}^{\frac{1}{\pi }}{\mathcal{e}}^{i2\Pi \left({\left(1-{\left(1-{\left({\overline{\overline{\mathbb{T}}}}_{{\widehat{\mathbb{H}}}_{i}}^{1}\right)}^{\pi }\right)}^{{\widehat{\Xi }}_{s}}\right)}^{\frac{1}{\pi }}\right)},{\left({\overline{\overline{\mathbb{V}}}}_{{\widehat{\mathbb{H}}}_{r}}^{1}\right)}^{{\widehat{\Xi }}_{s}}{\mathcal{e}}^{i2\Pi {\left({\overline{\overline{\mathbb{V}}}}_{{\widehat{\mathbb{H}}}_{a}}^{1}\right)}^{{\widehat{\Xi }}_{s}}}\\ ,{\left({\overline{\overline{\mathbb{U}}}}_{{\widehat{\mathbb{H}}}_{r}}^{1}\right)}^{{\widehat{\Xi }}_{s}}{\mathcal{e}}^{i2\Pi {\left({\overline{\overline{\mathbb{U}}}}_{{\widehat{\mathbb{H}}}_{i}}^{1}\right)}^{{\widehat{\Xi }}_{s}}}\end{array}\right)$$

$${\left({\widehat{\mathbb{H}}}_{p}^{1}\right)}^{{\widehat{\Xi }}_{s}}=\coprod_{\left(\begin{array}{c}{\overline{\overline{\mathbb{T}}}}_{{\widehat{\mathbb{H}}}_{r}}^{1},{\overline{\overline{\mathbb{T}}}}_{{\widehat{\mathbb{H}}}_{i}}^{1}\in {\overline{\overline{\mathbb{T}}}}_{{\widehat{\mathbb{H}}}_{\begin{array}{c}p\end{array}}}\\ {\overline{\overline{\mathbb{V}}}}_{{\widehat{\mathbb{H}}}_{r}}^{1},{\overline{\overline{\mathbb{V}}}}_{{\widehat{\mathbb{H}}}_{a}}^{1}\in {\overline{\overline{\mathbb{V}}}}_{{\widehat{\mathbb{H}}}_{\begin{array}{c}p\end{array}}}\\ {\overline{\overline{\mathbb{U}}}}_{{\widehat{\mathbb{H}}}_{r}}^{1},{\overline{\overline{\mathbb{U}}}}_{{\widehat{\mathbb{H}}}_{i}}^{1}\in {\overline{\overline{\mathbb{U}}}}_{{\widehat{\mathbb{H}}}_{\begin{array}{c}p\end{array}}}\end{array}\right)}\left(\begin{array}{c}{\left({\overline{\overline{\mathbb{T}}}}_{{\widehat{\mathbb{H}}}_{r}}^{1}\right)}^{{\widehat{\Xi }}_{s}}{\mathcal{e}}^{i2\Pi {\left({\overline{\overline{\mathbb{T}}}}_{{\widehat{\mathbb{H}}}_{i}}^{1}\right)}^{{\widehat{\Xi }}_{s}}},{\left(1-{\left(1-{\left({\overline{\overline{\mathbb{V}}}}_{{\widehat{\mathbb{H}}}_{r}}^{1}\right)}^{\pi }\right)}^{{\widehat{\Xi }}_{s}}\right)}^{\frac{1}{\pi }}{\mathcal{e}}^{i2\Pi \left({\left(1-{\left(1-{\left({\overline{\overline{\mathbb{V}}}}_{{\widehat{\mathbb{H}}}_{a}}^{1}\right)}^{\pi }\right)}^{{\widehat{\Xi }}_{s}}\right)}^{\frac{1}{\pi }}\right)}\\ {\left(1-{\left(1-{\left({\overline{\overline{\mathbb{U}}}}_{{\widehat{\mathbb{H}}}_{r}}^{1}\right)}^{\pi }\right)}^{{\widehat{\Xi }}_{s}}\right)}^{\frac{1}{\pi }}{\mathcal{e}}^{i2\Pi \left({\left(1-{\left(1-{\left({\overline{\overline{\mathbb{U}}}}_{{\widehat{\mathbb{H}}}_{i}}^{1}\right)}^{\pi }\right)}^{{\widehat{\Xi }}_{s}}\right)}^{\frac{1}{\pi }}\right)}\end{array}\right)$$

### Definition 8

Under the presence of CTSHFNs ${\widehat{\mathbb{H}}}_{p}^{j}= \left({\overline{\overline{\mathbb{T}}}}_{{\widehat{\mathbb{H}}}_{p}},{\overline{\overline{\mathbb{V}}}}_{{\widehat{\mathbb{H}}}_{p}},{\overline{\overline{\mathbb{U}}}}_{{\widehat{\mathbb{H}}}_{p}}\right)=\left(\left\{{\overline{\overline{\mathbb{T}}}}_{{\widehat{\mathbb{H}}}_{r}}^{j}{\mathcal{e}}^{i2\Pi \left({\overline{\overline{\mathbb{T}}}}_{{\widehat{\mathbb{H}}}_{i}}^{j}\right)}\right\},\left\{{\overline{\overline{\mathbb{V}}}}_{{\widehat{\mathbb{H}}}_{r}}^{j}{\mathcal{e}}^{i2\Pi \left({\overline{\overline{\mathbb{V}}}}_{{\widehat{\mathbb{H}}}_{a}}^{j}\right)}\right\},\left\{{\overline{\overline{\mathbb{U}}}}_{{\widehat{\mathbb{H}}}_{r}}^{j}{\mathcal{e}}^{i2\Pi \left({\overline{\overline{\mathbb{U}}}}_{{\widehat{\mathbb{H}}}_{i}}^{j}\right)}\right\}\right),j=\mathrm{1,2},\dots ,n.$ we have

$${\widehat{\upeta }}_{{\text{p}}}^{1}\oplus {\widehat{\upeta }}_{{\text{p}}}^{2}= \coprod_{\left(\begin{array}{c}{\overline{\overline{\mathbb{T}}}}_{{\widehat{\upeta }}_{{\text{r}}}}^{1},{\overline{\overline{\mathbb{T}}}}_{{\widehat{\upeta }}_{{\text{r}}}}^{2},{\overline{\overline{\mathbb{T}}}}_{{\widehat{\upeta }}_{{\text{i}}}}^{1},{\overline{\overline{\mathbb{T}}}}_{{\widehat{\upeta }}_{{\text{i}}}}^{2}\in {\overline{\overline{\mathbb{T}}}}_{{\widehat{\upeta }}_{\begin{array}{c}p\end{array}}}\\ {\overline{\overline{\mathbb{V}}}}_{{\widehat{\upeta }}_{{\text{r}}}}^{1},{\overline{\overline{\mathbb{V}}}}_{{\widehat{\upeta }}_{{\text{r}}}}^{2},{\overline{\overline{\mathbb{V}}}}_{{\widehat{\upeta }}_{{\text{a}}}}^{1},{\overline{\overline{\mathbb{V}}}}_{{\widehat{\upeta }}_{{\text{a}}}}^{2}\in {\overline{\overline{\mathbb{V}}}}_{{\widehat{\upeta }}_{\begin{array}{c}p\end{array}}}\\ {\overline{\overline{\mathbb{U}}}}_{{\widehat{\upeta }}_{{\text{r}}}}^{1},{\overline{\overline{\mathbb{U}}}}_{{\widehat{\upeta }}_{{\text{r}}}}^{2},{\overline{\overline{\mathbb{U}}}}_{{\widehat{\upeta }}_{{\text{i}}}}^{1},{\overline{\overline{\mathbb{U}}}}_{{\widehat{\upeta }}_{{\text{i}}}}^{2}\in {\overline{\overline{\mathbb{U}}}}_{{\widehat{\upeta }}_{\begin{array}{c}p\end{array}}}\end{array}\right)}\left(\begin{array}{c}\genfrac{}{}{0pt}{}{{\left(1-{\mathcal{e}}^{-{\left({(-{\text{ln}}\left(1-{\left({\overline{\overline{\mathbb{T}}}}_{{\widehat{\mathbb{H}}}_{r}}^{1}\right)}^{\pi }\right)}^{\varnothing }+{\left(-{\text{ln}}\left(1-{\left({\overline{\overline{\mathbb{T}}}}_{{\widehat{\mathbb{H}}}_{r}}^{2}\right)}^{\pi }\right)\right)}^{\varnothing }\right)}^{\frac{1}{\varnothing }}}\right)}^{\frac{1}{\pi }}{\mathcal{e}}^{i2\Pi {\left(1-{\mathcal{e}}^{-{\left({(-{\text{ln}}\left(1-{\left({\overline{\overline{\mathbb{T}}}}_{{\widehat{\mathbb{H}}}_{i}}^{1}\right)}^{\pi }\right)}^{\varnothing }+{\left(-{\text{ln}}\left(1-{\left({\overline{\overline{\mathbb{T}}}}_{{\widehat{\mathbb{H}}}_{i}}^{2}\right)}^{\pi }\right)\right)}^{\varnothing }\right)}^{\frac{1}{\varnothing }}}\right)}^{\frac{1}{\pi }}},}{{\left({\mathcal{e}}^{-{\left({(-{\text{ln}}\left({\left({\overline{\overline{\mathbb{V}}}}_{{\widehat{\mathbb{H}}}_{r}}^{1}\right)}^{\pi }\right)}^{\varnothing }+{\left(-{\text{ln}}\left({\left({\overline{\overline{\mathbb{V}}}}_{{\widehat{\mathbb{H}}}_{r}}^{2}\right)}^{\pi }\right)\right)}^{\varnothing }\right)}^{\frac{1}{\varnothing }}}\right)}^{\frac{1}{\pi }} {\mathcal{e}}^{i2\Pi {\left({\mathcal{e}}^{-{\left({(-{\text{ln}}\left({\left({\overline{\overline{\mathbb{V}}}}_{{\widehat{\mathbb{H}}}_{a}}^{1}\right)}^{\pi }\right)}^{\varnothing }+{\left(-{\text{ln}}\left({\left({\overline{\overline{\mathbb{V}}}}_{{\widehat{\mathbb{H}}}_{a}}^{2}\right)}^{\pi }\right)\right)}^{\varnothing }\right)}^{\frac{1}{\varnothing }}}\right)}^{\frac{1}{\pi }}},}\\ {\left({\mathcal{e}}^{-{\left({(-{\text{ln}}\left({\left({\overline{\overline{\mathbb{U}}}}_{{\widehat{\mathbb{H}}}_{r}}^{1}\right)}^{\pi }\right)}^{\varnothing }+{\left(-{\text{ln}}\left({\left({\overline{\overline{\mathbb{U}}}}_{{\widehat{\mathbb{H}}}_{r}}^{2}\right)}^{\pi }\right)\right)}^{\varnothing }\right)}^{\frac{1}{\varnothing }}}\right)}^{\frac{1}{\pi }} {\mathcal{e}}^{i2\Pi {\left({\mathcal{e}}^{-{\left({(-{\text{ln}}\left({\left({\overline{\overline{\mathbb{U}}}}_{{\widehat{\mathbb{H}}}_{i}}^{1}\right)}^{\pi }\right)}^{\varnothing }+{\left(-{\text{ln}}\left({\left({\overline{\overline{\mathbb{U}}}}_{{\widehat{\mathbb{H}}}_{i}}^{2}\right)}^{\pi }\right)\right)}^{\varnothing }\right)}^{\frac{1}{\varnothing }}}\right)}^{\frac{1}{\pi }}}\end{array}\right)$$

$${\widehat{\mathbb{H}}}_{p}^{1}\otimes {\widehat{\mathbb{H}}}_{p}^{2}=\coprod_{\left(\begin{array}{c}{\overline{\overline{\mathbb{T}}}}_{{\widehat{\upeta }}_{{\text{r}}}}^{1},{\overline{\overline{\mathbb{T}}}}_{{\widehat{\upeta }}_{{\text{r}}}}^{2},{\overline{\overline{\mathbb{T}}}}_{{\widehat{\upeta }}_{{\text{i}}}}^{1},{\overline{\overline{\mathbb{T}}}}_{{\widehat{\upeta }}_{{\text{i}}}}^{2}\in {\overline{\overline{\mathbb{T}}}}_{{\widehat{\upeta }}_{\begin{array}{c}p\end{array}}}\\ {\overline{\overline{\mathbb{V}}}}_{{\widehat{\upeta }}_{{\text{r}}}}^{1},{\overline{\overline{\mathbb{V}}}}_{{\widehat{\upeta }}_{{\text{r}}}}^{2},{\overline{\overline{\mathbb{V}}}}_{{\widehat{\upeta }}_{{\text{a}}}}^{1},{\overline{\overline{\mathbb{V}}}}_{{\widehat{\upeta }}_{{\text{a}}}}^{2}\in {\overline{\overline{\mathbb{V}}}}_{{\widehat{\upeta }}_{\begin{array}{c}p\end{array}}}\\ {\overline{\overline{\mathbb{U}}}}_{{\widehat{\upeta }}_{{\text{r}}}}^{1},{\overline{\overline{\mathbb{U}}}}_{{\widehat{\upeta }}_{{\text{r}}}}^{2},{\overline{\overline{\mathbb{U}}}}_{{\widehat{\upeta }}_{{\text{i}}}}^{1},{\overline{\overline{\mathbb{U}}}}_{{\widehat{\upeta }}_{{\text{i}}}}^{2}\in {\overline{\overline{\mathbb{U}}}}_{{\widehat{\upeta }}_{\begin{array}{c}p\end{array}}}\end{array}\right)}\left(\begin{array}{c}\genfrac{}{}{0pt}{}{{\left({\mathcal{e}}^{-{\left({(-{\text{ln}}\left({\left({\overline{\overline{\mathbb{T}}}}_{{\widehat{\mathbb{H}}}_{r}}^{1}\right)}^{\pi }\right)}^{\varnothing }+{\left(-{\text{ln}}\left({\left({\overline{\overline{\mathbb{T}}}}_{{\widehat{\mathbb{H}}}_{r}}^{2}\right)}^{\pi }\right)\right)}^{\varnothing }\right)}^{\frac{1}{\varnothing }}}\right)}^{\frac{1}{\pi }}{\mathcal{e}}^{i2\Pi {\left({\mathcal{e}}^{-{\left({(-{\text{ln}}\left({\left({\overline{\overline{\mathbb{T}}}}_{{\widehat{\mathbb{H}}}_{i}}^{1}\right)}^{\pi }\right)}^{\varnothing }+{\left(-{\text{ln}}\left({\left({\overline{\overline{\mathbb{T}}}}_{{\widehat{\mathbb{H}}}_{i}}^{2}\right)}^{\pi }\right)\right)}^{\varnothing }\right)}^{\frac{1}{\varnothing }}}\right)}^{\frac{1}{\pi }}},}{{\left(1-{\mathcal{e}}^{-{\left({(-{\text{ln}}\left(1-{\left({\overline{\overline{\mathbb{V}}}}_{{\widehat{\mathbb{H}}}_{r}}^{1}\right)}^{\pi }\right)}^{\varnothing }+{\left(-{\text{ln}}\left(1-{\left({\overline{\overline{\mathbb{V}}}}_{{\widehat{\mathbb{H}}}_{r}}^{2}\right)}^{\pi }\right)\right)}^{\varnothing }\right)}^{\frac{1}{\varnothing }}}\right)}^{\frac{1}{\pi }} {\mathcal{e}}^{i2\Pi {\left({1-\mathcal{e}}^{-{\left({(-{\text{ln}}\left({1-\left({\overline{\overline{\mathbb{V}}}}_{{\widehat{\mathbb{H}}}_{a}}^{1}\right)}^{\pi }\right)}^{\varnothing }+{\left(-{\text{ln}}\left({1-\left({\overline{\overline{\mathbb{V}}}}_{{\widehat{\mathbb{H}}}_{a}}^{2}\right)}^{\pi }\right)\right)}^{\varnothing }\right)}^{\frac{1}{\varnothing }}}\right)}^{\frac{1}{\pi }}},}\\ {\left(1-{\mathcal{e}}^{-{\left({(-{\text{ln}}\left(1-{\left({\overline{\overline{\mathbb{U}}}}_{{\widehat{\mathbb{H}}}_{r}}^{1}\right)}^{\pi }\right)}^{\varnothing }+{\left(-{\text{ln}}\left({1-\left({\overline{\overline{\mathbb{U}}}}_{{\widehat{\mathbb{H}}}_{r}}^{2}\right)}^{\pi }\right)\right)}^{\varnothing }\right)}^{\frac{1}{\varnothing }}}\right)}^{\frac{1}{\pi }} {\mathcal{e}}^{i2\Pi {\left({1-\mathcal{e}}^{-{\left({(-{\text{ln}}\left(1-{\left({\overline{\overline{\mathbb{U}}}}_{{\widehat{\mathbb{H}}}_{i}}^{1}\right)}^{\pi }\right)}^{\varnothing }+{\left(-{\text{ln}}\left(1-{\left({\overline{\overline{\mathbb{U}}}}_{{\widehat{\mathbb{H}}}_{i}}^{2}\right)}^{\pi }\right)\right)}^{\varnothing }\right)}^{\frac{1}{\varnothing }}}\right)}^{\frac{1}{\pi }}}\end{array}\right)$$

$${\widehat{\Xi }}_{s}{\widehat{\mathbb{H}}}_{p}^{1}=\coprod_{\left(\begin{array}{c}{\overline{\overline{\mathbb{T}}}}_{{\widehat{\mathbb{H}}}_{r}}^{1},{\overline{\overline{\mathbb{T}}}}_{{\widehat{\mathbb{H}}}_{i}}^{1}\in {\overline{\overline{\mathbb{T}}}}_{{\widehat{\mathbb{H}}}_{\begin{array}{c}p\end{array}}}\\ {\overline{\overline{\mathbb{V}}}}_{{\widehat{\mathbb{H}}}_{r}}^{1},{\overline{\overline{\mathbb{V}}}}_{{\widehat{\mathbb{H}}}_{a}}^{1}\in {\overline{\overline{\mathbb{V}}}}_{{\widehat{\mathbb{H}}}_{\begin{array}{c}p\end{array}}}\\ {\overline{\overline{\mathbb{U}}}}_{{\widehat{\mathbb{H}}}_{r}}^{1},{\overline{\overline{\mathbb{U}}}}_{{\widehat{\mathbb{H}}}_{i}}^{1}\in {\overline{\overline{\mathbb{U}}}}_{{\widehat{\mathbb{H}}}_{\begin{array}{c}p\end{array}}}\end{array}\right)}\left(\begin{array}{c}\genfrac{}{}{0pt}{}{\begin{array}{c}{\left(1-{\mathcal{e}}^{-{\left({\widehat{\Xi }}_{s}{\left(-{\text{ln}}\left(1-{\left({\overline{\overline{\mathbb{T}}}}_{{\widehat{\mathbb{H}}}_{r}}^{1}\right)}^{\pi }\right)\right)}^{\varnothing }\right)}^{\frac{1}{\varnothing }}}\right)}^{\frac{1}{\pi }}{\mathcal{e}}^{i2\Pi {\left(1-{\mathcal{e}}^{-{\left({\widehat{\Xi }}_{s}{\left(-{\text{ln}}\left(1-{\left({\overline{\overline{\mathbb{T}}}}_{{\widehat{\mathbb{H}}}_{i}}^{1}\right)}^{\pi }\right)\right)}^{\varnothing }\right)}^{\frac{1}{\varnothing }}}\right)}^{\frac{1}{\pi }}},\\ {\left({\mathcal{e}}^{-{\left({\widehat{\Xi }}_{s}{\left(-{\text{ln}}\left({\left({\overline{\overline{\mathbb{V}}}}_{{\widehat{\mathbb{H}}}_{r}}^{1}\right)}^{\pi }\right)\right)}^{\varnothing }\right)}^{\frac{1}{\varnothing }}}\right)}^{\frac{1}{\pi }} {\mathcal{e}}^{i2\Pi {\left({\mathcal{e}}^{-{\left({\widehat{\Xi }}_{s}{\left(-{\text{ln}}\left({\left({\overline{\overline{\mathbb{V}}}}_{{\widehat{\mathbb{H}}}_{a}}^{1}\right)}^{\pi }\right)\right)}^{\varnothing }\right)}^{\frac{1}{\varnothing }}}\right)}^{\frac{1}{\pi }}},\end{array}}{{\left({\mathcal{e}}^{-{\left({\widehat{\Xi }}_{s}{\left(-{\text{ln}}\left({\left({\overline{\overline{\mathbb{U}}}}_{{\widehat{\mathbb{H}}}_{r}}^{1}\right)}^{\pi }\right)\right)}^{\varnothing }\right)}^{\frac{1}{\varnothing }}}\right)}^{\frac{1}{\pi }} {\mathcal{e}}^{i2\Pi {\left({\mathcal{e}}^{-{\left({\widehat{\Xi }}_{s}{\left(-{\text{ln}}\left({\left({\overline{\overline{\mathbb{U}}}}_{{\widehat{\mathbb{H}}}_{i}}^{1}\right)}^{\pi }\right)\right)}^{\varnothing }\right)}^{\frac{1}{\varnothing }}}\right)}^{\frac{1}{\pi }}}}\end{array}\right)$$

$${\left({\widehat{\mathbb{H}}}_{p}^{1}\right)}^{{\widehat{\Xi }}_{s}}=\coprod_{\left(\begin{array}{c}{\overline{\overline{\mathbb{T}}}}_{{\widehat{\mathbb{H}}}_{r}}^{1},{\overline{\overline{\mathbb{T}}}}_{{\widehat{\mathbb{H}}}_{i}}^{1}\in {\overline{\overline{\mathbb{T}}}}_{{\widehat{\mathbb{H}}}_{\begin{array}{c}p\end{array}}}\\ {\overline{\overline{\mathbb{V}}}}_{{\widehat{\mathbb{H}}}_{r}}^{1},{\overline{\overline{\mathbb{V}}}}_{{\widehat{\mathbb{H}}}_{a}}^{1}\in {\overline{\overline{\mathbb{V}}}}_{{\widehat{\mathbb{H}}}_{\begin{array}{c}p\end{array}}}\\ {\overline{\overline{\mathbb{U}}}}_{{\widehat{\mathbb{H}}}_{r}}^{1},{\overline{\overline{\mathbb{U}}}}_{{\widehat{\mathbb{H}}}_{i}}^{1}\in {\overline{\overline{\mathbb{U}}}}_{{\widehat{\mathbb{H}}}_{\begin{array}{c}p\end{array}}}\end{array}\right)}\left(\begin{array}{c}\genfrac{}{}{0pt}{}{{\left({\mathcal{e}}^{-{\left({\widehat{\Xi }}_{s}{\left(-{\text{ln}}\left({\left({\overline{\overline{\mathbb{T}}}}_{{\widehat{\mathbb{H}}}_{r}}^{1}\right)}^{\pi }\right)\right)}^{\varnothing }\right)}^{\frac{1}{\varnothing }}}\right)}^{\frac{1}{\pi }}{\mathcal{e}}^{i2\Pi {\left({\mathcal{e}}^{-{\left({\widehat{\Xi }}_{s}{\left(-{\text{ln}}\left({\left({\overline{\overline{\mathbb{T}}}}_{{\widehat{\mathbb{H}}}_{i}}^{1}\right)}^{\pi }\right)\right)}^{\varnothing }\right)}^{\frac{1}{\varnothing }}}\right)}^{\frac{1}{\pi }}},}{{\left(1-{\mathcal{e}}^{-{\left({\widehat{\Xi }}_{s}{\left(-{\text{ln}}\left(1-{\left({\overline{\overline{\mathbb{V}}}}_{{\widehat{\mathbb{H}}}_{r}}^{1}\right)}^{\pi }\right)\right)}^{\varnothing }\right)}^{\frac{1}{\varnothing }}}\right)}^{\frac{1}{\pi }} {\mathcal{e}}^{i2\Pi {\left(1-{\mathcal{e}}^{-{\left({\widehat{\Xi }}_{s}{\left(-{\text{ln}}\left(1-{\left({\overline{\overline{\mathbb{V}}}}_{{\widehat{\mathbb{H}}}_{a}}^{1}\right)}^{\pi }\right)\right)}^{\varnothing }\right)}^{\frac{1}{\varnothing }}}\right)}^{\frac{1}{\pi }}},}\\ {\left(1-{\mathcal{e}}^{-{\left({\widehat{\Xi }}_{s}{\left(-{\text{ln}}\left(1-{\left({\overline{\overline{\mathbb{U}}}}_{{\widehat{\mathbb{H}}}_{r}}^{1}\right)}^{\pi }\right)\right)}^{\varnothing }\right)}^{\frac{1}{\varnothing }}}\right)}^{\frac{1}{\pi }} {\mathcal{e}}^{i2\Pi {\left(1-{\mathcal{e}}^{-{\left({\widehat{\Xi }}_{s}{\left(-{\text{ln}}\left(1-{\left({\overline{\overline{\mathbb{U}}}}_{{\widehat{\mathbb{H}}}_{i}}^{1}\right)}^{\pi }\right)\right)}^{\varnothing }\right)}^{\frac{1}{\varnothing }}}\right)}^{\frac{1}{\pi }}}\end{array}\right)$$

### Definition 9

Under the presence of CTSFNs ${\widehat{\mathbb{H}}}_{p}^{j}= \left({\overline{\overline{\mathbb{T}}}}_{{\widehat{\mathbb{H}}}_{p}},{\overline{\overline{\mathbb{V}}}}_{{\widehat{\mathbb{H}}}_{p}},{\overline{\overline{\mathbb{U}}}}_{{\widehat{\mathbb{H}}}_{p}}\right)=\left(\left\{{\overline{\overline{\mathbb{T}}}}_{{\widehat{\mathbb{H}}}_{r}}^{j}{\mathcal{e}}^{i2\Pi \left({\overline{\overline{\mathbb{T}}}}_{{\widehat{\mathbb{H}}}_{i}}^{j}\right)}\right\},\left\{{\overline{\overline{\mathbb{V}}}}_{{\widehat{\mathbb{H}}}_{r}}^{j}{\mathcal{e}}^{i2\Pi \left({\overline{\overline{\mathbb{V}}}}_{{\widehat{\mathbb{H}}}_{a}}^{j}\right)}\right\},\left\{{\overline{\overline{\mathbb{U}}}}_{{\widehat{\mathbb{H}}}_{r}}^{j}{\mathcal{e}}^{i2\Pi \left({\overline{\overline{\mathbb{U}}}}_{{\widehat{\mathbb{H}}}_{i}}^{j}\right)}\right\}\right),j=\mathrm{1,2},\dots ,n.$ we have

$${\overline{\overline{\mathrm{^\circ{\rm F} }}}}_{{\text{SV}}}\left({\widehat{\mathbb{H}}}_{p}^{1}\right)=\frac{1}{6}\left(\frac{1}{n}\sum_{j=1}^{n}{\overline{\overline{t}}}_{{\widehat{\mathbb{H}}}_{r}}^{j}+\frac{1}{n}\sum_{j=1}^{n}{\overline{\overline{t}}}_{{\widehat{\mathbb{H}}}_{i}}^{j}-\frac{1}{n}\sum_{j=1}^{n}{\overline{\overline{h}}}_{{\widehat{\mathbb{H}}}_{r}}^{j}-\frac{1}{n}\sum_{j=1}^{n}{\overline{\overline{h}}}_{{\widehat{\mathbb{H}}}_{a}}^{j}-\frac{1}{n}\sum_{j=1}^{n}{\overline{\overline{f}}}_{{\widehat{\mathbb{H}}}_{r}}^{j}-\frac{1}{n}\sum_{j=1}^{n}{\overline{\overline{f}}}_{{\widehat{\mathbb{H}}}_{i}}^{j}\right) \epsilon \left[-\mathrm{1,1}\right]$$

$${\overline{\overline{\mathrm{^\circ{\rm F} }}}}_{{\text{AV}}}\left({\widehat{\mathbb{H}}}_{p}^{1}\right)=\frac{1}{6}\left(\frac{1}{n}\sum_{j=1}^{n}{\overline{\overline{t}}}_{{\widehat{\mathbb{H}}}_{r}}^{j}+\frac{1}{n}\sum_{j=1}^{n}{\overline{\overline{t}}}_{{\widehat{\mathbb{H}}}_{i}}^{j}+\frac{1}{n}\sum_{j=1}^{n}{\overline{\overline{h}}}_{{\widehat{\mathbb{H}}}_{r}}^{j}+\frac{1}{n}\sum_{j=1}^{n}{\overline{\overline{h}}}_{{\widehat{\mathbb{H}}}_{a}}^{j}+\frac{1}{n}\sum_{j=1}^{n}{\overline{\overline{f}}}_{{\widehat{\mathbb{H}}}_{r}}^{j}+\frac{1}{n}\sum_{j=1}^{n}{\overline{\overline{f}}}_{{\widehat{\mathbb{H}}}_{i}}^{j}\right) \epsilon \left[{\rm O},1\right]$$

Under the consideration of data in Eq. (22) and Eq. (23), we express few properties, such as:

1. When $${\overline{\overline{\mathrm{^\circ{\rm F} }}}}_{{\text{SV}}}\left({\widehat{\mathbb{H}}}_{p}^{1}\right) >{\overline{\overline{\mathrm{^\circ{\rm F} }}}}_{{\text{SV}}}\left({\widehat{\mathbb{H}}}_{p}^{2}\right),$$ thus $${\widehat{\mathbb{H}}}_{p}^{1}>{\widehat{\mathbb{H}}}_{p}^{2}$$
2. When $${\overline{\overline{\mathrm{^\circ{\rm F} }}}}_{{\text{SV}}}\left({\widehat{\mathbb{H}}}_{p}^{1}\right) <{\overline{\overline{\mathrm{^\circ{\rm F} }}}}_{{\text{SV}}}\left({\widehat{\mathbb{H}}}_{p}^{2}\right),$$ thus $${\widehat{\mathbb{H}}}_{p}^{1}<{\widehat{\mathbb{H}}}_{p}^{2}$$
3. When $${\overline{\overline{\mathrm{^\circ{\rm F} }}}}_{{\text{SV}}}\left({\widehat{\mathbb{H}}}_{p}^{1}\right) <{\overline{\overline{\mathrm{^\circ{\rm F} }}}}_{{\text{SV}}}\left({\widehat{\mathbb{H}}}_{p}^{2}\right),$$ thus
4. when $${\overline{\overline{\mathrm{^\circ{\rm F} }}}}_{{\text{AV}}}\left({\widehat{\mathbb{H}}}_{p}^{1}\right) >{\overline{\overline{\mathrm{^\circ{\rm F} }}}}_{{\text{AV}}}\left({\widehat{\mathbb{H}}}_{p}^{2}\right),$$ thus $${\widehat{\mathbb{H}}}_{p}^{1}>{\widehat{\mathbb{H}}}_{p}^{2}$$
5. When $${\overline{\overline{\mathrm{^\circ{\rm F} }}}}_{{\text{AV}}}\left({\widehat{\mathbb{H}}}_{p}^{1}\right) <{\overline{\overline{\mathrm{^\circ{\rm F} }}}}_{{\text{AV}}}\left({\widehat{\mathbb{H}}}_{p}^{2}\right),$$ thus $${\widehat{\mathbb{H}}}_{p}^{1}<{\widehat{\mathbb{H}}}_{p}^{2}$$
6. When $${\overline{\overline{\mathrm{^\circ{\rm F} }}}}_{{\text{AV}}}\left({\widehat{\mathbb{H}}}_{p}^{1}\right)$$ $$={\overline{\overline{\mathrm{^\circ{\rm F} }}}}_{{\text{AV}}}\left({\widehat{\mathbb{H}}}_{p}^{2}\right),$$ thus $${\widehat{\mathbb{H}}}_{p}^{1}={\widehat{\mathbb{H}}}_{p}^{2}$$

## Aczel–Alsina power aggregation operators for CTSHFSs

This section introduces the theory of CTSHFAAWPA operator, CTSHFAAOWPA operator, CTSHFAAWPG operator, and CTSHFAAOWPG operator. Further, we also investigate the idea of idempotency, monotonicity, and boundedness based on the weight vectors, such as ${\widehat{\Xi }}_{s}^{j}\in \left[{\rm O},1\right],$ where ${\sum }_{j=1}^{n}{\widehat{\Xi }}_{s}^{j}=1$.

### Definition 10

The mathematical information

$$CTSHFAAWPA\left({\widehat{\mathbb{H}}}_{p}^{1},{\widehat{\mathbb{H}}}_{p}^{2},\dots ,{\widehat{\mathbb{H}}}_{p}^{n}\right)={\widehat{\Xi }}_{s}^{1}{\widehat{\mathbb{H}}}_{p}^{1}\oplus{\widehat{\Xi }}_{s}^{2}{\widehat{\mathbb{H}}}_{p}^{2}\oplus\dots \oplus{\widehat{\Xi }}_{s}{\widehat{\mathbb{H}}}_{p}^{1}={\oplus}_{j=1}^{n}\left({\widehat{\Xi }}_{s}^{j}{\widehat{\mathbb{H}}}_{p}^{j}\right)$$

Represents the CTSHFAAWPA operator, such as

$${\widehat{\Xi }}_{s}^{i}=\frac{1+T\left({\mathbb{A}}_{i}\right)}{\sum_{i=1}^{n}\left(1+T\left({\mathbb{A}}_{i}\right)\right)},i=\mathrm{1,2},\dots ,n$$

Shown the power operator which is used as a weight vector.

### Theorem 1

Information in Def. (10), we derive that the aggregated information of the above theory is again a CTSHFN, such as

$$CTSHFAAWPA\left({\widehat{\mathbb{H}}}_{p}^{1},{\widehat{\mathbb{H}}}_{p}^{2},\dots ,{\widehat{\mathbb{H}}}_{p}^{n}\right)=\coprod_{\left(\begin{array}{c}{\overline{\overline{\mathbb{T}}}}_{{\widehat{\mathbb{H}}}_{r}}^{j},{\overline{\overline{\mathbb{T}}}}_{{\widehat{\mathbb{H}}}_{i}}^{j}\in {\overline{\overline{\mathbb{T}}}}_{{\widehat{\mathbb{H}}}_{\begin{array}{c}p\end{array}}}\\ {\overline{\overline{\mathbb{V}}}}_{{\widehat{\mathbb{H}}}_{r}}^{j},{\overline{\overline{\mathbb{V}}}}_{{\widehat{\mathbb{H}}}_{a}}^{j}\in {\overline{\overline{\mathbb{V}}}}_{{\widehat{\mathbb{H}}}_{\begin{array}{c}p\end{array}}}\\ {\overline{\overline{\mathbb{U}}}}_{{\widehat{\mathbb{H}}}_{r}}^{j},{\overline{\overline{\mathbb{U}}}}_{{\widehat{\mathbb{H}}}_{i}}^{j}\in {\overline{\overline{\mathbb{U}}}}_{{\widehat{\mathbb{H}}}_{\begin{array}{c}p\end{array}}}\end{array}\right)}\left(\begin{array}{c}\genfrac{}{}{0pt}{}{\begin{array}{c}{\left(1-{\mathcal{e}}^{-{\left({\sum }_{j=1}^{n}{{\widehat{\Xi }}_{s}^{j}\left(-{\text{ln}}\left(1-{\left({\overline{\overline{\mathbb{T}}}}_{{\widehat{\mathbb{H}}}_{r}}^{j}\right)}^{\pi }\right)\right)}^{\varnothing } \right)}^{\frac{1}{\varnothing }}}\right)}^{\frac{1}{\pi }}{\mathcal{e}}^{i2\Pi {\left(1-{\mathcal{e}}^{-{\left({\sum }_{j=1}^{n}{{\widehat{\Xi }}_{s}^{j}\left(-{\text{ln}}\left(1-{\left({\overline{\overline{\mathbb{T}}}}_{{\widehat{\mathbb{H}}}_{i}}^{j}\right)}^{\pi }\right)\right)}^{\varnothing } \right)}^{\frac{1}{\varnothing }}}\right)}^{\frac{1}{\pi }}},\\ {\left({\mathcal{e}}^{-{\left({\sum }_{j=1}^{n}{{\widehat{\Xi }}_{s}^{j}\left(-{\text{ln}}\left(1-{\left({\overline{\overline{\mathbb{V}}}}_{{\widehat{\mathbb{H}}}_{r}}^{j}\right)}^{\pi }\right)\right)}^{\varnothing } \right)}^{\frac{1}{\varnothing }}}\right)}^{\frac{1}{\pi }} {\mathcal{e}}^{i2\Pi {\left({\mathcal{e}}^{-{\left({\sum }_{j=1}^{n}{{\widehat{\Xi }}_{s}^{j}\left(-{\text{ln}}\left(1-{\left({\overline{\overline{\mathbb{V}}}}_{{\widehat{\mathbb{H}}}_{a}}^{j}\right)}^{\pi }\right)\right)}^{\varnothing } \right)}^{\frac{1}{\varnothing }}}\right)}^{\frac{1}{\pi }}},\end{array}}{{\left({\mathcal{e}}^{-{\left({\sum }_{j=1}^{n}{{\widehat{\Xi }}_{s}^{j}\left(-{\text{ln}}\left(1-{\left({\overline{\overline{\mathbb{U}}}}_{{\widehat{\mathbb{H}}}_{r}}^{j}\right)}^{\pi }\right)\right)}^{\varnothing } \right)}^{\frac{1}{\varnothing }}}\right)}^{\frac{1}{\pi }} {\mathcal{e}}^{i2\Pi {\left({\mathcal{e}}^{-{\left({\sum }_{j=1}^{n}{{\widehat{\Xi }}_{s}^{j}\left(-{\text{ln}}\left(1-{\left({\overline{\overline{\mathbb{U}}}}_{{\widehat{\mathbb{H}}}_{i}}^{j}\right)}^{\pi }\right)\right)}^{\varnothing } \right)}^{\frac{1}{\varnothing }}}\right)}^{\frac{1}{\pi }}}}\end{array}\right)$$

### Proof

For $n=2$ we prove that the above information, we have

$${\widehat{\Xi }}_{s}^{1}{\widehat{\mathbb{H}}}_{p}^{1}=\coprod_{\left(\begin{array}{c}{\overline{\overline{\mathbb{T}}}}_{{\widehat{\mathbb{H}}}_{r}}^{1},{\overline{\overline{\mathbb{T}}}}_{{\widehat{\mathbb{H}}}_{i}}^{1}\in {\overline{\overline{\mathbb{T}}}}_{{\widehat{\mathbb{H}}}_{\begin{array}{c}p\end{array}}}\\ {\overline{\overline{\mathbb{V}}}}_{{\widehat{\mathbb{H}}}_{r}}^{1},{\overline{\overline{\mathbb{V}}}}_{{\widehat{\mathbb{H}}}_{a}}^{1}\in {\overline{\overline{\mathbb{V}}}}_{{\widehat{\mathbb{H}}}_{\begin{array}{c}p\end{array}}}\\ {\overline{\overline{\mathbb{U}}}}_{{\widehat{\mathbb{H}}}_{r}}^{1},{\overline{\overline{\mathbb{U}}}}_{{\widehat{\mathbb{H}}}_{i}}^{1}\in {\overline{\overline{\mathbb{U}}}}_{{\widehat{\mathbb{H}}}_{\begin{array}{c}p\end{array}}}\end{array}\right)}\left(\begin{array}{c}\genfrac{}{}{0pt}{}{\begin{array}{c}{\left(1-{\mathcal{e}}^{-{\left({\widehat{\Xi }}_{s}^{1} {\left(-{\text{ln}}\left(1-{\left({\overline{\overline{\mathbb{T}}}}_{{\widehat{\mathbb{H}}}_{r}}^{1}\right)}^{\pi }\right)\right)}^{\varnothing }\right)}^{\frac{1}{\varnothing }}}\right)}^{\frac{1}{\pi }}{\mathcal{e}}^{i2\Pi {\left(1-{\mathcal{e}}^{-{\left({\widehat{\Xi }}_{s}^{1}{\left(-{\text{ln}}\left(1-{\left({\overline{\overline{\mathbb{T}}}}_{{\widehat{\mathbb{H}}}_{i}}^{1}\right)}^{\pi }\right)\right)}^{\varnothing }\right)}^{\frac{1}{\varnothing }}}\right)}^{\frac{1}{\pi }}},\\ {\left({\mathcal{e}}^{-{\left({\widehat{\Xi }}_{s}^{1}{\left(-{\text{ln}}\left({\left({\overline{\overline{\mathbb{V}}}}_{{\widehat{\mathbb{H}}}_{r}}^{1}\right)}^{\pi }\right)\right)}^{\varnothing }\right)}^{\frac{1}{\varnothing }}}\right)}^{\frac{1}{\pi }} {\mathcal{e}}^{i2\Pi {\left({\mathcal{e}}^{-{\left({\widehat{\Xi }}_{s}^{1}{\left(-{\text{ln}}\left({\left({\overline{\overline{\mathbb{V}}}}_{{\widehat{\mathbb{H}}}_{a}}^{1}\right)}^{\pi }\right)\right)}^{\varnothing }\right)}^{\frac{1}{\varnothing }}}\right)}^{\frac{1}{\pi }}},\end{array}}{{\left({\mathcal{e}}^{-{\left({\widehat{\Xi }}_{s}^{1}{\left(-{\text{ln}}\left({\left({\overline{\overline{\mathbb{U}}}}_{{\widehat{\mathbb{H}}}_{r}}^{1}\right)}^{\pi }\right)\right)}^{\varnothing }\right)}^{\frac{1}{\varnothing }}}\right)}^{\frac{1}{\pi }} {\mathcal{e}}^{i2\Pi {\left({\mathcal{e}}^{-{\left({\widehat{\Xi }}_{s}^{1}{\left(-{\text{ln}}\left({\left({\overline{\overline{\mathbb{U}}}}_{{\widehat{\mathbb{H}}}_{i}}^{1}\right)}^{\pi }\right)\right)}^{\varnothing }\right)}^{\frac{1}{\varnothing }}}\right)}^{\frac{1}{\pi }}}}\end{array}\right)$$

$${\widehat{\Xi }}_{s}^{2}{\widehat{\mathbb{H}}}_{p}^{2}=\coprod_{\left(\begin{array}{c}{\overline{\overline{\mathbb{T}}}}_{{\widehat{\mathbb{H}}}_{r}}^{2},{\overline{\overline{\mathbb{T}}}}_{{\widehat{\mathbb{H}}}_{i}}^{2}\in {\overline{\overline{\mathbb{T}}}}_{{\widehat{\mathbb{H}}}_{\begin{array}{c}p\end{array}}}\\ {\overline{\overline{\mathbb{V}}}}_{{\widehat{\mathbb{H}}}_{r}}^{2},{\overline{\overline{\mathbb{V}}}}_{{\widehat{\mathbb{H}}}_{a}}^{2}\in {\overline{\overline{\mathbb{V}}}}_{{\widehat{\mathbb{H}}}_{\begin{array}{c}p\end{array}}}\\ {\overline{\overline{\mathbb{U}}}}_{{\widehat{\mathbb{H}}}_{r}}^{2},{\overline{\overline{\mathbb{U}}}}_{{\widehat{\mathbb{H}}}_{i}}^{2}\in {\overline{\overline{\mathbb{U}}}}_{{\widehat{\mathbb{H}}}_{\begin{array}{c}p\end{array}}}\end{array}\right)}\left(\begin{array}{c}\genfrac{}{}{0pt}{}{\begin{array}{c}{\left(1-{\mathcal{e}}^{-{\left({\widehat{\Xi }}_{s}^{2} {\left(-{\text{ln}}\left(1-{\left({\overline{\overline{\mathbb{T}}}}_{{\widehat{\mathbb{H}}}_{r}}^{2}\right)}^{\pi }\right)\right)}^{\varnothing }\right)}^{\frac{1}{\varnothing }}}\right)}^{\frac{1}{\pi }}{\mathcal{e}}^{i2\Pi {\left(1-{\mathcal{e}}^{-{\left({\widehat{\Xi }}_{s}^{2}{\left(-{\text{ln}}\left(1-{\left({\overline{\overline{\mathbb{T}}}}_{{\widehat{\mathbb{H}}}_{i}}^{2}\right)}^{\pi }\right)\right)}^{\varnothing }\right)}^{\frac{1}{\varnothing }}}\right)}^{\frac{1}{\pi }}},\\ {\left({\mathcal{e}}^{-{\left({\widehat{\Xi }}_{s}^{2}{\left(-{\text{ln}}\left({\left({\overline{\overline{\mathbb{V}}}}_{{\widehat{\mathbb{H}}}_{r}}^{2}\right)}^{\pi }\right)\right)}^{\varnothing }\right)}^{\frac{1}{\varnothing }}}\right)}^{\frac{1}{\pi }} {\mathcal{e}}^{i2\Pi {\left({\mathcal{e}}^{-{\left({\widehat{\Xi }}_{s}^{2}{\left(-{\text{ln}}\left({\left({\overline{\overline{\mathbb{V}}}}_{{\widehat{\mathbb{H}}}_{a}}^{2}\right)}^{\pi }\right)\right)}^{\varnothing }\right)}^{\frac{1}{\varnothing }}}\right)}^{\frac{1}{\pi }}},\end{array}}{{\left({\mathcal{e}}^{-{\left({\widehat{\Xi }}_{s}^{2}{\left(-{\text{ln}}\left({\left({\overline{\overline{\mathbb{U}}}}_{{\widehat{\mathbb{H}}}_{r}}^{2}\right)}^{\pi }\right)\right)}^{\varnothing }\right)}^{\frac{1}{\varnothing }}}\right)}^{\frac{1}{\pi }} {\mathcal{e}}^{i2\Pi {\left({\mathcal{e}}^{-{\left({\widehat{\Xi }}_{s}^{2}{\left(-{\text{ln}}\left({\left({\overline{\overline{\mathbb{U}}}}_{{\widehat{\mathbb{H}}}_{i}}^{2}\right)}^{\pi }\right)\right)}^{\varnothing }\right)}^{\frac{1}{\varnothing }}}\right)}^{\frac{1}{\pi }}}}\end{array}\right)$$

$$CTSHFAAWPA\left({\widehat{\mathbb{H}}}_{p}^{1},{\widehat{\mathbb{H}}}_{p}^{2}\right)={\widehat{\Xi }}_{s}^{1}{\widehat{\mathbb{H}}}_{p}^{1}\oplus {\widehat{\Xi }}_{s}^{2}{\widehat{\mathbb{H}}}_{p}^{2}$$

$$=\coprod_{\left(\begin{array}{c}{\overline{\overline{\mathbb{T}}}}_{{\widehat{\mathbb{H}}}_{r}}^{1},{\overline{\overline{\mathbb{T}}}}_{{\widehat{\mathbb{H}}}_{i}}^{1}\in {\overline{\overline{\mathbb{T}}}}_{{\widehat{\mathbb{H}}}_{\begin{array}{c}p\end{array}}}\\ {\overline{\overline{\mathbb{V}}}}_{{\widehat{\mathbb{H}}}_{r}}^{1},{\overline{\overline{\mathbb{V}}}}_{{\widehat{\mathbb{H}}}_{a}}^{1}\in {\overline{\overline{\mathbb{V}}}}_{{\widehat{\mathbb{H}}}_{\begin{array}{c}p\end{array}}}\\ {\overline{\overline{\mathbb{U}}}}_{{\widehat{\mathbb{H}}}_{r}}^{1},{\overline{\overline{\mathbb{U}}}}_{{\widehat{\mathbb{H}}}_{i}}^{1}\in {\overline{\overline{\mathbb{U}}}}_{{\widehat{\mathbb{H}}}_{\begin{array}{c}p\end{array}}}\end{array}\right)}\left(\begin{array}{c}\genfrac{}{}{0pt}{}{\begin{array}{c}{\left(1-{\mathcal{e}}^{-{\left({\widehat{\Xi }}_{s}^{1} {\left(-{\text{ln}}\left(1-{\left({\overline{\overline{\mathbb{T}}}}_{{\widehat{\mathbb{H}}}_{r}}^{1}\right)}^{\pi }\right)\right)}^{\varnothing }\right)}^{\frac{1}{\varnothing }}}\right)}^{\frac{1}{\pi }}{\mathcal{e}}^{i2\Pi {\left(1-{\mathcal{e}}^{-{\left({\widehat{\Xi }}_{s}^{1}{\left(-{\text{ln}}\left(1-{\left({\overline{\overline{\mathbb{T}}}}_{{\widehat{\mathbb{H}}}_{i}}^{1}\right)}^{\pi }\right)\right)}^{\varnothing }\right)}^{\frac{1}{\varnothing }}}\right)}^{\frac{1}{\pi }}},\\ {\left({\mathcal{e}}^{-{\left({\widehat{\Xi }}_{s}^{1}{\left(-{\text{ln}}\left({\left({\overline{\overline{\mathbb{V}}}}_{{\widehat{\mathbb{H}}}_{r}}^{1}\right)}^{\pi }\right)\right)}^{\varnothing }\right)}^{\frac{1}{\varnothing }}}\right)}^{\frac{1}{\pi }} {\mathcal{e}}^{i2\Pi {\left({\mathcal{e}}^{-{\left({\widehat{\Xi }}_{s}^{1}{\left(-{\text{ln}}\left({\left({\overline{\overline{\mathbb{V}}}}_{{\widehat{\mathbb{H}}}_{a}}^{1}\right)}^{\pi }\right)\right)}^{\varnothing }\right)}^{\frac{1}{\varnothing }}}\right)}^{\frac{1}{\pi }}},\end{array}}{{\left({\mathcal{e}}^{-{\left({\widehat{\Xi }}_{s}^{1}{\left(-{\text{ln}}\left({\left({\overline{\overline{\mathbb{U}}}}_{{\widehat{\mathbb{H}}}_{r}}^{1}\right)}^{\pi }\right)\right)}^{\varnothing }\right)}^{\frac{1}{\varnothing }}}\right)}^{\frac{1}{\pi }} {\mathcal{e}}^{i2\Pi {\left({\mathcal{e}}^{-{\left({\widehat{\Xi }}_{s}^{1}{\left(-{\text{ln}}\left({\left({\overline{\overline{\mathbb{U}}}}_{{\widehat{\mathbb{H}}}_{i}}^{1}\right)}^{\pi }\right)\right)}^{\varnothing }\right)}^{\frac{1}{\varnothing }}}\right)}^{\frac{1}{\pi }}}}\end{array}\right)$$

$$\oplus\coprod_{\left(\begin{array}{c}{\overline{\overline{\mathbb{T}}}}_{{\widehat{\mathbb{H}}}_{r}}^{2},{\overline{\overline{\mathbb{T}}}}_{{\widehat{\mathbb{H}}}_{i}}^{2}\in {\overline{\overline{\mathbb{T}}}}_{{\widehat{\mathbb{H}}}_{\begin{array}{c}p\end{array}}}\\ {\overline{\overline{\mathbb{V}}}}_{{\widehat{\mathbb{H}}}_{r}}^{2},{\overline{\overline{\mathbb{V}}}}_{{\widehat{\mathbb{H}}}_{a}}^{2}\in {\overline{\overline{\mathbb{V}}}}_{{\widehat{\mathbb{H}}}_{\begin{array}{c}p\end{array}}}\\ {\overline{\overline{\mathbb{U}}}}_{{\widehat{\mathbb{H}}}_{r}}^{2},{\overline{\overline{\mathbb{U}}}}_{{\widehat{\mathbb{H}}}_{i}}^{2}\in {\overline{\overline{\mathbb{U}}}}_{{\widehat{\mathbb{H}}}_{\begin{array}{c}p\end{array}}}\end{array}\right)}\left(\begin{array}{c}\genfrac{}{}{0pt}{}{\begin{array}{c}{\left(1-{\mathcal{e}}^{-{\left({\widehat{\Xi }}_{s}^{2} {\left(-{\text{ln}}\left(1-{\left({\overline{\overline{\mathbb{T}}}}_{{\widehat{\mathbb{H}}}_{r}}^{2}\right)}^{\pi }\right)\right)}^{\varnothing }\right)}^{\frac{1}{\varnothing }}}\right)}^{\frac{1}{\pi }}{\mathcal{e}}^{i2\Pi {\left(1-{\mathcal{e}}^{-{\left({\widehat{\Xi }}_{s}^{2}{\left(-{\text{ln}}\left(1-{\left({\overline{\overline{\mathbb{T}}}}_{{\widehat{\mathbb{H}}}_{i}}^{2}\right)}^{\pi }\right)\right)}^{\varnothing }\right)}^{\frac{1}{\varnothing }}}\right)}^{\frac{1}{\pi }}},\\ {\left({\mathcal{e}}^{-{\left({\widehat{\Xi }}_{s}^{2}{\left(-{\text{ln}}\left({\left({\overline{\overline{\mathbb{V}}}}_{{\widehat{\mathbb{H}}}_{r}}^{2}\right)}^{\pi }\right)\right)}^{\varnothing }\right)}^{\frac{1}{\varnothing }}}\right)}^{\frac{1}{\pi }} {\mathcal{e}}^{i2\Pi {\left({\mathcal{e}}^{-{\left({\widehat{\Xi }}_{s}^{2}{\left(-{\text{ln}}\left({\left({\overline{\overline{\mathbb{V}}}}_{{\widehat{\mathbb{H}}}_{a}}^{2}\right)}^{\pi }\right)\right)}^{\varnothing }\right)}^{\frac{1}{\varnothing }}}\right)}^{\frac{1}{\pi }}},\end{array}}{{\left({\mathcal{e}}^{-{\left({\widehat{\Xi }}_{s}^{2}{\left(-{\text{ln}}\left({\left({\overline{\overline{\mathbb{U}}}}_{{\widehat{\mathbb{H}}}_{r}}^{2}\right)}^{\pi }\right)\right)}^{\varnothing }\right)}^{\frac{1}{\varnothing }}}\right)}^{\frac{1}{\pi }} {\mathcal{e}}^{i2\Pi {\left({\mathcal{e}}^{-{\left({\widehat{\Xi }}_{s}^{2}{\left(-{\text{ln}}\left({\left({\overline{\overline{\mathbb{U}}}}_{{\widehat{\mathbb{H}}}_{i}}^{2}\right)}^{\pi }\right)\right)}^{\varnothing }\right)}^{\frac{1}{\varnothing }}}\right)}^{\frac{1}{\pi }}}}\end{array}\right)$$

$$=\coprod_{\left(\begin{array}{c}{\overline{\overline{\mathbb{T}}}}_{{\widehat{\mathbb{H}}}_{r}}^{j},{\overline{\overline{\mathbb{T}}}}_{{\widehat{\mathbb{H}}}_{i}}^{j}\in {\overline{\overline{\mathbb{T}}}}_{{\widehat{\mathbb{H}}}_{\begin{array}{c}p\end{array}}}\\ {\overline{\overline{\mathbb{V}}}}_{{\widehat{\mathbb{H}}}_{r}}^{j},{\overline{\overline{\mathbb{V}}}}_{{\widehat{\mathbb{H}}}_{a}}^{j}\in {\overline{\overline{\mathbb{V}}}}_{{\widehat{\mathbb{H}}}_{\begin{array}{c}p\end{array}}}\\ {\overline{\overline{\mathbb{U}}}}_{{\widehat{\mathbb{H}}}_{r}}^{j},{\overline{\overline{\mathbb{U}}}}_{{\widehat{\mathbb{H}}}_{i}}^{j}\in {\overline{\overline{\mathbb{U}}}}_{{\widehat{\mathbb{H}}}_{\begin{array}{c}p\end{array}}}\end{array}\right)}\left(\begin{array}{c}\genfrac{}{}{0pt}{}{\begin{array}{c}{\left(1-{\mathcal{e}}^{-{\left({\sum }_{j=1}^{2}{{\widehat{\Xi }}_{s}^{j}\left(-{\text{ln}}\left(1-{\left({\overline{\overline{\mathbb{T}}}}_{{\widehat{\mathbb{H}}}_{r}}^{j}\right)}^{\pi }\right)\right)}^{\varnothing } \right)}^{\frac{1}{\varnothing }}}\right)}^{\frac{1}{\pi }}{\mathcal{e}}^{i2\Pi {\left(1-{\mathcal{e}}^{-{\left({\sum }_{j=1}^{2}{{\widehat{\Xi }}_{s}^{j}\left(-{\text{ln}}\left(1-{\left({\overline{\overline{\mathbb{T}}}}_{{\widehat{\mathbb{H}}}_{i}}^{j}\right)}^{\pi }\right)\right)}^{\varnothing } \right)}^{\frac{1}{\varnothing }}}\right)}^{\frac{1}{\pi }}},\\ {\left({\mathcal{e}}^{-{\left({\sum }_{j=1}^{2}{{\widehat{\Xi }}_{s}^{j}\left(-{\text{ln}}\left(1-{\left({\overline{\overline{\mathbb{V}}}}_{{\widehat{\mathbb{H}}}_{r}}^{j}\right)}^{\pi }\right)\right)}^{\varnothing } \right)}^{\frac{1}{\varnothing }}}\right)}^{\frac{1}{\pi }} {\mathcal{e}}^{i2\Pi {\left({\mathcal{e}}^{-{\left({\sum }_{j=1}^{2}{{\widehat{\Xi }}_{s}^{j}\left(-{\text{ln}}\left(1-{\left({\overline{\overline{\mathbb{V}}}}_{{\widehat{\mathbb{H}}}_{a}}^{j}\right)}^{\pi }\right)\right)}^{\varnothing } \right)}^{\frac{1}{\varnothing }}}\right)}^{\frac{1}{\pi }}},\end{array}}{{\left({\mathcal{e}}^{-{\left({\sum }_{j=1}^{2}{{\widehat{\Xi }}_{s}^{j}\left(-{\text{ln}}\left(1-{\left({\overline{\overline{\mathbb{U}}}}_{{\widehat{\mathbb{H}}}_{r}}^{j}\right)}^{\pi }\right)\right)}^{\varnothing } \right)}^{\frac{1}{\varnothing }}}\right)}^{\frac{1}{\pi }} {\mathcal{e}}^{i2\Pi {\left({\mathcal{e}}^{-{\left({\sum }_{j=1}^{2}{{\widehat{\Xi }}_{s}^{j}\left(-{\text{ln}}\left(1-{\left({\overline{\overline{\mathbb{U}}}}_{{\widehat{\mathbb{H}}}_{i}}^{j}\right)}^{\pi }\right)\right)}^{\varnothing } \right)}^{\frac{1}{\varnothing }}}\right)}^{\frac{1}{\pi }}}}\end{array}\right)$$

□

Our problem is correct for n = 2, Further, we take it for $n=k$, such as

$$CTSHFAAWPA\left({\widehat{\mathbb{H}}}_{p}^{1},{\widehat{\mathbb{H}}}_{p}^{2},\dots ,{\widehat{\mathbb{H}}}_{p}^{n}\right)=\coprod_{\left(\begin{array}{c}{\overline{\overline{\mathbb{T}}}}_{{\widehat{\mathbb{H}}}_{r}}^{j},{\overline{\overline{\mathbb{T}}}}_{{\widehat{\mathbb{H}}}_{i}}^{j}\in {\overline{\overline{\mathbb{T}}}}_{{\widehat{\mathbb{H}}}_{\begin{array}{c}p\end{array}}}\\ {\overline{\overline{\mathbb{V}}}}_{{\widehat{\mathbb{H}}}_{r}}^{j},{\overline{\overline{\mathbb{V}}}}_{{\widehat{\mathbb{H}}}_{a}}^{j}\in {\overline{\overline{\mathbb{V}}}}_{{\widehat{\mathbb{H}}}_{\begin{array}{c}p\end{array}}}\\ {\overline{\overline{\mathbb{U}}}}_{{\widehat{\mathbb{H}}}_{r}}^{j},{\overline{\overline{\mathbb{U}}}}_{{\widehat{\mathbb{H}}}_{i}}^{j}\in {\overline{\overline{\mathbb{U}}}}_{{\widehat{\mathbb{H}}}_{\begin{array}{c}p\end{array}}}\end{array}\right)}\left(\begin{array}{c}\genfrac{}{}{0pt}{}{\begin{array}{c}{\left(1-{\mathcal{e}}^{-{\left({\sum }_{j=1}^{k}{{\widehat{\Xi }}_{s}^{j}\left(-{\text{ln}}\left(1-{\left({\overline{\overline{\mathbb{T}}}}_{{\widehat{\mathbb{H}}}_{r}}^{j}\right)}^{\pi }\right)\right)}^{\varnothing } \right)}^{\frac{1}{\varnothing }}}\right)}^{\frac{1}{\pi }}{\mathcal{e}}^{i2\Pi {\left(1-{\mathcal{e}}^{-{\left({\sum }_{j=1}^{k}{{\widehat{\Xi }}_{s}^{j}\left(-{\text{ln}}\left(1-{\left({\overline{\overline{\mathbb{T}}}}_{{\widehat{\mathbb{H}}}_{i}}^{j}\right)}^{\pi }\right)\right)}^{\varnothing } \right)}^{\frac{1}{\varnothing }}}\right)}^{\frac{1}{\pi }}},\\ {\left({\mathcal{e}}^{-{\left({\sum }_{j=1}^{k}{{\widehat{\Xi }}_{s}^{j}\left(-{\text{ln}}\left(1-{\left({\overline{\overline{\mathbb{V}}}}_{{\widehat{\mathbb{H}}}_{r}}^{j}\right)}^{\pi }\right)\right)}^{\varnothing } \right)}^{\frac{1}{\varnothing }}}\right)}^{\frac{1}{\pi }} {\mathcal{e}}^{i2\Pi {\left({\mathcal{e}}^{-{\left({\sum }_{j=1}^{k}{{\widehat{\Xi }}_{s}^{j}\left(-{\text{ln}}\left(1-{\left({\overline{\overline{\mathbb{V}}}}_{{\widehat{\mathbb{H}}}_{a}}^{j}\right)}^{\pi }\right)\right)}^{\varnothing } \right)}^{\frac{1}{\varnothing }}}\right)}^{\frac{1}{\pi }}},\end{array}}{{\left({\mathcal{e}}^{-{\left({\sum }_{j=1}^{k}{{\widehat{\Xi }}_{s}^{j}\left(-{\text{ln}}\left(1-{\left({\overline{\overline{\mathbb{U}}}}_{{\widehat{\mathbb{H}}}_{r}}^{j}\right)}^{\pi }\right)\right)}^{\varnothing } \right)}^{\frac{1}{\varnothing }}}\right)}^{\frac{1}{\pi }} {\mathcal{e}}^{i2\Pi {\left({\mathcal{e}}^{-{\left({\sum }_{j=1}^{k}{{\widehat{\Xi }}_{s}^{j}\left(-{\text{ln}}\left(1-{\left({\overline{\overline{\mathbb{U}}}}_{{\widehat{\mathbb{H}}}_{i}}^{j}\right)}^{\pi }\right)\right)}^{\varnothing } \right)}^{\frac{1}{\varnothing }}}\right)}^{\frac{1}{\pi }}}}\end{array}\right)$$

Then, we compute it for n = k + 1, such as

$$CTSHFAAWA\left({\widehat{\mathbb{H}}}_{p}^{1},{\widehat{\mathbb{H}}}_{p}^{2},\dots ,{\widehat{\mathbb{H}}}_{p}^{K+1}\right)={\widehat{\Xi }}_{s}^{1}{\widehat{\mathbb{H}}}_{p}^{1}\oplus{\widehat{\Xi }}_{s}^{2}{\widehat{\mathbb{H}}}_{p}^{2}\oplus\dots \oplus{{\widehat{\Xi }}_{s}^{K}\widehat{\mathbb{H}}}_{p}^{K}\oplus{{\widehat{\Xi }}_{s}^{K+1}\widehat{\mathbb{H}}}_{p}^{K+1}={\oplus}_{j=1}^{n}\left({\widehat{\Xi }}_{s}^{j}{\widehat{\mathbb{H}}}_{p}^{j}\right)\oplus {{\widehat{\Xi }}_{s}^{K+1}\widehat{\mathbb{H}}}_{p}^{K+1}$$

$$=\coprod_{\left(\begin{array}{c}{\overline{\overline{\mathbb{T}}}}_{{\widehat{\mathbb{H}}}_{r}}^{j},{\overline{\overline{\mathbb{T}}}}_{{\widehat{\mathbb{H}}}_{i}}^{j}\in {\overline{\overline{\mathbb{T}}}}_{{\widehat{\mathbb{H}}}_{\begin{array}{c}p\end{array}}}\\ {\overline{\overline{\mathbb{V}}}}_{{\widehat{\mathbb{H}}}_{r}}^{j},{\overline{\overline{\mathbb{V}}}}_{{\widehat{\mathbb{H}}}_{a}}^{j}\in {\overline{\overline{\mathbb{V}}}}_{{\widehat{\mathbb{H}}}_{\begin{array}{c}p\end{array}}}\\ {\overline{\overline{\mathbb{U}}}}_{{\widehat{\mathbb{H}}}_{r}}^{j},{\overline{\overline{\mathbb{U}}}}_{{\widehat{\mathbb{H}}}_{i}}^{j}\in {\overline{\overline{\mathbb{U}}}}_{{\widehat{\mathbb{H}}}_{\begin{array}{c}p\end{array}}}\end{array}\right)}\begin{array}{c}\left(\begin{array}{c}\genfrac{}{}{0pt}{}{\begin{array}{c}{\left(1-{\mathcal{e}}^{-{\left({\sum }_{j=1}^{k}{{\widehat{\Xi }}_{s}^{j}\left(-{\text{ln}}\left(1-{\left({\overline{\overline{\mathbb{T}}}}_{{\widehat{\mathbb{H}}}_{r}}^{j}\right)}^{\pi }\right)\right)}^{\varnothing } \right)}^{\frac{1}{\varnothing }}}\right)}^{\frac{1}{\pi }}{\mathcal{e}}^{i2\Pi {\left(1-{\mathcal{e}}^{-{\left({\sum }_{j=1}^{k}{{\widehat{\Xi }}_{s}^{j}\left(-{\text{ln}}\left(1-{\left({\overline{\overline{\mathbb{T}}}}_{{\widehat{\mathbb{H}}}_{i}}^{j}\right)}^{\pi }\right)\right)}^{\varnothing } \right)}^{\frac{1}{\varnothing }}}\right)}^{\frac{1}{\pi }}},\\ {\left({\mathcal{e}}^{-{\left({\sum }_{j=1}^{k}{{\widehat{\Xi }}_{s}^{j}\left(-{\text{ln}}\left(1-{\left({\overline{\overline{\mathbb{V}}}}_{{\widehat{\mathbb{H}}}_{r}}^{j}\right)}^{\pi }\right)\right)}^{\varnothing } \right)}^{\frac{1}{\varnothing }}}\right)}^{\frac{1}{\pi }} {\mathcal{e}}^{i2\Pi {\left({\mathcal{e}}^{-{\left({\sum }_{j=1}^{k}{{\widehat{\Xi }}_{s}^{j}\left(-{\text{ln}}\left(1-{\left({\overline{\overline{\mathbb{V}}}}_{{\widehat{\mathbb{H}}}_{a}}^{j}\right)}^{\pi }\right)\right)}^{\varnothing } \right)}^{\frac{1}{\varnothing }}}\right)}^{\frac{1}{\pi }}},\end{array}}{{\left({\mathcal{e}}^{-{\left({\sum }_{j=1}^{k}{{\widehat{\Xi }}_{s}^{j}\left(-{\text{ln}}\left(1-{\left({\overline{\overline{\mathbb{U}}}}_{{\widehat{\mathbb{H}}}_{r}}^{j}\right)}^{\pi }\right)\right)}^{\varnothing } \right)}^{\frac{1}{\varnothing }}}\right)}^{\frac{1}{\pi }} {\mathcal{e}}^{i2\Pi {\left({\mathcal{e}}^{-{\left({\sum }_{j=1}^{k}{{\widehat{\Xi }}_{s}^{j}\left(-{\text{ln}}\left(1-{\left({\overline{\overline{\mathbb{U}}}}_{{\widehat{\mathbb{H}}}_{i}}^{j}\right)}^{\pi }\right)\right)}^{\varnothing } \right)}^{\frac{1}{\varnothing }}}\right)}^{\frac{1}{\pi }}}}\end{array}\right)\\ \end{array}\oplus{{\widehat{\Xi }}_{s}^{K+1}\widehat{\mathbb{H}}}_{p}^{K+1}$$

$$=\coprod_{\left(\begin{array}{c}{\overline{\overline{\mathbb{T}}}}_{{\widehat{\mathbb{H}}}_{r}}^{j},{\overline{\overline{\mathbb{T}}}}_{{\widehat{\mathbb{H}}}_{i}}^{j}\in {\overline{\overline{\mathbb{T}}}}_{{\widehat{\mathbb{H}}}_{\begin{array}{c}p\end{array}}}\\ {\overline{\overline{\mathbb{V}}}}_{{\widehat{\mathbb{H}}}_{r}}^{j},{\overline{\overline{\mathbb{V}}}}_{{\widehat{\mathbb{H}}}_{a}}^{j}\in {\overline{\overline{\mathbb{V}}}}_{{\widehat{\mathbb{H}}}_{\begin{array}{c}p\end{array}}}\\ {\overline{\overline{\mathbb{U}}}}_{{\widehat{\mathbb{H}}}_{r}}^{j},{\overline{\overline{\mathbb{U}}}}_{{\widehat{\mathbb{H}}}_{i}}^{j}\in {\overline{\overline{\mathbb{U}}}}_{{\widehat{\mathbb{H}}}_{\begin{array}{c}p\end{array}}}\end{array}\right)}\begin{array}{c}\left(\begin{array}{c}\genfrac{}{}{0pt}{}{\begin{array}{c}{\left(1-{\mathcal{e}}^{-{\left({\sum }_{j=1}^{k}{{\widehat{\Xi }}_{s}^{j}\left(-{\text{ln}}\left(1-{\left({\overline{\overline{\mathbb{T}}}}_{{\widehat{\mathbb{H}}}_{r}}^{j}\right)}^{\pi }\right)\right)}^{\varnothing } \right)}^{\frac{1}{\varnothing }}}\right)}^{\frac{1}{\pi }}{\mathcal{e}}^{i2\Pi {\left(1-{\mathcal{e}}^{-{\left({\sum }_{j=1}^{k}{{\widehat{\Xi }}_{s}^{j}\left(-{\text{ln}}\left(1-{\left({\overline{\overline{\mathbb{T}}}}_{{\widehat{\mathbb{H}}}_{i}}^{j}\right)}^{\pi }\right)\right)}^{\varnothing } \right)}^{\frac{1}{\varnothing }}}\right)}^{\frac{1}{\pi }}},\\ {\left({\mathcal{e}}^{-{\left({\sum }_{j=1}^{k}{{\widehat{\Xi }}_{s}^{j}\left(-{\text{ln}}\left(1-{\left({\overline{\overline{\mathbb{V}}}}_{{\widehat{\mathbb{H}}}_{r}}^{j}\right)}^{\pi }\right)\right)}^{\varnothing } \right)}^{\frac{1}{\varnothing }}}\right)}^{\frac{1}{\pi }} {\mathcal{e}}^{i2\Pi {\left({\mathcal{e}}^{-{\left({\sum }_{j=1}^{k}{{\widehat{\Xi }}_{s}^{j}\left(-{\text{ln}}\left(1-{\left({\overline{\overline{\mathbb{V}}}}_{{\widehat{\mathbb{H}}}_{a}}^{j}\right)}^{\pi }\right)\right)}^{\varnothing } \right)}^{\frac{1}{\varnothing }}}\right)}^{\frac{1}{\pi }}},\end{array}}{{\left({\mathcal{e}}^{-{\left({\sum }_{j=1}^{k}{{\widehat{\Xi }}_{s}^{j}\left(-{\text{ln}}\left(1-{\left({\overline{\overline{\mathbb{U}}}}_{{\widehat{\mathbb{H}}}_{r}}^{j}\right)}^{\pi }\right)\right)}^{\varnothing } \right)}^{\frac{1}{\varnothing }}}\right)}^{\frac{1}{\pi }} {\mathcal{e}}^{i2\Pi {\left({\mathcal{e}}^{-{\left({\sum }_{j=1}^{k}{{\widehat{\Xi }}_{s}^{j}\left(-{\text{ln}}\left(1-{\left({\overline{\overline{\mathbb{U}}}}_{{\widehat{\mathbb{H}}}_{i}}^{j}\right)}^{\pi }\right)\right)}^{\varnothing } \right)}^{\frac{1}{\varnothing }}}\right)}^{\frac{1}{\pi }}}}\end{array}\right)\\ \end{array}$$

$$\oplus\coprod_{\left(\begin{array}{c}{\overline{\overline{\mathbb{T}}}}_{{\widehat{\mathbb{H}}}_{r}}^{K+1},{\overline{\overline{\mathbb{T}}}}_{{\widehat{\mathbb{H}}}_{i}}^{K+1}\in {\overline{\overline{\mathbb{T}}}}_{{\widehat{\mathbb{H}}}_{\begin{array}{c}p\end{array}}}\\ {\overline{\overline{\mathbb{V}}}}_{{\widehat{\mathbb{H}}}_{r}}^{K+1},{\overline{\overline{\mathbb{V}}}}_{{\widehat{\mathbb{H}}}_{a}}^{K+1}\in {\overline{\overline{\mathbb{V}}}}_{{\widehat{\mathbb{H}}}_{\begin{array}{c}p\end{array}}}\\ {\overline{\overline{\mathbb{U}}}}_{{\widehat{\mathbb{H}}}_{r}}^{K+1},{\overline{\overline{\mathbb{U}}}}_{{\widehat{\mathbb{H}}}_{i}}^{K+1}\in {\overline{\overline{\mathbb{U}}}}_{{\widehat{\mathbb{H}}}_{\begin{array}{c}p\end{array}}}\end{array}\right)}\left(\begin{array}{c}\genfrac{}{}{0pt}{}{\begin{array}{c}{\left(1-{\mathcal{e}}^{-{\left({\widehat{\Xi }}_{s}^{K+1} {\left(-{\text{ln}}\left(1-{\left({\overline{\overline{\mathbb{T}}}}_{{\widehat{\mathbb{H}}}_{r}}^{K+1}\right)}^{\pi }\right)\right)}^{\varnothing }\right)}^{\frac{1}{\varnothing }}}\right)}^{\frac{1}{\pi }}{\mathcal{e}}^{i2\Pi {\left(1-{\mathcal{e}}^{-{\left({\widehat{\Xi }}_{s}^{K+1}{\left(-{\text{ln}}\left(1-{\left({\overline{\overline{\mathbb{T}}}}_{{\widehat{\mathbb{H}}}_{i}}^{K+1}\right)}^{\pi }\right)\right)}^{\varnothing }\right)}^{\frac{1}{\varnothing }}}\right)}^{\frac{1}{\pi }}},\\ {\left({\mathcal{e}}^{-{\left({\widehat{\Xi }}_{s}^{K+1}{\left(-{\text{ln}}\left({\left({\overline{\overline{\mathbb{V}}}}_{{\widehat{\mathbb{H}}}_{r}}^{K+1}\right)}^{\pi }\right)\right)}^{\varnothing }\right)}^{\frac{1}{\varnothing }}}\right)}^{\frac{1}{\pi }} {\mathcal{e}}^{i2\Pi {\left({\mathcal{e}}^{-{\left({\widehat{\Xi }}_{s}^{K+1}{\left(-{\text{ln}}\left({\left({\overline{\overline{\mathbb{V}}}}_{{\widehat{\mathbb{H}}}_{a}}^{K+1}\right)}^{\pi }\right)\right)}^{\varnothing }\right)}^{\frac{1}{\varnothing }}}\right)}^{\frac{1}{\pi }}},\end{array}}{{\left({\mathcal{e}}^{-{\left({\widehat{\Xi }}_{s}^{K+1}{\left(-{\text{ln}}\left({\left({\overline{\overline{\mathbb{U}}}}_{{\widehat{\mathbb{H}}}_{r}}^{K+1}\right)}^{\pi }\right)\right)}^{\varnothing }\right)}^{\frac{1}{\varnothing }}}\right)}^{\frac{1}{\pi }} {\mathcal{e}}^{i2\Pi {\left({\mathcal{e}}^{-{\left({\widehat{\Xi }}_{s}^{K+1}{\left(-{\text{ln}}\left({\left({\overline{\overline{\mathbb{U}}}}_{{\widehat{\mathbb{H}}}_{i}}^{K+1}\right)}^{\pi }\right)\right)}^{\varnothing }\right)}^{\frac{1}{\varnothing }}}\right)}^{\frac{1}{\pi }}}}\end{array}\right)$$

$$=\coprod_{\left(\begin{array}{c}{\overline{\overline{\mathbb{T}}}}_{{\widehat{\mathbb{H}}}_{r}}^{j},{\overline{\overline{\mathbb{T}}}}_{{\widehat{\mathbb{H}}}_{i}}^{j}\in {\overline{\overline{\mathbb{T}}}}_{{\widehat{\mathbb{H}}}_{\begin{array}{c}p\end{array}}}\\ {\overline{\overline{\mathbb{V}}}}_{{\widehat{\mathbb{H}}}_{r}}^{j},{\overline{\overline{\mathbb{V}}}}_{{\widehat{\mathbb{H}}}_{a}}^{j}\in {\overline{\overline{\mathbb{V}}}}_{{\widehat{\mathbb{H}}}_{\begin{array}{c}p\end{array}}}\\ {\overline{\overline{\mathbb{U}}}}_{{\widehat{\mathbb{H}}}_{r}}^{j},{\overline{\overline{\mathbb{U}}}}_{{\widehat{\mathbb{H}}}_{i}}^{j}\in {\overline{\overline{\mathbb{U}}}}_{{\widehat{\mathbb{H}}}_{\begin{array}{c}p\end{array}}}\end{array}\right)}\begin{array}{c}\left(\begin{array}{c}\genfrac{}{}{0pt}{}{\begin{array}{c}{\left(1-{\mathcal{e}}^{-{\left({\sum }_{j=1}^{K+1}{{\widehat{\Xi }}_{s}^{j}\left(-{\text{ln}}\left(1-{\left({\overline{\overline{\mathbb{T}}}}_{{\widehat{\mathbb{H}}}_{r}}^{j}\right)}^{\pi }\right)\right)}^{\varnothing } \right)}^{\frac{1}{\varnothing }}}\right)}^{\frac{1}{\pi }}{\mathcal{e}}^{i2\Pi {\left(1-{\mathcal{e}}^{-{\left({\sum }_{j=1}^{K+1}{{\widehat{\Xi }}_{s}^{j}\left(-{\text{ln}}\left(1-{\left({\overline{\overline{\mathbb{T}}}}_{{\widehat{\mathbb{H}}}_{i}}^{j}\right)}^{\pi }\right)\right)}^{\varnothing } \right)}^{\frac{1}{\varnothing }}}\right)}^{\frac{1}{\pi }}},\\ {\left({\mathcal{e}}^{-{\left({\sum }_{j=1}^{K+1}{{\widehat{\Xi }}_{s}^{j}\left(-{\text{ln}}\left(1-{\left({\overline{\overline{\mathbb{V}}}}_{{\widehat{\mathbb{H}}}_{r}}^{j}\right)}^{\pi }\right)\right)}^{\varnothing } \right)}^{\frac{1}{\varnothing }}}\right)}^{\frac{1}{\pi }} {\mathcal{e}}^{i2\Pi {\left({\mathcal{e}}^{-{\left({\sum }_{j=1}^{K+1}{{\widehat{\Xi }}_{s}^{j}\left(-{\text{ln}}\left(1-{\left({\overline{\overline{\mathbb{V}}}}_{{\widehat{\mathbb{H}}}_{a}}^{j}\right)}^{\pi }\right)\right)}^{\varnothing } \right)}^{\frac{1}{\varnothing }}}\right)}^{\frac{1}{\pi }}},\end{array}}{{\left({\mathcal{e}}^{-{\left({\sum }_{j=1}^{K+1}{{\widehat{\Xi }}_{s}^{j}\left(-{\text{ln}}\left(1-{\left({\overline{\overline{\mathbb{U}}}}_{{\widehat{\mathbb{H}}}_{r}}^{j}\right)}^{\pi }\right)\right)}^{\varnothing } \right)}^{\frac{1}{\varnothing }}}\right)}^{\frac{1}{\pi }} {\mathcal{e}}^{i2\Pi {\left({\mathcal{e}}^{-{\left({\sum }_{j=1}^{K+1}{{\widehat{\Xi }}_{s}^{j}\left(-{\text{ln}}\left(1-{\left({\overline{\overline{\mathbb{U}}}}_{{\widehat{\mathbb{H}}}_{i}}^{j}\right)}^{\pi }\right)\right)}^{\varnothing } \right)}^{\frac{1}{\varnothing }}}\right)}^{\frac{1}{\pi }}}}\end{array}\right)\\ \end{array}$$

Hence, we can prove our result successfully under the above consideration. Furthermore, we expressed the idea of idempotency, monotonicity, and boundness under the given techniques.

### Proposition 1

For any CTSHFNs, we have

1. Idempotency: When ${\widehat{\mathbb{H}}}_{p}^{j}= \left({\overline{\overline{\mathbb{T}}}}_{{\widehat{\mathbb{H}}}_{p}},{\overline{\overline{\mathbb{V}}}}_{{\widehat{\mathbb{H}}}_{p}},{\overline{\overline{\mathbb{U}}}}_{{\widehat{\mathbb{H}}}_{p}}\right)=\left(\left\{{\overline{\overline{\mathbb{T}}}}_{{\widehat{\mathbb{H}}}_{r}}^{j}{\mathcal{e}}^{i2\Pi \left({\overline{\overline{\mathbb{T}}}}_{{\widehat{\mathbb{H}}}_{i}}^{j}\right)}\right\},\left\{{\overline{\overline{\mathbb{V}}}}_{{\widehat{\mathbb{H}}}_{r}}^{j}{\mathcal{e}}^{i2\Pi \left({\overline{\overline{\mathbb{V}}}}_{{\widehat{\mathbb{H}}}_{a}}^{j}\right)}\right\},\left\{{\overline{\overline{\mathbb{U}}}}_{{\widehat{\mathbb{H}}}_{r}}^{j}{\mathcal{e}}^{i2\Pi \left({\overline{\overline{\mathbb{U}}}}_{{\widehat{\mathbb{H}}}_{i}}^{j}\right)}\right\}\right),$ thus

$$CTSHFAAWA\left({\widehat{\mathbb{H}}}_{p}^{1},{\widehat{\mathbb{H}}}_{p}^{2},\dots ,{\widehat{\mathbb{H}}}_{p}^{n}\right)={\widehat{\mathbb{H}}}_{p}$$

2. Monotonicity: When ${\widehat{\mathbb{H}}}_{p}^{j}\le {{\widehat{\mathbb{H}}}^{*j}}_{p}$ thus

$$CTSHFAAWA\left({\widehat{\mathbb{H}}}_{p}^{1},{\widehat{\mathbb{H}}}_{p}^{2},\dots ,{\widehat{\mathbb{H}}}_{p}^{n}\right)\le {\text{CTSHFAAWA}}\left({{\widehat{\mathbb{H}}}^{*1}}_{p} ,{{\widehat{\mathbb{H}}}^{*2}}_{p} ,\dots ,{{\widehat{\mathbb{H}}}^{*n}}_{p}\right)$$

3. Boundness: When $${\widehat{\mathbb{H}}}_{p}^{-}=\left(min{\overline{\overline{\mathbb{T}}}}_{{\widehat{\mathbb{H}}}_{p}},max{\overline{\overline{\mathbb{V}}}}_{{\widehat{\mathbb{H}}}_{p}},max{\overline{\overline{\mathbb{U}}}}_{{\widehat{\mathbb{H}}}_{p}}\right)$$=

$$\left(\left\{\underset{j}{\mathit{min}}{\overline{\overline{\mathbb{T}}}}_{{\widehat{\mathbb{H}}}_{r}}^{j}{e}^{i2\Pi \left(\underset{j}{\mathit{min}}{\overline{\overline{\mathbb{T}}}}_{{\widehat{\mathbb{H}}}_{i}}^{j}\right)}\right\},\left\{\underset{j}{\mathit{max}}{\overline{\overline{\mathbb{V}}}}_{{\widehat{\mathbb{H}}}_{r}}^{j}{e}^{i2\Pi \left(\underset{j}{\mathit{max}}{\overline{\overline{\mathbb{V}}}}_{{\widehat{\mathbb{H}}}_{a}}^{j}\right)}\right\},\left\{\underset{j}{\mathit{max}}{\overline{\overline{\mathbb{U}}}}_{{\widehat{\mathbb{H}}}_{r}}^{j}{e}^{i2\Pi \left(\underset{j}{\mathit{max}}{\overline{\overline{\mathbb{U}}}}_{{\widehat{\mathbb{H}}}_{i}}^{j}\right)}\right\}\right)$$ And $${\widehat{\mathbb{H}}}_{p}^{+}=\left(\mathit{max}{\overline{\overline{\mathbb{T}}}}_{{\widehat{\mathbb{H}}}_{p}},\mathit{min}{\overline{\overline{\mathbb{V}}}}_{{\widehat{\mathbb{H}}}_{p}},\mathit{min}{\overline{\overline{\mathbb{U}}}}_{{\widehat{\mathbb{H}}}_{p}}\right)=\left(\left\{\underset{j}{\mathit{max}}{\overline{\overline{\mathbb{T}}}}_{{\widehat{\mathbb{H}}}_{r}}^{j}{e}^{i2\Pi \left(\underset{j}{\mathit{max}}{\overline{\overline{\mathbb{T}}}}_{{\widehat{\mathbb{H}}}_{i}}^{j}\right)}\right\},\left\{\underset{j}{\mathit{min}}{\overline{\overline{\mathbb{V}}}}_{{\widehat{\mathbb{H}}}_{r}}^{j}{e}^{i2\Pi \left(\underset{j}{\mathit{min}}{\overline{\overline{\mathbb{V}}}}_{{\widehat{\mathbb{H}}}_{a}}^{j}\right)}\right\},\left\{\underset{j}{\mathit{min}}{\overline{\overline{\mathbb{U}}}}_{{\widehat{\mathbb{H}}}_{r}}^{j}{e}^{i2\Pi \left(\underset{j}{\mathit{min}}{\overline{\overline{\mathbb{U}}}}_{{\widehat{\mathbb{H}}}_{i}}^{j}\right)}\right\}\right)$$, thus

$${\widehat{\mathbb{H}}}_{p}^{-}\le CSHFAAWA\left({\widehat{\mathbb{H}}}_{p}^{1},{\widehat{\mathbb{H}}}_{p}^{2},\dots ,{\widehat{\mathbb{H}}}_{p}^{n}\right)\le {\widehat{\mathbb{H}}}_{p}^{+}$$

### Proof

Straightforward.□

### Definition 11

The mathematical information

$$CTSHFAAOWPA\left({\widehat{\mathbb{H}}}_{p}^{1},{\widehat{\mathbb{H}}}_{p}^{2},\dots ,{\widehat{\mathbb{H}}}_{p}^{n}\right)={\widehat{\Xi }}_{s}^{1}{\widehat{\mathbb{H}}}_{p}^{o\left(1\right)}\oplus{\widehat{\Xi }}_{s}^{2}{\widehat{\mathbb{H}}}_{p}^{o\left(2\right)}\oplus\dots \oplus{\widehat{\Xi }}_{s}{\widehat{\mathbb{H}}}_{p}^{o\left({\text{n}}\right)}={\oplus}_{j=1}^{n}\left({\widehat{\Xi }}_{s}^{j}{\widehat{\mathbb{H}}}_{p}^{o\left({\text{j}}\right)}\right)$$

Represents $CTSHFAAOWPA$ operator such as

$${\widehat{\Xi }}_{s}^{i}=\frac{1+T\left({\mathbb{A}}_{i}\right)}{\sum_{i=1}^{n}\left(1+T\left({\mathbb{A}}_{i}\right)\right)},i=\mathrm{1,2},\dots ,n$$

Shown the power operator which is used as a weight vector.

### Theorem 2

Information in Def. (11), we derive that the aggregated information of the above theory is again a CTSHFN, such as

$$CTSHFAAOWPA\left({\widehat{\mathbb{H}}}_{p}^{1},{\widehat{\mathbb{H}}}_{p}^{2},\dots ,{\widehat{\mathbb{H}}}_{p}^{n}\right)=\coprod_{\left(\begin{array}{c}{\overline{\overline{\mathbb{T}}}}_{{\widehat{\mathbb{H}}}_{r}}^{j},{\overline{\overline{\mathbb{T}}}}_{{\widehat{\mathbb{H}}}_{i}}^{j}\in {\overline{\overline{\mathbb{T}}}}_{{\widehat{\mathbb{H}}}_{\begin{array}{c}p\end{array}}}\\ {\overline{\overline{\mathbb{V}}}}_{{\widehat{\mathbb{H}}}_{r}}^{j},{\overline{\overline{\mathbb{V}}}}_{{\widehat{\mathbb{H}}}_{a}}^{j}\in {\overline{\overline{\mathbb{V}}}}_{{\widehat{\mathbb{H}}}_{\begin{array}{c}p\end{array}}}\\ {\overline{\overline{\mathbb{U}}}}_{{\widehat{\mathbb{H}}}_{r}}^{j},{\overline{\overline{\mathbb{U}}}}_{{\widehat{\mathbb{H}}}_{i}}^{j}\in {\overline{\overline{\mathbb{U}}}}_{{\widehat{\mathbb{H}}}_{\begin{array}{c}p\end{array}}}\end{array}\right)}\left(\begin{array}{c}\genfrac{}{}{0pt}{}{\begin{array}{c}{\left(1-{\mathcal{e}}^{-{\left({\sum }_{j=1}^{n}{{\widehat{\Xi }}_{s}^{j}\left(-{\text{ln}}\left(1-{\left({\overline{\overline{\mathbb{T}}}}_{{\widehat{\mathbb{H}}}_{r}}^{o(j)}\right)}^{\pi }\right)\right)}^{\varnothing } \right)}^{\frac{1}{\varnothing }}}\right)}^{\frac{1}{\pi }}{\mathcal{e}}^{i2\Pi {\left(1-{\mathcal{e}}^{-{\left({\sum }_{j=1}^{n}{{\widehat{\Xi }}_{s}^{j}\left(-{\text{ln}}\left(1-{\left({\overline{\overline{\mathbb{T}}}}_{{\widehat{\mathbb{H}}}_{i}}^{o(j)}\right)}^{\pi }\right)\right)}^{\varnothing } \right)}^{\frac{1}{\varnothing }}}\right)}^{\frac{1}{\pi }}},\\ {\left({\mathcal{e}}^{-{\left({\sum }_{j=1}^{n}{{\widehat{\Xi }}_{s}^{j}\left(-{\text{ln}}\left(1-{\left({\overline{\overline{\mathbb{V}}}}_{{\widehat{\mathbb{H}}}_{r}}^{o(j)}\right)}^{\pi }\right)\right)}^{\varnothing } \right)}^{\frac{1}{\varnothing }}}\right)}^{\frac{1}{\pi }} {\mathcal{e}}^{i2\Pi {\left({\mathcal{e}}^{-{\left({\sum }_{j=1}^{n}{{\widehat{\Xi }}_{s}^{j}\left(-{\text{ln}}\left(1-{\left({\overline{\overline{\mathbb{V}}}}_{{\widehat{\mathbb{H}}}_{a}}^{o(j)}\right)}^{\pi }\right)\right)}^{\varnothing } \right)}^{\frac{1}{\varnothing }}}\right)}^{\frac{1}{\pi }}},\end{array}}{{\left({\mathcal{e}}^{-{\left({\sum }_{j=1}^{n}{{\widehat{\Xi }}_{s}^{j}\left(-{\text{ln}}\left(1-{\left({\overline{\overline{\mathbb{U}}}}_{{\widehat{\mathbb{H}}}_{r}}^{o(j)}\right)}^{\pi }\right)\right)}^{\varnothing } \right)}^{\frac{1}{\varnothing }}}\right)}^{\frac{1}{\pi }} {\mathcal{e}}^{i2\Pi {\left({\mathcal{e}}^{-{\left({\sum }_{j=1}^{n}{{\widehat{\Xi }}_{s}^{j}\left(-{\text{ln}}\left(1-{\left({\overline{\overline{\mathbb{U}}}}_{{\widehat{\mathbb{H}}}_{i}}^{o(j)}\right)}^{\pi }\right)\right)}^{\varnothing } \right)}^{\frac{1}{\varnothing }}}\right)}^{\frac{1}{\pi }}}}\end{array}\right)$$

### Proof

Straightforward. Additionally, we observed the purpose of idempotency, monotonicity, and boundness for the given techniques.□

### Proposition 2

A CTSHFN initiates ${\widehat{\mathbb{H}}}_{p}^{j}= \left({\overline{\overline{\mathbb{T}}}}_{{\widehat{\mathbb{H}}}_{p}},{\overline{\overline{\mathbb{V}}}}_{{\widehat{\mathbb{H}}}_{p}},{\overline{\overline{\mathbb{U}}}}_{{\widehat{\mathbb{H}}}_{p}}\right)=\left(\left\{{\overline{\overline{\mathbb{T}}}}_{{\widehat{\mathbb{H}}}_{r}}^{j}{\mathcal{e}}^{i2\Pi \left({\overline{\overline{\mathbb{T}}}}_{{\widehat{\mathbb{H}}}_{i}}^{j}\right)}\right\},\left\{{\overline{\overline{\mathbb{V}}}}_{{\widehat{\mathbb{H}}}_{r}}^{j}{\mathcal{e}}^{i2\Pi \left({\overline{\overline{\mathbb{V}}}}_{{\widehat{\mathbb{H}}}_{a}}^{j}\right)}\right\},\left\{{\overline{\overline{\mathbb{U}}}}_{{\widehat{\mathbb{H}}}_{r}}^{j}{\mathcal{e}}^{i2\Pi \left({\overline{\overline{\mathbb{U}}}}_{{\widehat{\mathbb{H}}}_{i}}^{j}\right)}\right\}\right),j=\mathrm{1,2},\dots ,n$. Then we observe that

1. Idempotency: When ${\widehat{\mathbb{H}}}_{p}^{j}={\widehat{\mathbb{H}}}_{p}= \left({\overline{\overline{\mathbb{T}}}}_{{\widehat{\mathbb{H}}}_{p}},{\overline{\overline{\mathbb{V}}}}_{{\widehat{\mathbb{H}}}_{p}},{\overline{\overline{\mathbb{U}}}}_{{\widehat{\mathbb{H}}}_{p}}\right)=\left(\left\{{\overline{\overline{\mathbb{T}}}}_{{\widehat{\mathbb{H}}}_{r}}^{j}{\mathcal{e}}^{i2\Pi \left({\overline{\overline{\mathbb{T}}}}_{{\widehat{\mathbb{H}}}_{i}}^{j}\right)}\right\},\left\{{\overline{\overline{\mathbb{V}}}}_{{\widehat{\mathbb{H}}}_{r}}^{j}{\mathcal{e}}^{i2\Pi \left({\overline{\overline{\mathbb{V}}}}_{{\widehat{\mathbb{H}}}_{a}}^{j}\right)}\right\},\left\{{\overline{\overline{\mathbb{U}}}}_{{\widehat{\mathbb{H}}}_{r}}^{j}{\mathcal{e}}^{i2\Pi \left({\overline{\overline{\mathbb{U}}}}_{{\widehat{\mathbb{H}}}_{i}}^{j}\right)}\right\}\right),$ thus

$$CTSHFAAOWPA\left({\widehat{\mathbb{H}}}_{p}^{1},{\widehat{\mathbb{H}}}_{p}^{2},\dots ,{\widehat{\mathbb{H}}}_{p}^{n}\right)={\widehat{\mathbb{H}}}_{p}$$

2. Monotonicity: When ${\widehat{\mathbb{H}}}_{p}^{j}\le {{\widehat{\mathbb{H}}}^{*j}}_{p}$ thus

$$CTSHFAAOWPA\left({\widehat{\mathbb{H}}}_{p}^{1},{\widehat{\mathbb{H}}}_{p}^{2},\dots ,{\widehat{\mathbb{H}}}_{p}^{n}\right)\le {\text{CTSHFAAOWPA}}\left({{\widehat{\mathbb{H}}}^{*1}}_{p} ,{{\widehat{\mathbb{H}}}^{*2}}_{p} ,\dots ,{{\widehat{\mathbb{H}}}^{*n}}_{p}\right)$$

3. Boundness: When $${\widehat{\mathbb{H}}}_{p}^{-}=\left(min{\overline{\overline{\mathbb{T}}}}_{{\widehat{\mathbb{H}}}_{p}},max{\overline{\overline{\mathbb{V}}}}_{{\widehat{\mathbb{H}}}_{p}},max{\overline{\overline{\mathbb{U}}}}_{{\widehat{\mathbb{H}}}_{p}}\right)$$=

$$\left(\left\{\underset{j}{\mathit{min}}{\overline{\overline{\mathbb{T}}}}_{{\widehat{\mathbb{H}}}_{r}}^{j}{e}^{i2\Pi \left(\underset{j}{\mathit{min}}{\overline{\overline{\mathbb{T}}}}_{{\widehat{\mathbb{H}}}_{i}}^{j}\right)}\right\},\left\{\underset{j}{\mathit{max}}{\overline{\overline{\mathbb{V}}}}_{{\widehat{\mathbb{H}}}_{r}}^{j}{e}^{i2\Pi \left(\underset{j}{\mathit{max}}{\overline{\overline{\mathbb{V}}}}_{{\widehat{\mathbb{H}}}_{a}}^{j}\right)}\right\},\left\{\underset{j}{\mathit{max}}{\overline{\overline{\mathbb{U}}}}_{{\widehat{\mathbb{H}}}_{r}}^{j}{e}^{i2\Pi \left(\underset{j}{\mathit{max}}{\overline{\overline{\mathbb{U}}}}_{{\widehat{\mathbb{H}}}_{i}}^{j}\right)}\right\}\right)$$ And $${\widehat{\mathbb{H}}}_{p}^{+}=\left(\mathit{max}{\overline{\overline{\mathbb{T}}}}_{{\widehat{\mathbb{H}}}_{p}},\mathit{min}{\overline{\overline{\mathbb{V}}}}_{{\widehat{\mathbb{H}}}_{p}},\mathit{min}{\overline{\overline{\mathbb{U}}}}_{{\widehat{\mathbb{H}}}_{p}}\right)=\left(\left\{\underset{j}{\mathit{max}}{\overline{\overline{\mathbb{T}}}}_{{\widehat{\mathbb{H}}}_{r}}^{j}{e}^{i2\Pi \left(\underset{j}{\mathit{max}}{\overline{\overline{\mathbb{T}}}}_{{\widehat{\mathbb{H}}}_{i}}^{j}\right)}\right\},\left\{\underset{j}{\mathit{min}}{\overline{\overline{\mathbb{V}}}}_{{\widehat{\mathbb{H}}}_{r}}^{j}{e}^{i2\Pi \left(\underset{j}{\mathit{min}}{\overline{\overline{\mathbb{V}}}}_{{\widehat{\mathbb{H}}}_{a}}^{j}\right)}\right\},\left\{\underset{j}{\mathit{min}}{\overline{\overline{\mathbb{U}}}}_{{\widehat{\mathbb{H}}}_{r}}^{j}{e}^{i2\Pi \left(\underset{j}{\mathit{min}}{\overline{\overline{\mathbb{U}}}}_{{\widehat{\mathbb{H}}}_{i}}^{j}\right)}\right\}\right)$$, thus

$${\widehat{\mathbb{H}}}_{p}^{-}\le CSHFAAOWPA\left({\widehat{\mathbb{H}}}_{p}^{1},{\widehat{\mathbb{H}}}_{p}^{2},\dots ,{\widehat{\mathbb{H}}}_{p}^{n}\right)\le {\widehat{\mathbb{H}}}_{p}^{+}$$

### Proof

Straightforward.□

### Definition 12

The mathematical information

$$CTSHFAAOWPG\left({\widehat{\mathbb{H}}}_{p}^{1},{\widehat{\mathbb{H}}}_{p}^{2},\dots ,{\widehat{\mathbb{H}}}_{p}^{n}\right)={\widehat{\mathbb{H}}}_{p}^{{{1}{\widehat{\Xi }}_{s}^{1}}}\otimes {\widehat{\mathbb{H}}}_{p}^{{{2}{\widehat{\Xi }}_{s}^{2}}}\otimes \dots \otimes {\widehat{\mathbb{H}}}_{p}^{{{n}{\widehat{\Xi }}_{s}^{n}}}={\otimes }_{j=1}^{n}\left({\widehat{\mathbb{H}}}_{p}^{{{j}{\widehat{\Xi }}_{s}^{j}}}\right)$$

Represents the CTSHFAAWPA operator, such as

$${\widehat{\Xi }}_{s}^{i}=\frac{1+T\left({\mathbb{A}}_{i}\right)}{\sum_{i=1}^{n}\left(1+T\left({\mathbb{A}}_{i}\right)\right)},i=\mathrm{1,2},\dots ,n$$

Shown the power operator which is used as a weight vector.

### Theorem 3

Information in Def. (12), we derive that the aggregated information of the above theory is again a CTSHFN, such as

$$CTSHFAAWPG\left({\widehat{\mathbb{H}}}_{p}^{1},{\widehat{\mathbb{H}}}_{p}^{2},\dots ,{\widehat{\mathbb{H}}}_{p}^{n}\right)=\coprod_{\left(\begin{array}{c}{\overline{\overline{\mathbb{T}}}}_{{\widehat{\mathbb{H}}}_{r}}^{j},{\overline{\overline{\mathbb{T}}}}_{{\widehat{\mathbb{H}}}_{i}}^{j}\in {\overline{\overline{\mathbb{T}}}}_{{\widehat{\mathbb{H}}}_{\begin{array}{c}p\end{array}}}\\ {\overline{\overline{\mathbb{V}}}}_{{\widehat{\mathbb{H}}}_{r}}^{j},{\overline{\overline{\mathbb{V}}}}_{{\widehat{\mathbb{H}}}_{a}}^{j}\in {\overline{\overline{\mathbb{V}}}}_{{\widehat{\mathbb{H}}}_{\begin{array}{c}p\end{array}}}\\ {\overline{\overline{\mathbb{U}}}}_{{\widehat{\mathbb{H}}}_{r}}^{j},{\overline{\overline{\mathbb{U}}}}_{{\widehat{\mathbb{H}}}_{i}}^{j}\in {\overline{\overline{\mathbb{U}}}}_{{\widehat{\mathbb{H}}}_{\begin{array}{c}p\end{array}}}\end{array}\right)}\begin{array}{c}\left(\begin{array}{c}\genfrac{}{}{0pt}{}{\begin{array}{c}{\left({\mathcal{e}}^{-{\left({\sum }_{j=1}^{n}{{\widehat{\Xi }}_{s}^{j}\left(-{\text{ln}}\left({\left({\overline{\overline{\mathbb{T}}}}_{{\widehat{\mathbb{H}}}_{r}}^{j}\right)}^{\pi }\right)\right)}^{\varnothing } \right)}^{\frac{1}{\varnothing }}}\right)}^{\frac{1}{\pi }}{\mathcal{e}}^{i2\Pi {\left({\mathcal{e}}^{-{\left({\sum }_{j=1}^{n}{{\widehat{\Xi }}_{s}^{j}\left(-{\text{ln}}\left({\left({\overline{\overline{\mathbb{T}}}}_{{\widehat{\mathbb{H}}}_{i}}^{j}\right)}^{\pi }\right)\right)}^{\varnothing } \right)}^{\frac{1}{\varnothing }}}\right)}^{\frac{1}{\pi }}},\\ {\left(1-{\mathcal{e}}^{-{\left({\sum }_{j=1}^{n}{{\widehat{\Xi }}_{s}^{j}\left(-{\text{ln}}\left(1-{\left({\overline{\overline{\mathbb{V}}}}_{{\widehat{\mathbb{H}}}_{r}}^{j}\right)}^{\pi }\right)\right)}^{\varnothing } \right)}^{\frac{1}{\varnothing }}}\right)}^{\frac{1}{\pi }} {\mathcal{e}}^{i2\Pi {\left(1-{\mathcal{e}}^{-{\left({\sum }_{j=1}^{n}{{\widehat{\Xi }}_{s}^{j}\left(-{\text{ln}}\left(1-{\left({\overline{\overline{\mathbb{V}}}}_{{\widehat{\mathbb{H}}}_{a}}^{j}\right)}^{\pi }\right)\right)}^{\varnothing } \right)}^{\frac{1}{\varnothing }}}\right)}^{\frac{1}{\pi }}},\end{array}}{{\left({\mathcal{e}}^{-{\left({\sum }_{j=1}^{k}{{\widehat{\Xi }}_{s}^{j}\left(-{\text{ln}}\left(1-{\left({\overline{\overline{\mathbb{U}}}}_{{\widehat{\mathbb{H}}}_{r}}^{j}\right)}^{\pi }\right)\right)}^{\varnothing } \right)}^{\frac{1}{\varnothing }}}\right)}^{\frac{1}{\pi }} {\mathcal{e}}^{i2\Pi {\left(1-{\mathcal{e}}^{-{\left({\sum }_{j=1}^{n}{{\widehat{\Xi }}_{s}^{j}\left(-{\text{ln}}\left(1-{\left({\overline{\overline{\mathbb{U}}}}_{{\widehat{\mathbb{H}}}_{i}}^{j}\right)}^{\pi }\right)\right)}^{\varnothing } \right)}^{\frac{1}{\varnothing }}}\right)}^{\frac{1}{\pi }}}}\end{array}\right)\\ \end{array}$$

### Proof

Straightforward. Additionally, we observed the main purpose of idempotency, monotonicity, and boundness for the given techniques.□

### Proposition 3

A CTSHFNs commence ${\widehat{\mathbb{H}}}_{p}^{j}= \left({\overline{\overline{\mathbb{T}}}}_{{\widehat{\mathbb{H}}}_{p}},{\overline{\overline{\mathbb{V}}}}_{{\widehat{\mathbb{H}}}_{p}},{\overline{\overline{\mathbb{U}}}}_{{\widehat{\mathbb{H}}}_{p}}\right)=\left(\left\{{\overline{\overline{\mathbb{T}}}}_{{\widehat{\mathbb{H}}}_{r}}^{j}{\mathcal{e}}^{i2\Pi \left({\overline{\overline{\mathbb{T}}}}_{{\widehat{\mathbb{H}}}_{i}}^{j}\right)}\right\},\left\{{\overline{\overline{\mathbb{V}}}}_{{\widehat{\mathbb{H}}}_{r}}^{j}{\mathcal{e}}^{i2\Pi \left({\overline{\overline{\mathbb{V}}}}_{{\widehat{\mathbb{H}}}_{a}}^{j}\right)}\right\},\left\{{\overline{\overline{\mathbb{U}}}}_{{\widehat{\mathbb{H}}}_{r}}^{j}{\mathcal{e}}^{i2\Pi \left({\overline{\overline{\mathbb{U}}}}_{{\widehat{\mathbb{H}}}_{i}}^{j}\right)}\right\}\right),j=\mathrm{1,2},\dots ,n$. Then we observe the following properties.

1. Idempotency: When ${\widehat{\mathbb{H}}}_{p}^{j}={\widehat{\mathbb{H}}}_{p}= \left({\overline{\overline{\mathbb{T}}}}_{{\widehat{\mathbb{H}}}_{p}},{\overline{\overline{\mathbb{V}}}}_{{\widehat{\mathbb{H}}}_{p}},{\overline{\overline{\mathbb{U}}}}_{{\widehat{\mathbb{H}}}_{p}}\right)=\left(\left\{{\overline{\overline{\mathbb{T}}}}_{{\widehat{\mathbb{H}}}_{r}}^{j}{\mathcal{e}}^{i2\Pi \left({\overline{\overline{\mathbb{T}}}}_{{\widehat{\mathbb{H}}}_{i}}^{j}\right)}\right\},\left\{{\overline{\overline{\mathbb{V}}}}_{{\widehat{\mathbb{H}}}_{r}}^{j}{\mathcal{e}}^{i2\Pi \left({\overline{\overline{\mathbb{V}}}}_{{\widehat{\mathbb{H}}}_{a}}^{j}\right)}\right\},\left\{{\overline{\overline{\mathbb{U}}}}_{{\widehat{\mathbb{H}}}_{r}}^{j}{\mathcal{e}}^{i2\Pi \left({\overline{\overline{\mathbb{U}}}}_{{\widehat{\mathbb{H}}}_{i}}^{j}\right)}\right\}\right),$ thus

$$CTSHFAAWPG\left({\widehat{\mathbb{H}}}_{p}^{1},{\widehat{\mathbb{H}}}_{p}^{2},\dots ,{\widehat{\mathbb{H}}}_{p}^{n}\right)={\widehat{\mathbb{H}}}_{p}$$

2. Monotonicity: When ${\widehat{\mathbb{H}}}_{p}^{j}\le {{\widehat{\mathbb{H}}}^{*j}}_{p}$ thus

$$CTSHFAAWPG\left({\widehat{\mathbb{H}}}_{p}^{1},{\widehat{\mathbb{H}}}_{p}^{2},\dots ,{\widehat{\mathbb{H}}}_{p}^{n}\right)\le {\text{CTSHFAAWPG}}\left({{\widehat{\mathbb{H}}}^{*1}}_{p} ,{{\widehat{\mathbb{H}}}^{*2}}_{p} ,\dots ,{{\widehat{\mathbb{H}}}^{*n}}_{p}\right)$$

3. Boundness: When $${\widehat{\mathbb{H}}}_{p}^{-}=\left(min{\overline{\overline{\mathbb{T}}}}_{{\widehat{\mathbb{H}}}_{p}},max{\overline{\overline{\mathbb{V}}}}_{{\widehat{\mathbb{H}}}_{p}},max{\overline{\overline{\mathbb{U}}}}_{{\widehat{\mathbb{H}}}_{p}}\right)$$=

$$\left(\left\{\underset{j}{\mathit{min}}{\overline{\overline{\mathbb{T}}}}_{{\widehat{\mathbb{H}}}_{r}}^{j}{e}^{i2\Pi \left(\underset{j}{\mathit{min}}{\overline{\overline{\mathbb{T}}}}_{{\widehat{\mathbb{H}}}_{i}}^{j}\right)}\right\},\left\{\underset{j}{\mathit{max}}{\overline{\overline{\mathbb{V}}}}_{{\widehat{\mathbb{H}}}_{r}}^{j}{e}^{i2\Pi \left(\underset{j}{\mathit{max}}{\overline{\overline{\mathbb{V}}}}_{{\widehat{\mathbb{H}}}_{a}}^{j}\right)}\right\},\left\{\underset{j}{\mathit{max}}{\overline{\overline{\mathbb{U}}}}_{{\widehat{\mathbb{H}}}_{r}}^{j}{e}^{i2\Pi \left(\underset{j}{\mathit{max}}{\overline{\overline{\mathbb{U}}}}_{{\widehat{\mathbb{H}}}_{i}}^{j}\right)}\right\}\right)$$ And $${\widehat{\mathbb{H}}}_{p}^{+}=\left(\mathit{max}{\overline{\overline{\mathbb{T}}}}_{{\widehat{\mathbb{H}}}_{p}},\mathit{min}{\overline{\overline{\mathbb{V}}}}_{{\widehat{\mathbb{H}}}_{p}},\mathit{min}{\overline{\overline{\mathbb{U}}}}_{{\widehat{\mathbb{H}}}_{p}}\right)=\left(\left\{\underset{j}{\mathit{max}}{\overline{\overline{\mathbb{T}}}}_{{\widehat{\mathbb{H}}}_{r}}^{j}{e}^{i2\Pi \left(\underset{j}{\mathit{max}}{\overline{\overline{\mathbb{T}}}}_{{\widehat{\mathbb{H}}}_{i}}^{j}\right)}\right\},\left\{\underset{j}{\mathit{min}}{\overline{\overline{\mathbb{V}}}}_{{\widehat{\mathbb{H}}}_{r}}^{j}{e}^{i2\Pi \left(\underset{j}{\mathit{min}}{\overline{\overline{\mathbb{V}}}}_{{\widehat{\mathbb{H}}}_{a}}^{j}\right)}\right\},\left\{\underset{j}{\mathit{min}}{\overline{\overline{\mathbb{U}}}}_{{\widehat{\mathbb{H}}}_{r}}^{j}{e}^{i2\Pi \left(\underset{j}{\mathit{min}}{\overline{\overline{\mathbb{U}}}}_{{\widehat{\mathbb{H}}}_{i}}^{j}\right)}\right\}\right)$$, thus

$${\widehat{\mathbb{H}}}_{p}^{-}\le CPHFAAWPG\left({\widehat{\mathbb{H}}}_{p}^{1},{\widehat{\mathbb{H}}}_{p}^{2},\dots ,{\widehat{\mathbb{H}}}_{p}^{n}\right)\le {\widehat{\mathbb{H}}}_{p}^{+}$$

### Proof

Straightforward.□

### Definition 13

The mathematical information

$$CTSHFAAOWPG\left({\widehat{\mathbb{H}}}_{p}^{1},{\widehat{\mathbb{H}}}_{p}^{2},\dots ,{\widehat{\mathbb{H}}}_{p}^{n}\right)={\widehat{\mathbb{H}}}_{p}^{{o{\left(1\right)}{\widehat{\Xi }}_{s}^{1}}}\otimes {\widehat{\mathbb{H}}}_{p}^{{o{\left(1\right)}{\widehat{\Xi }}_{s}^{2}}}\otimes \dots \otimes {\widehat{\mathbb{H}}}_{p}^{{o{\left(1\right)}{\widehat{\Xi }}_{s}^{n}}}={\otimes }_{j=1}^{n}\left({\widehat{\mathbb{H}}}_{p}^{{o{\left(1\right)}{\widehat{\Xi }}_{s}^{j}}}\right)$$

Represents the CTSHFAAWPA operator, such as

$${\widehat{\Xi }}_{s}^{i}=\frac{1+T\left({\mathbb{A}}_{i}\right)}{\sum_{i=1}^{n}\left(1+T\left({\mathbb{A}}_{i}\right)\right)},i=\mathrm{1,2},\dots ,n$$

Shown the power operator which is used as a weight vector.

### Theorem 4

Information in Def. (13), we derive that the aggregated information of the above theory is again a CTSHFN, such as

$$CTSHFAAOWPG\left({\widehat{\mathbb{H}}}_{p}^{1},{\widehat{\mathbb{H}}}_{p}^{2},\dots ,{\widehat{\mathbb{H}}}_{p}^{n}\right)=\coprod_{\left(\begin{array}{c}{\overline{\overline{\mathbb{T}}}}_{{\widehat{\mathbb{H}}}_{r}}^{j},{\overline{\overline{\mathbb{T}}}}_{{\widehat{\mathbb{H}}}_{i}}^{j}\in {\overline{\overline{\mathbb{T}}}}_{{\widehat{\mathbb{H}}}_{\begin{array}{c}p\end{array}}}\\ {\overline{\overline{\mathbb{V}}}}_{{\widehat{\mathbb{H}}}_{r}}^{j},{\overline{\overline{\mathbb{V}}}}_{{\widehat{\mathbb{H}}}_{a}}^{j}\in {\overline{\overline{\mathbb{V}}}}_{{\widehat{\mathbb{H}}}_{\begin{array}{c}p\end{array}}}\\ {\overline{\overline{\mathbb{U}}}}_{{\widehat{\mathbb{H}}}_{r}}^{j},{\overline{\overline{\mathbb{U}}}}_{{\widehat{\mathbb{H}}}_{i}}^{j}\in {\overline{\overline{\mathbb{U}}}}_{{\widehat{\mathbb{H}}}_{\begin{array}{c}p\end{array}}}\end{array}\right)}\left(\begin{array}{c}\genfrac{}{}{0pt}{}{\begin{array}{c}{\left({\mathcal{e}}^{-{\left({\sum }_{j=1}^{n}{{\widehat{\Xi }}_{s}^{j}\left(-{\text{ln}}\left({\left({\overline{\overline{\mathbb{T}}}}_{{\widehat{\mathbb{H}}}_{r}}^{o(j)}\right)}^{\pi }\right)\right)}^{\varnothing } \right)}^{\frac{1}{\varnothing }}}\right)}^{\frac{1}{\pi }}{\mathcal{e}}^{i2\Pi {\left({\mathcal{e}}^{-{\left({\sum }_{j=1}^{n}{{\widehat{\Xi }}_{s}^{j}\left(-{\text{ln}}\left({\left({\overline{\overline{\mathbb{T}}}}_{{\widehat{\mathbb{H}}}_{i}}^{o(j)}\right)}^{\pi }\right)\right)}^{\varnothing } \right)}^{\frac{1}{\varnothing }}}\right)}^{\frac{1}{\pi }}},\\ {\left(1-{\mathcal{e}}^{-{\left({\sum }_{j=1}^{n}{{\widehat{\Xi }}_{s}^{j}\left(-{\text{ln}}\left(1-{\left({\overline{\overline{\mathbb{V}}}}_{{\widehat{\mathbb{H}}}_{r}}^{o(j)}\right)}^{\pi }\right)\right)}^{\varnothing } \right)}^{\frac{1}{\varnothing }}}\right)}^{\frac{1}{\pi }} {\mathcal{e}}^{i2\Pi {\left(1-{\mathcal{e}}^{-{\left({\sum }_{j=1}^{n}{{\widehat{\Xi }}_{s}^{j}\left(-{\text{ln}}\left(1-{\left({\overline{\overline{\mathbb{V}}}}_{{\widehat{\mathbb{H}}}_{a}}^{o(j)}\right)}^{\pi }\right)\right)}^{\varnothing } \right)}^{\frac{1}{\varnothing }}}\right)}^{\frac{1}{\pi }}},\end{array}}{{\left(1-{\mathcal{e}}^{-{\left({\sum }_{j=1}^{n}{{\widehat{\Xi }}_{s}^{j}\left(-{\text{ln}}\left(1-{\left({\overline{\overline{\mathbb{U}}}}_{{\widehat{\mathbb{H}}}_{r}}^{o(j)}\right)}^{\pi }\right)\right)}^{\varnothing } \right)}^{\frac{1}{\varnothing }}}\right)}^{\frac{1}{\pi }} {\mathcal{e}}^{i2\Pi {\left(1-{\mathcal{e}}^{-{\left({\sum }_{j=1}^{n}{{\widehat{\Xi }}_{s}^{j}\left(-{\text{ln}}\left(1-{\left({\overline{\overline{\mathbb{U}}}}_{{\widehat{\mathbb{H}}}_{i}}^{o(j)}\right)}^{\pi }\right)\right)}^{\varnothing } \right)}^{\frac{1}{\varnothing }}}\right)}^{\frac{1}{\pi }}}}\end{array}\right)$$

### Proof

Straightforward. Further, we observed the main purpose of idempotency, monotonicity, and boundness for the given techniques.□

### Proposition 4

Under the presence of CTSHFNs ${\widehat{\mathbb{H}}}_{p}^{j}= \left({\overline{\overline{\mathbb{T}}}}_{{\widehat{\mathbb{H}}}_{p}},{\overline{\overline{\mathbb{V}}}}_{{\widehat{\mathbb{H}}}_{p}},{\overline{\overline{\mathbb{U}}}}_{{\widehat{\mathbb{H}}}_{p}}\right)=\left(\left\{{\overline{\overline{\mathbb{T}}}}_{{\widehat{\mathbb{H}}}_{r}}^{j}{\mathcal{e}}^{i2\Pi \left({\overline{\overline{\mathbb{T}}}}_{{\widehat{\mathbb{H}}}_{i}}^{j}\right)}\right\},\left\{{\overline{\overline{\mathbb{V}}}}_{{\widehat{\mathbb{H}}}_{r}}^{j}{\mathcal{e}}^{i2\Pi \left({\overline{\overline{\mathbb{V}}}}_{{\widehat{\mathbb{H}}}_{a}}^{j}\right)}\right\},\left\{{\overline{\overline{\mathbb{U}}}}_{{\widehat{\mathbb{H}}}_{r}}^{j}{\mathcal{e}}^{i2\Pi \left({\overline{\overline{\mathbb{U}}}}_{{\widehat{\mathbb{H}}}_{i}}^{j}\right)}\right\}\right),j=\mathrm{1,2},\dots ,n$. Then we observed

1. Idempotency: When ${\widehat{\mathbb{H}}}_{p}^{j}={\widehat{\mathbb{H}}}_{p}= \left({\overline{\overline{\mathbb{T}}}}_{{\widehat{\mathbb{H}}}_{p}},{\overline{\overline{\mathbb{V}}}}_{{\widehat{\mathbb{H}}}_{p}},{\overline{\overline{\mathbb{U}}}}_{{\widehat{\mathbb{H}}}_{p}}\right)=\left(\left\{{\overline{\overline{\mathbb{T}}}}_{{\widehat{\mathbb{H}}}_{r}}^{j}{\mathcal{e}}^{i2\Pi \left({\overline{\overline{\mathbb{T}}}}_{{\widehat{\mathbb{H}}}_{i}}^{j}\right)}\right\},\left\{{\overline{\overline{\mathbb{V}}}}_{{\widehat{\mathbb{H}}}_{r}}^{j}{\mathcal{e}}^{i2\Pi \left({\overline{\overline{\mathbb{V}}}}_{{\widehat{\mathbb{H}}}_{a}}^{j}\right)}\right\},\left\{{\overline{\overline{\mathbb{U}}}}_{{\widehat{\mathbb{H}}}_{r}}^{j}{\mathcal{e}}^{i2\Pi \left({\overline{\overline{\mathbb{U}}}}_{{\widehat{\mathbb{H}}}_{i}}^{j}\right)}\right\}\right),$ thus

$$CTSHFAAOWPG\left({\widehat{\mathbb{H}}}_{p}^{1},{\widehat{\mathbb{H}}}_{p}^{2},\dots ,{\widehat{\mathbb{H}}}_{p}^{n}\right)={\widehat{\mathbb{H}}}_{p}$$

2. Monotonicity: When ${\widehat{\mathbb{H}}}_{p}^{j}\le {{\widehat{\mathbb{H}}}^{*j}}_{p}$ thus

$$CTSHFAAOWPG\left({\widehat{\mathbb{H}}}_{p}^{1},{\widehat{\mathbb{H}}}_{p}^{2},\dots ,{\widehat{\mathbb{H}}}_{p}^{n}\right)\le {\text{CTSHFAAOWPG}}\left({{\widehat{\mathbb{H}}}^{*1}}_{p} ,{{\widehat{\mathbb{H}}}^{*2}}_{p} ,\dots ,{{\widehat{\mathbb{H}}}^{*n}}_{p}\right)$$

3. Boundness: When $${\widehat{\mathbb{H}}}_{p}^{-}=\left(min{\overline{\overline{\mathbb{T}}}}_{{\widehat{\mathbb{H}}}_{p}},max{\overline{\overline{\mathbb{V}}}}_{{\widehat{\mathbb{H}}}_{p}},max{\overline{\overline{\mathbb{U}}}}_{{\widehat{\mathbb{H}}}_{p}}\right)$$=

$$\left(\left\{\underset{j}{\mathit{min}}{\overline{\overline{\mathbb{T}}}}_{{\widehat{\mathbb{H}}}_{r}}^{j}{e}^{i2\Pi \left(\underset{j}{\mathit{min}}{\overline{\overline{\mathbb{T}}}}_{{\widehat{\mathbb{H}}}_{i}}^{j}\right)}\right\},\left\{\underset{j}{\mathit{max}}{\overline{\overline{\mathbb{V}}}}_{{\widehat{\mathbb{H}}}_{r}}^{j}{e}^{i2\Pi \left(\underset{j}{\mathit{max}}{\overline{\overline{\mathbb{V}}}}_{{\widehat{\mathbb{H}}}_{a}}^{j}\right)}\right\},\left\{\underset{j}{\mathit{max}}{\overline{\overline{\mathbb{U}}}}_{{\widehat{\mathbb{H}}}_{r}}^{j}{e}^{i2\Pi \left(\underset{j}{\mathit{max}}{\overline{\overline{\mathbb{U}}}}_{{\widehat{\mathbb{H}}}_{i}}^{j}\right)}\right\}\right)$$ And $${\widehat{\mathbb{H}}}_{p}^{+}=\left(\mathit{max}{\overline{\overline{\mathbb{T}}}}_{{\widehat{\mathbb{H}}}_{p}},\mathit{min}{\overline{\overline{\mathbb{V}}}}_{{\widehat{\mathbb{H}}}_{p}},\mathit{min}{\overline{\overline{\mathbb{U}}}}_{{\widehat{\mathbb{H}}}_{p}}\right)=\left(\left\{\underset{j}{\mathit{max}}{\overline{\overline{\mathbb{T}}}}_{{\widehat{\mathbb{H}}}_{r}}^{j}{e}^{i2\Pi \left(\underset{j}{\mathit{max}}{\overline{\overline{\mathbb{T}}}}_{{\widehat{\mathbb{H}}}_{i}}^{j}\right)}\right\},\left\{\underset{j}{\mathit{min}}{\overline{\overline{\mathbb{V}}}}_{{\widehat{\mathbb{H}}}_{r}}^{j}{e}^{i2\Pi \left(\underset{j}{\mathit{min}}{\overline{\overline{\mathbb{V}}}}_{{\widehat{\mathbb{H}}}_{a}}^{j}\right)}\right\},\left\{\underset{j}{\mathit{min}}{\overline{\overline{\mathbb{U}}}}_{{\widehat{\mathbb{H}}}_{r}}^{j}{e}^{i2\Pi \left(\underset{j}{\mathit{min}}{\overline{\overline{\mathbb{U}}}}_{{\widehat{\mathbb{H}}}_{i}}^{j}\right)}\right\}\right)$$, thus

$${\widehat{\mathbb{H}}}_{p}^{-}\le CTSHFAAOWPG\left({\widehat{\mathbb{H}}}_{p}^{1},{\widehat{\mathbb{H}}}_{p}^{2},\dots ,{\widehat{\mathbb{H}}}_{p}^{n}\right)\le {\widehat{\mathbb{H}}}_{p}^{+}$$

### Proof

Straightforward. By using the above operators, our target is to simplify it with the help of some practical examples to compute the efficiency and exactness of the invented techniques.□

## Decision-making procedure based on proposed operators

In this section, we elaborate on the procedure of MADM techniques under the consideration of the proposed operators such as the CTSHFAAWPA operator and CTSHFAAWPG operator to evaluate the supremacy and validity of the derived information.

For this, we demonstrate the procedure of MADM techniques with alternatives ${\widehat{\mathbb{H}}}_{p}^{1},{\widehat{\mathbb{H}}}_{p}^{2},\dots ,{\widehat{\mathbb{H}}}_{p}^{n}$ and for each alternative, we have some attributes ${\widehat{\mathbb{H}}}_{AT}^{1},{\widehat{\mathbb{H}}}_{AT}^{2},\dots ,{\widehat{\mathbb{H}}}_{AT}^{m}$ with weight vectors ${\widehat{\Xi }}_{s}^{j}$ in the shape of power operators. Based on the above information, we compute the matrix by including the CTSHFNs, such as we observed in positive grade ${\overline{\overline{\mathbb{T}}}}_{{\widehat{\mathbb{H}}}_{p}}\left(x\right)=\left\{{\overline{\overline{\mathbb{T}}}}_{{\widehat{\mathbb{H}}}_{r}}^{j}\left(x\right){\mathcal{e}}^{i2\Pi \left({\overline{\overline{\mathbb{T}}}}_{{\widehat{\mathbb{H}}}_{i}}^{j}\left(x\right)\right)},j=\mathrm{1,2},\dots ,n\right\}$, abstinence grade ${\overline{\overline{\mathbb{V}}}}_{{\widehat{\mathbb{H}}}_{p}}\left(x\right)=\left\{{\overline{\overline{\mathbb{V}}}}_{{\widehat{\mathbb{H}}}_{r}}^{j}\left(x\right){\mathcal{e}}^{i2\Pi \left({\overline{\overline{\mathbb{V}}}}_{{\widehat{\mathbb{H}}}_{i}}^{j}\left(x\right)\right)},j=\mathrm{1,2},\dots ,n\right\}$, and a negative grade ${\overline{\overline{\mathbb{U}}}}_{{\widehat{\mathbb{H}}}_{p}}\left(x\right)=\left\{{\overline{\overline{\mathbb{U}}}}_{{\widehat{\mathbb{H}}}_{r}}^{j}\left(x\right){\mathcal{e}}^{i2\Pi \left({\overline{\overline{\mathbb{U}}}}_{i}^{j}\left(x\right)\right)},j=1,2,\dots ,n\right\}$ with the following characteristics: $\left({\left({\text{sup}}({\overline{\overline{\mathbb{T}}}}_{{\widehat{\mathbb{H}}}_{r}}^{j}\left(x\right))\right)}^{\pi }+{\left({\text{sup}}({\overline{\overline{\mathbb{V}}}}_{{\widehat{\mathbb{H}}}_{r}}^{j}\left(x\right))\right)}^{\pi }+{\left({\text{sup}}({\overline{\overline{\mathbb{U}}}}_{{\widehat{\mathbb{H}}}_{r}}^{j}\left(x\right))\right)}^{\pi },{\left({\text{sup}}({\overline{\overline{\mathbb{T}}}}_{{\widehat{\mathbb{H}}}_{i}}^{j}\left(x\right))\right)}^{\pi }+{\left({\text{sup}}{(\overline{\overline{\mathbb{V}}}}_{{\widehat{\mathbb{H}}}_{a}}^{j}\left(x\right))\right)}^{\pi }+{\left({\text{sup}}({\overline{\overline{\mathbb{U}}}}_{{\widehat{\mathbb{H}}}_{i}}^{j}\left(x\right))\right)}^{\pi }\right)\in \left[{\rm O},1\right]$, Further, we describe the main purpose of neutral grade such as: ${\overline{\overline{\mathbb{r}}}}_{{\widehat{\mathbb{H}}}_{p}}\left(x\right)=\left\{{\overline{\overline{\mathbb{r}}}}_{{\widehat{\mathbb{H}}}_{r}}^{j}\left(x\right){\mathcal{e}}^{i2\Pi \left({\overline{\overline{\mathbb{r}}}}_{{\widehat{\mathbb{H}}}_{i}}^{j}\left(x\right)\right)},j=\mathrm{1,2},\dots ,n\right\}=$

$\left\{{\left({1- (({\overline{\overline{\mathbb{T}}}}_{{\widehat{\mathbb{H}}}_{r}}^{j}\left(x\right) ) }^{\pi }+{ ({\overline{\overline{\mathbb{V}}}}_{{\widehat{\mathbb{H}}}_{r}}^{j}\left(x\right) ) }^{\pi }+{ ({\overline{\overline{\mathbb{U}}}}_{{\widehat{\mathbb{H}}}_{r}}^{j}\left(x\right) ) }^{\pi }\right)}^{\frac{1}{\pi }} {\mathcal{e}}^{{i2\Pi \left(1-\left({( {\overline{\overline{\mathbb{T}}}}_{{\widehat{\mathbb{H}}}_{i}}^{j}\left(x\right) ) }^{\pi }+({\overline{\overline{\mathbb{V}}}}_{{\widehat{\mathbb{H}}}_{a}}^{j}{\left(x\right) ) }^{\pi }+{\left({\overline{\overline{\mathbb{U}}}}_{{\widehat{\mathbb{H}}}_{i}}^{j}\left(x\right) \right)}^{\pi }\right)\right)}^{\frac{1}{\pi }} },j=\mathrm{1,2},\dots ,n\right\}$ and the purified form of the CTSHFN is deduced by : ${\widehat{\mathbb{H}}}_{p}^{j}= \left({\overline{\overline{\mathbb{T}}}}_{{\widehat{\mathbb{H}}}_{p}},{\overline{\overline{\mathbb{V}}}}_{{\widehat{\mathbb{H}}}_{p}},{\overline{\overline{\mathbb{U}}}}_{{\widehat{\mathbb{H}}}_{p}}\right)=\left(\left\{{\overline{\overline{\mathbb{T}}}}_{{\widehat{\mathbb{H}}}_{r}}^{j}{\mathcal{e}}^{i2\Pi \left({\overline{\overline{\mathbb{T}}}}_{{\widehat{\mathbb{H}}}_{i}}^{j}\right)}\right\},\left\{{\overline{\overline{\mathbb{V}}}}_{{\widehat{\mathbb{H}}}_{r}}^{j}{\mathcal{e}}^{i2\Pi \left({\overline{\overline{\mathbb{V}}}}_{{\widehat{\mathbb{H}}}_{a}}^{j}\right)}\right\},\left\{{\overline{\overline{\mathbb{U}}}}_{{\widehat{\mathbb{H}}}_{r}}^{j}{\mathcal{e}}^{i2\Pi \left({\overline{\overline{\mathbb{U}}}}_{{\widehat{\mathbb{H}}}_{i}}^{j}\right)}\right\}\right),j=\mathrm{1,2},\dots ,n.$ Using the above information, we compute the procedure of MADM techniques to evaluate the above problems, such as.

Step 1: Derive the matrix by putting the values of CTSHFNs, if we have cost type of data, then

$$Z=\left\{\begin{array}{cc}\left(\left\{{\overline{\overline{\mathbb{T}}}}_{{\widehat{\mathbb{H}}}_{r}}^{j}{\mathcal{e}}^{i2\Pi \left({\overline{\overline{\mathbb{T}}}}_{{\widehat{\mathbb{H}}}_{i}}^{j}\right)}\right\},\left\{{\overline{\overline{\mathbb{V}}}}_{{\widehat{\mathbb{H}}}_{r}}^{j}{\mathcal{e}}^{i2\Pi \left({\overline{\overline{\mathbb{V}}}}_{{\widehat{\mathbb{H}}}_{a}}^{j}\right)}\right\},\left\{{\overline{\overline{\mathbb{U}}}}_{{\widehat{\mathbb{H}}}_{r}}^{j}{\mathcal{e}}^{i2\Pi \left({\overline{\overline{\mathbb{U}}}}_{{\widehat{\mathbb{H}}}_{i}}^{j}\right)}\right\}\right)& for benefit\\ \left(\left\{{\overline{\overline{\mathbb{U}}}}_{{\widehat{\mathbb{H}}}_{r}}^{j}{\mathcal{e}}^{i2\Pi \left({\overline{\overline{\mathbb{U}}}}_{{\widehat{\mathbb{H}}}_{i}}^{j}\right)}\right\},\left\{{\overline{\overline{\mathbb{V}}}}_{{\widehat{\mathbb{H}}}_{r}}^{j}{\mathcal{e}}^{i2\Pi \left({\overline{\overline{\mathbb{V}}}}_{{\widehat{\mathbb{H}}}_{a}}^{j}\right)}\right\},\left\{{\overline{\overline{\mathbb{T}}}}_{{\widehat{\mathbb{H}}}_{r}}^{j}{\mathcal{e}}^{i2\Pi \left({\overline{\overline{\mathbb{T}}}}_{{\widehat{\mathbb{H}}}_{i}}^{j}\right)}\right\}\right)& for cost\end{array}\right.$$

But if we have a benefit type of data, then do not normalize the matrix.

Step 2: Aggregate the matrix based on the CTSHFAAWPA operator and CTSHFAAWPG operator, such as

$$CTSHFAAWPPA\left({\widehat{\mathbb{H}}}_{p}^{1},{\widehat{\mathbb{H}}}_{p}^{2},\dots ,{\widehat{\mathbb{H}}}_{p}^{n}\right)=\coprod_{\left(\begin{array}{c}{\overline{\overline{\mathbb{T}}}}_{{\widehat{\mathbb{H}}}_{r}}^{j},{\overline{\overline{\mathbb{T}}}}_{{\widehat{\mathbb{H}}}_{i}}^{j}\in {\overline{\overline{\mathbb{T}}}}_{{\widehat{\mathbb{H}}}_{\begin{array}{c}p\end{array}}}\\ {\overline{\overline{\mathbb{V}}}}_{{\widehat{\mathbb{H}}}_{r}}^{j},{\overline{\overline{\mathbb{V}}}}_{{\widehat{\mathbb{H}}}_{a}}^{j}\in {\overline{\overline{\mathbb{V}}}}_{{\widehat{\mathbb{H}}}_{\begin{array}{c}p\end{array}}}\\ {\overline{\overline{\mathbb{U}}}}_{{\widehat{\mathbb{H}}}_{r}}^{j},{\overline{\overline{\mathbb{U}}}}_{{\widehat{\mathbb{H}}}_{i}}^{j}\in {\overline{\overline{\mathbb{U}}}}_{{\widehat{\mathbb{H}}}_{\begin{array}{c}p\end{array}}}\end{array}\right)}\left(\begin{array}{c}\genfrac{}{}{0pt}{}{\begin{array}{c}{\left(1-{\mathcal{e}}^{-{\left({\sum }_{j=1}^{n}{{\widehat{\Xi }}_{s}^{j}\left(-{\text{ln}}\left(1-{\left({\overline{\overline{\mathbb{T}}}}_{{\widehat{\mathbb{H}}}_{r}}^{j}\right)}^{\pi }\right)\right)}^{\varnothing } \right)}^{\frac{1}{\varnothing }}}\right)}^{\frac{1}{\pi }}{\mathcal{e}}^{i2\Pi {\left(1-{\mathcal{e}}^{-{\left({\sum }_{j=1}^{n}{{\widehat{\Xi }}_{s}^{j}\left(-{\text{ln}}\left(1-{\left({\overline{\overline{\mathbb{T}}}}_{{\widehat{\mathbb{H}}}_{i}}^{j}\right)}^{\pi }\right)\right)}^{\varnothing } \right)}^{\frac{1}{\varnothing }}}\right)}^{\frac{1}{\pi }}},\\ {\left({\mathcal{e}}^{-{\left({\sum }_{j=1}^{n}{{\widehat{\Xi }}_{s}^{j}\left(-{\text{ln}}\left(1-{\left({\overline{\overline{\mathbb{V}}}}_{{\widehat{\mathbb{H}}}_{r}}^{j}\right)}^{\pi }\right)\right)}^{\varnothing } \right)}^{\frac{1}{\varnothing }}}\right)}^{\frac{1}{\pi }} {\mathcal{e}}^{i2\Pi {\left({\mathcal{e}}^{-{\left({\sum }_{j=1}^{n}{{\widehat{\Xi }}_{s}^{j}\left(-{\text{ln}}\left(1-{\left({\overline{\overline{\mathbb{V}}}}_{{\widehat{\mathbb{H}}}_{a}}^{j}\right)}^{\pi }\right)\right)}^{\varnothing } \right)}^{\frac{1}{\varnothing }}}\right)}^{\frac{1}{\pi }}},\end{array}}{{\left({\mathcal{e}}^{-{\left({\sum }_{j=1}^{n}{{\widehat{\Xi }}_{s}^{j}\left(-{\text{ln}}\left(1-{\left({\overline{\overline{\mathbb{U}}}}_{{\widehat{\mathbb{H}}}_{r}}^{j}\right)}^{\pi }\right)\right)}^{\varnothing } \right)}^{\frac{1}{\varnothing }}}\right)}^{\frac{1}{\pi }} {\mathcal{e}}^{i2\Pi {\left({\mathcal{e}}^{-{\left({\sum }_{j=1}^{n}{{\widehat{\Xi }}_{s}^{j}\left(-{\text{ln}}\left(1-{\left({\overline{\overline{\mathbb{U}}}}_{{\widehat{\mathbb{H}}}_{i}}^{j}\right)}^{\pi }\right)\right)}^{\varnothing } \right)}^{\frac{1}{\varnothing }}}\right)}^{\frac{1}{\pi }}}}\end{array}\right)$$

$$CTSHFAAWPG\left({\widehat{\mathbb{H}}}_{p}^{1},{\widehat{\mathbb{H}}}_{p}^{2},\dots ,{\widehat{\mathbb{H}}}_{p}^{n}\right)=\coprod_{\left(\begin{array}{c}{\overline{\overline{\mathbb{T}}}}_{{\widehat{\mathbb{H}}}_{r}}^{j},{\overline{\overline{\mathbb{T}}}}_{{\widehat{\mathbb{H}}}_{i}}^{j}\in {\overline{\overline{\mathbb{T}}}}_{{\widehat{\mathbb{H}}}_{\begin{array}{c}p\end{array}}}\\ {\overline{\overline{\mathbb{V}}}}_{{\widehat{\mathbb{H}}}_{r}}^{j},{\overline{\overline{\mathbb{V}}}}_{{\widehat{\mathbb{H}}}_{a}}^{j}\in {\overline{\overline{\mathbb{V}}}}_{{\widehat{\mathbb{H}}}_{\begin{array}{c}p\end{array}}}\\ {\overline{\overline{\mathbb{U}}}}_{{\widehat{\mathbb{H}}}_{r}}^{j},{\overline{\overline{\mathbb{U}}}}_{{\widehat{\mathbb{H}}}_{i}}^{j}\in {\overline{\overline{\mathbb{U}}}}_{{\widehat{\mathbb{H}}}_{\begin{array}{c}p\end{array}}}\end{array}\right)}\begin{array}{c}\left(\begin{array}{c}\genfrac{}{}{0pt}{}{\begin{array}{c}{\left({\mathcal{e}}^{-{\left({\sum }_{j=1}^{n}{{\widehat{\Xi }}_{s}^{j}\left(-{\text{ln}}\left({\left({\overline{\overline{\mathbb{T}}}}_{{\widehat{\mathbb{H}}}_{r}}^{j}\right)}^{\pi }\right)\right)}^{\varnothing } \right)}^{\frac{1}{\varnothing }}}\right)}^{\frac{1}{\pi }}{\mathcal{e}}^{i2\Pi {\left({\mathcal{e}}^{-{\left({\sum }_{j=1}^{n}{{\widehat{\Xi }}_{s}^{j}\left(-{\text{ln}}\left({\left({\overline{\overline{\mathbb{T}}}}_{{\widehat{\mathbb{H}}}_{i}}^{j}\right)}^{\pi }\right)\right)}^{\varnothing } \right)}^{\frac{1}{\varnothing }}}\right)}^{\frac{1}{\pi }}},\\ {\left(1-{\mathcal{e}}^{-{\left({\sum }_{j=1}^{n}{{\widehat{\Xi }}_{s}^{j}\left(-{\text{ln}}\left(1-{\left({\overline{\overline{\mathbb{V}}}}_{{\widehat{\mathbb{H}}}_{r}}^{j}\right)}^{\pi }\right)\right)}^{\varnothing } \right)}^{\frac{1}{\varnothing }}}\right)}^{\frac{1}{\pi }} {\mathcal{e}}^{i2\Pi {\left(1-{\mathcal{e}}^{-{\left({\sum }_{j=1}^{n}{{\widehat{\Xi }}_{s}^{j}\left(-{\text{ln}}\left(1-{\left({\overline{\overline{\mathbb{V}}}}_{{\widehat{\mathbb{H}}}_{a}}^{j}\right)}^{\pi }\right)\right)}^{\varnothing } \right)}^{\frac{1}{\varnothing }}}\right)}^{\frac{1}{\pi }}},\end{array}}{{\left({\mathcal{e}}^{-{\left({\sum }_{j=1}^{k}{{\widehat{\Xi }}_{s}^{j}\left(-{\text{ln}}\left(1-{\left({\overline{\overline{\mathbb{U}}}}_{{\widehat{\mathbb{H}}}_{r}}^{j}\right)}^{\pi }\right)\right)}^{\varnothing } \right)}^{\frac{1}{\varnothing }}}\right)}^{\frac{1}{\pi }} {\mathcal{e}}^{i2\Pi {\left(1-{\mathcal{e}}^{-{\left({\sum }_{j=1}^{n}{{\widehat{\Xi }}_{s}^{j}\left(-{\text{ln}}\left(1-{\left({\overline{\overline{\mathbb{U}}}}_{{\widehat{\mathbb{H}}}_{i}}^{j}\right)}^{\pi }\right)\right)}^{\varnothing } \right)}^{\frac{1}{\varnothing }}}\right)}^{\frac{1}{\pi }}}}\end{array}\right)\\ \end{array}$$

Step 3: Evaluate the Score values of the above-aggregated information, such as

$${\overline{\overline{\mathrm{^\circ{\rm F} }}}}_{{\text{SV}}}\left({\widehat{\mathbb{H}}}_{p}^{1}\right)=\frac{1}{6}\left(\frac{1}{n}\sum_{j=1}^{n}{\overline{\overline{t}}}_{{\widehat{\mathbb{H}}}_{r}}^{j}+\frac{1}{n}\sum_{j=1}^{n}{\overline{\overline{t}}}_{{\widehat{\mathbb{H}}}_{i}}^{j}-\frac{1}{n}\sum_{j=1}^{n}{\overline{\overline{h}}}_{{\widehat{\mathbb{H}}}_{r}}^{j}-\frac{1}{n}\sum_{j=1}^{n}{\overline{\overline{h}}}_{{\widehat{\mathbb{H}}}_{a}}^{j}-\frac{1}{n}\sum_{j=1}^{n}{\overline{\overline{f}}}_{{\widehat{\mathbb{H}}}_{r}}^{j}-\frac{1}{n}\sum_{j=1}^{n}{\overline{\overline{f}}}_{{\widehat{\mathbb{H}}}_{i}}^{j}\right) \epsilon \left[-\mathrm{1,1}\right]$$

$${\overline{\overline{\mathrm{^\circ{\rm F} }}}}_{{\text{AV}}}\left({\widehat{\mathbb{H}}}_{p}^{1}\right)=\frac{1}{6}\left(\frac{1}{n}\sum_{j=1}^{n}{\overline{\overline{t}}}_{{\widehat{\mathbb{H}}}_{r}}^{j}+\frac{1}{n}\sum_{j=1}^{n}{\overline{\overline{t}}}_{{\widehat{\mathbb{H}}}_{i}}^{j}+\frac{1}{n}\sum_{j=1}^{n}{\overline{\overline{h}}}_{{\widehat{\mathbb{H}}}_{r}}^{j}+\frac{1}{n}\sum_{j=1}^{n}{\overline{\overline{h}}}_{{\widehat{\mathbb{H}}}_{a}}^{j}+\frac{1}{n}\sum_{j=1}^{n}{\overline{\overline{f}}}_{{\widehat{\mathbb{H}}}_{r}}^{j}+\frac{1}{n}\sum_{j=1}^{n}{\overline{\overline{f}}}_{{\widehat{\mathbb{H}}}_{i}}^{j}\right) \epsilon \left[{\rm O},1\right]$$

**Figure 2:**
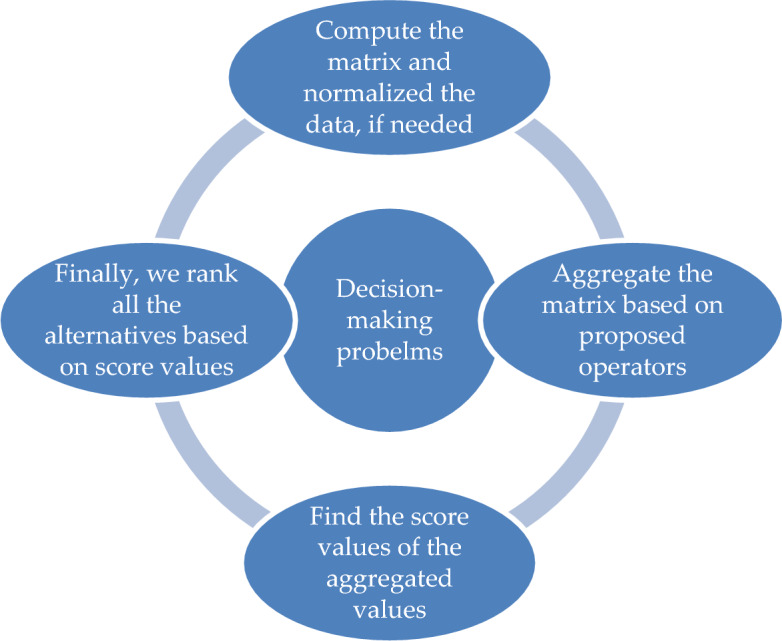
The geometrical shape of the proposed algorithm.

Step 4: Derive the ranking values according to the score function to examine the best one. The geometrical shape of the proposed algorithm is listed in the shape of Fig. 2.

To simplify some real-life problems, we use the above procedure based on the MADM technique to improve the reliability and supremacy of the proposed information.

### Application of 3D Seismic Attributes analysis to Mine Planning Based on Proposed Methods

In this application, we evaluate the best way in which 3D seismic attribute analysis can be used or applied to mine planning. 3D seismic attributes analysis can help geologists and mine developers associate subsurface geological features, structures, faults, and ore bodies more precisely and accurately. The major influence of this application is to evaluate the usage of the 3D seismic attributes analysis in mine planning. For this, we consider some ways which will be used as an alternative, such as

1. Geological Mapping and Target Identification “${\widehat{\mathbb{H}}}_{p}^{1}$”.
2. Structure Analysis “$${\widehat{\mathbb{H}}}_{p}^{2}$$”.
3. Reserves Estimation “$${\widehat{\mathbb{H}}}_{p}^{3}$$”.
4. Mine Design Optimization “${\widehat{\mathbb{H}}}_{p}^{4}$”.
5. Health and Safety “${\widehat{\mathbb{H}}}_{p}^{5}$”.

To depict the best one, we use the following features such as growth analysis, social impact, political impact, and environmental impact. Using the above information, we compute the procedure of MADM techniques to evaluate the above problems, such as

Step 1: Derive the matrix by putting the values of CTSHFNs, see Table 1, if we have cost type of data, then

**Table 1:** CTSHF decision-matrix.

	${\widehat{{\varvec{\eta}}}}_{{\varvec{p}}}^{1}$	${\widehat{{\varvec{\eta}}}}_{{\varvec{p}}}^{2}$
${\widehat{{\varvec{\eta}}}}_{{\varvec{p}}}^{{\varvec{A}}-1}$	$$\left(\begin{array}{c}\left\{{\rm O}.2{\mathcal{e}}^{i2\Pi \left({\rm O}.3\right)},{\rm O}.4{\mathcal{e}}^{i2\Pi \left({\rm O}.7\right)},{\rm O}.7{\mathcal{e}}^{i2\Pi \left({\rm O}.8\right)}\right\},\\ \left\{{\rm O}.2{\mathcal{e}}^{i2\Pi \left({\rm O}.3\right)}\right\},\\ \left\{{\rm O}.6{\mathcal{e}}^{i2\Pi \left({\rm O}.5\right)},{\rm O}.7{\mathcal{e}}^{i2\Pi \left({\rm O}.5\right)}\right\}\end{array}\right)$$	$$\left(\begin{array}{c}\left\{{\rm O}.21{\mathcal{e}}^{i2\Pi \left({\rm O}.31\right)},{\rm O}.41{\mathcal{e}}^{i2\Pi \left({\rm O}.71\right)},{\rm O}.71{\mathcal{e}}^{i2\Pi \left({\rm O}.81\right)}\right\},\\ \left\{{\rm O}.21{\mathcal{e}}^{i2\Pi \left({\rm O}.31\right)}\right\},\\ \left\{{\rm O}.6{1\mathcal{e}}^{i2\Pi \left({\rm O}.51\right)},{\rm O}.71{\mathcal{e}}^{i2\Pi \left({\rm O}.51\right)}\right\}\end{array}\right)$$
${\widehat{{\varvec{\eta}}}}_{{\varvec{p}}}^{{\varvec{A}}-2}$	$$\left(\begin{array}{c}\left\{{\rm O}.1{\mathcal{e}}^{i2\Pi \left({\rm O}.7\right)},{\rm O}.3{\mathcal{e}}^{i2\Pi \left({\rm O}.5\right)},{\rm O}.6{\mathcal{e}}^{i2\Pi \left({\rm O}.8\right)}\right\},\\ \left\{{\rm O}.4{\mathcal{e}}^{i2\Pi \left({\rm O}.2\right)}\right\},\\ \left\{{\rm O}.3{\mathcal{e}}^{i2\Pi \left({\rm O}.4\right)},{\rm O}.3{\mathcal{e}}^{i2\Pi \left({\rm O}.1\right)}\right\}\end{array}\right)$$	$$\left(\begin{array}{c}\left\{{\rm O}.11{\mathcal{e}}^{i2\Pi \left({\rm O}.71\right)},{\rm O}.31{\mathcal{e}}^{i2\Pi \left({\rm O}.51\right)},{\rm O}.61{\mathcal{e}}^{i2\Pi \left({\rm O}.81\right)}\right\},\\ \left\{{\rm O}.41{\mathcal{e}}^{i2\Pi \left({\rm O}.21\right)}\right\},\\ \left\{{\rm O}.31{\mathcal{e}}^{i2\Pi \left({\rm O}.41\right)},{\rm O}.31{\mathcal{e}}^{i2\Pi \left({\rm O}.11\right)}\right\}\end{array}\right)$$
${\widehat{{\varvec{\eta}}}}_{{\varvec{p}}}^{{\varvec{A}}-3}$	$$\left(\begin{array}{c}\left\{{\rm O}.2{\mathcal{e}}^{i2\Pi \left({\rm O}.3\right)},{\rm O}.1{\mathcal{e}}^{i2\Pi \left({\rm O}.4\right)},{\rm O}.8{\mathcal{e}}^{i2\Pi \left({\rm O}.5\right)}\right\},\\ \left\{{\rm O}.3{\mathcal{e}}^{i2\Pi \left({\rm O}.5\right)}\right\},\\ \left\{{\rm O}.4{\mathcal{e}}^{i2\Pi \left({\rm O}.3\right)},{\rm O}.7{\mathcal{e}}^{i2\Pi \left({\rm O}.7\right)}\right\}\end{array}\right)$$	$$\left(\begin{array}{c}\left\{{\rm O}.21{\mathcal{e}}^{i2\Pi \left({\rm O}.31\right)},{\rm O}.11{\mathcal{e}}^{i2\Pi \left({\rm O}.41\right)},{\rm O}.81{\mathcal{e}}^{i2\Pi \left({\rm O}.51\right)}\right\},\\ \left\{{\rm O}.3{1\mathcal{e}}^{i2\Pi \left({\rm O}.51\right)}\right\},\\ \left\{{\rm O}.4{1\mathcal{e}}^{i2\Pi \left({\rm O}.31\right)},{\rm O}.71{\mathcal{e}}^{i2\Pi \left({\rm O}.71\right)}\right\}\end{array}\right)$$
${\widehat{{\varvec{\eta}}}}_{{\varvec{p}}}^{{\varvec{A}}-4}$	$$\left(\begin{array}{c}\left\{{\rm O}.1{\mathcal{e}}^{i2\Pi \left({\rm O}.5\right)},{\rm O}.6{\mathcal{e}}^{i2\Pi \left({\rm O}.3\right)},{\rm O}.6{\mathcal{e}}^{i2\Pi \left({\rm O}.8\right)}\right\},\\ \left\{{\rm O}.7{\mathcal{e}}^{i2\Pi \left({\rm O}.3\right)}\right\},\\ \left\{{\rm O}.8{\mathcal{e}}^{i2\Pi \left({\rm O}.6\right)},{\rm O}.9{\mathcal{e}}^{i2\Pi \left({\rm O}.3\right)}\right\}\end{array}\right)$$	$$\left(\begin{array}{c}\left\{{\rm O}.11{\mathcal{e}}^{i2\Pi \left({\rm O}.51\right)},{\rm O}.61{\mathcal{e}}^{i2\Pi \left({\rm O}.31\right)},{\rm O}.6{1\mathcal{e}}^{i2\Pi \left({\rm O}.81\right)}\right\},\\ \left\{{\rm O}.71{\mathcal{e}}^{i2\Pi \left({\rm O}.31\right)}\right\},\\ \left\{{\rm O}.8{1\mathcal{e}}^{i2\Pi \left({\rm O}.61\right)},{\rm O}.91{\mathcal{e}}^{i2\Pi \left({\rm O}.31\right)}\right\}\end{array}\right)$$
${\widehat{{\varvec{\eta}}}}_{{\varvec{p}}}^{{\varvec{A}}-5}$	$$\left(\begin{array}{c}\left\{{\rm O}.5{\mathcal{e}}^{i2\Pi \left({\rm O}.6\right)},{\rm O}.3{\mathcal{e}}^{i2\Pi \left({\rm O}.4\right)},{\rm O}.2{\mathcal{e}}^{i2\Pi \left({\rm O}.9\right)}\right\},\\ \left\{{\rm O}.5{\mathcal{e}}^{i2\Pi \left({\rm O}.4\right)}\right\},\\ \left\{{\rm O}.9{\mathcal{e}}^{i2\Pi \left({\rm O}.3\right)},{\rm O}.5{\mathcal{e}}^{i2\Pi \left({\rm O}.4\right)}\right\}\end{array}\right)$$	$$\left(\begin{array}{c}\left\{{\rm O}.51{\mathcal{e}}^{i2\Pi \left({\rm O}.61\right)},{\rm O}.3{1\mathcal{e}}^{i2\Pi \left({\rm O}.41\right)},{\rm O}.21{\mathcal{e}}^{i2\Pi \left({\rm O}.91\right)}\right\},\\ \left\{{\rm O}.51{\mathcal{e}}^{i2\Pi \left({\rm O}.41\right)}\right\},\\ \left\{{\rm O}.91{\mathcal{e}}^{i2\Pi \left({\rm O}.31\right)},{\rm O}.5{1\mathcal{e}}^{i2\Pi \left({\rm O}.41\right)}\right\}\end{array}\right)$$
	${\widehat{{\varvec{\eta}}}}_{{\varvec{p}}}^{3}$	${\widehat{{\varvec{\eta}}}}_{{\varvec{p}}}^{4}$
${\widehat{{\varvec{\eta}}}}_{{\varvec{p}}}^{{\varvec{A}}-1}$	$$\left(\begin{array}{c}\left\{{\rm O}.22{\mathcal{e}}^{i2\Pi \left({\rm O}.32\right)},{\rm O}.42{\mathcal{e}}^{i2\Pi \left({\rm O}.72\right)},{\rm O}.72{\mathcal{e}}^{i2\Pi \left({\rm O}.82\right)}\right\},\\ \left\{{\rm O}.22{\mathcal{e}}^{i2\Pi \left({\rm O}.32\right)}\right\},\\ \left\{{\rm O}.6{2\mathcal{e}}^{i2\Pi \left({\rm O}.52\right)},{\rm O}.72{\mathcal{e}}^{i2\Pi \left({\rm O}.52\right)}\right\}\end{array}\right)$$	$$\left(\begin{array}{c}\left\{{\rm O}.23{\mathcal{e}}^{i2\Pi \left({\rm O}.33\right)},{\rm O}.43{\mathcal{e}}^{i2\Pi \left({\rm O}.73\right)},{\rm O}.73{\mathcal{e}}^{i2\Pi \left({\rm O}.83\right)}\right\},\\ \left\{{\rm O}.23{\mathcal{e}}^{i2\Pi \left({\rm O}.33\right)}\right\},\\ \left\{{\rm O}.6{3\mathcal{e}}^{i2\Pi \left({\rm O}.53\right)},{\rm O}.73{\mathcal{e}}^{i2\Pi \left({\rm O}.53\right)}\right\}\end{array}\right)$$
${\widehat{{\varvec{\eta}}}}_{{\varvec{p}}}^{{\varvec{A}}-2}$	$$\left(\begin{array}{c}\left\{{\rm O}.12{\mathcal{e}}^{i2\Pi \left({\rm O}.72\right)},{\rm O}.32{\mathcal{e}}^{i2\Pi \left({\rm O}.52\right)},{\rm O}.62{\mathcal{e}}^{i2\Pi \left({\rm O}.82\right)}\right\},\\ \left\{{\rm O}.42{\mathcal{e}}^{i2\Pi \left({\rm O}.22\right)}\right\},\\ \left\{{\rm O}.32{\mathcal{e}}^{i2\Pi \left({\rm O}.42\right)},{\rm O}.32{\mathcal{e}}^{i2\Pi \left({\rm O}.12\right)}\right\}\end{array}\right)$$	$$\left(\begin{array}{c}\left\{{\rm O}.13{\mathcal{e}}^{i2\Pi \left({\rm O}.73\right)},{\rm O}.33{\mathcal{e}}^{i2\Pi \left({\rm O}.53\right)},{\rm O}.63{\mathcal{e}}^{i2\Pi \left({\rm O}.83\right)}\right\},\\ \left\{{\rm O}.43{\mathcal{e}}^{i2\Pi \left({\rm O}.23\right)}\right\},\\ \left\{{\rm O}.33{\mathcal{e}}^{i2\Pi \left({\rm O}.43\right)},{\rm O}.33{\mathcal{e}}^{i2\Pi \left({\rm O}.13\right)}\right\}\end{array}\right)$$
${\widehat{{\varvec{\eta}}}}_{{\varvec{p}}}^{{\varvec{A}}-3}$	$$\left(\begin{array}{c}\left\{{\rm O}.22{\mathcal{e}}^{i2\Pi \left({\rm O}.32\right)},{\rm O}.12{\mathcal{e}}^{i2\Pi \left({\rm O}.42\right)},{\rm O}.82{\mathcal{e}}^{i2\Pi \left({\rm O}.52\right)}\right\},\\ \left\{{\rm O}.3{2\mathcal{e}}^{i2\Pi \left({\rm O}.52\right)}\right\},\\ \left\{{\rm O}.4{2\mathcal{e}}^{i2\Pi \left({\rm O}.32\right)},{\rm O}.72{\mathcal{e}}^{i2\Pi \left({\rm O}.72\right)}\right\}\end{array}\right)$$	$$\left(\begin{array}{c}\left\{{\rm O}.23{\mathcal{e}}^{i2\Pi \left({\rm O}.33\right)},{\rm O}.13{\mathcal{e}}^{i2\Pi \left({\rm O}.43\right)},{\rm O}.83{\mathcal{e}}^{i2\Pi \left({\rm O}.53\right)}\right\},\\ \left\{{\rm O}.3{3\mathcal{e}}^{i2\Pi \left({\rm O}.53\right)}\right\},\\ \left\{{\rm O}.4{3\mathcal{e}}^{i2\Pi \left({\rm O}.33\right)},{\rm O}.73{\mathcal{e}}^{i2\Pi \left({\rm O}.73\right)}\right\}\end{array}\right)$$
${\widehat{{\varvec{\eta}}}}_{{\varvec{p}}}^{{\varvec{A}}-4}$	$$\left(\begin{array}{c}\left\{{\rm O}.12{\mathcal{e}}^{i2\Pi \left({\rm O}.52\right)},{\rm O}.62{\mathcal{e}}^{i2\Pi \left({\rm O}.32\right)},{\rm O}.6{2\mathcal{e}}^{i2\Pi \left({\rm O}.82\right)}\right\},\\ \left\{{\rm O}.72{\mathcal{e}}^{i2\Pi \left({\rm O}.32\right)}\right\},\\ \left\{{\rm O}.8{2\mathcal{e}}^{i2\Pi \left({\rm O}.62\right)},{\rm O}.92{\mathcal{e}}^{i2\Pi \left({\rm O}.32\right)}\right\}\end{array}\right)$$	$$\left(\begin{array}{c}\left\{{\rm O}.13{\mathcal{e}}^{i2\Pi \left({\rm O}.53\right)},{\rm O}.63{\mathcal{e}}^{i2\Pi \left({\rm O}.33\right)},{\rm O}.6{3\mathcal{e}}^{i2\Pi \left({\rm O}.83\right)}\right\},\\ \left\{{\rm O}.73{\mathcal{e}}^{i2\Pi \left({\rm O}.33\right)}\right\},\\ \left\{{\rm O}.8{3\mathcal{e}}^{i2\Pi \left({\rm O}.63\right)},{\rm O}.93{\mathcal{e}}^{i2\Pi \left({\rm O}.33\right)}\right\}\end{array}\right)$$
${\widehat{{\varvec{\eta}}}}_{{\varvec{p}}}^{{\varvec{A}}-5}$	$$\left(\begin{array}{c}\left\{{\rm O}.52{\mathcal{e}}^{i2\Pi \left({\rm O}.62\right)},{\rm O}.3{2\mathcal{e}}^{i2\Pi \left({\rm O}.42\right)},{\rm O}.22{\mathcal{e}}^{i2\Pi \left({\rm O}.92\right)}\right\},\\ \left\{{\rm O}.52{\mathcal{e}}^{i2\Pi \left({\rm O}.42\right)}\right\},\\ \left\{{\rm O}.92{\mathcal{e}}^{i2\Pi \left({\rm O}.32\right)},{\rm O}.5{2\mathcal{e}}^{i2\Pi \left({\rm O}.42\right)}\right\}\end{array}\right)$$	$$\left(\begin{array}{c}\left\{{\rm O}.53{\mathcal{e}}^{i2\Pi \left({\rm O}.63\right)},{\rm O}.3{3\mathcal{e}}^{i2\Pi \left({\rm O}.43\right)},{\rm O}.23{\mathcal{e}}^{i2\Pi \left({\rm O}.93\right)}\right\},\\ \left\{{\rm O}.53{\mathcal{e}}^{i2\Pi \left({\rm O}.43\right)}\right\},\\ \left\{{\rm O}.93{\mathcal{e}}^{i2\Pi \left({\rm O}.33\right)},{\rm O}.5{3\mathcal{e}}^{i2\Pi \left({\rm O}.43\right)}\right\}\end{array}\right)$$

$$Z=\left\{\begin{array}{cc}\left(\left\{{\overline{\overline{\mathbb{T}}}}_{{\widehat{\mathbb{H}}}_{r}}^{j}{\mathcal{e}}^{i2\Pi \left({\overline{\overline{\mathbb{T}}}}_{{\widehat{\mathbb{H}}}_{i}}^{j}\right)}\right\},\left\{{\overline{\overline{\mathbb{V}}}}_{{\widehat{\mathbb{H}}}_{r}}^{j}{\mathcal{e}}^{i2\Pi \left({\overline{\overline{\mathbb{V}}}}_{{\widehat{\mathbb{H}}}_{a}}^{j}\right)}\right\},\left\{{\overline{\overline{\mathbb{U}}}}_{{\widehat{\mathbb{H}}}_{r}}^{j}{\mathcal{e}}^{i2\Pi \left({\overline{\overline{\mathbb{U}}}}_{{\widehat{\mathbb{H}}}_{i}}^{j}\right)}\right\}\right)& for benefit\\ \left(\left\{{\overline{\overline{\mathbb{U}}}}_{{\widehat{\mathbb{H}}}_{r}}^{j}{\mathcal{e}}^{i2\Pi \left({\overline{\overline{\mathbb{U}}}}_{{\widehat{\mathbb{H}}}_{i}}^{j}\right)}\right\},\left\{{\overline{\overline{\mathbb{V}}}}_{{\widehat{\mathbb{H}}}_{r}}^{j}{\mathcal{e}}^{i2\Pi \left({\overline{\overline{\mathbb{V}}}}_{{\widehat{\mathbb{H}}}_{a}}^{j}\right)}\right\},\left\{{\overline{\overline{\mathbb{T}}}}_{{\widehat{\mathbb{H}}}_{r}}^{j}{\mathcal{e}}^{i2\Pi \left({\overline{\overline{\mathbb{T}}}}_{{\widehat{\mathbb{H}}}_{i}}^{j}\right)}\right\}\right)& for cost\end{array}\right.$$

But if we have benefit type of data, then do not normalize the matrix., however, the data in Table 1 is not required to be evaluated.

Step 2: Aggregate the matrix based on the CTSHFAAWPA operator and CTSHFAAWPG operator, see Table 2 for $\varnothing =2$.

**Table 2:** CTSHFAAPO aggregated matrix.

	**CTSHFAAPAWA operator**	**CTSHFAAPAWG operator**
${\widehat{{\varvec{\eta}}}}_{{\varvec{p}}}^{{\varvec{A}}-1}$	$$\left(\begin{array}{c}\left\{\begin{array}{c}{\rm O}.2331{\mathcal{e}}^{i2\Pi \left({\rm O}.3394\right)},{\rm O}.4462{\mathcal{e}}^{i2\Pi \left({\rm O}.7658\right)},\\ {\rm O}.7658{\mathcal{e}}^{i2\Pi \left({\rm O}.8684\right)}\end{array}\right\},\\ \left\{{\rm O}.{\rm O}461{\mathcal{e}}^{i2\Pi \left({\rm O}.{\rm O}991\right)}\right\},\\ \left\{{\rm O}.3782{\mathcal{e}}^{i2\Pi \left({\rm O}.2651\right)},{\rm O}.5111{\mathcal{e}}^{i2\Pi \left({\rm O}.2651\right)}\right\}\end{array}\right)$$	$$\left(\begin{array}{c}\left\{\begin{array}{c}{\rm O}.0461{\mathcal{e}}^{i2\Pi \left({\rm O}.0991\right)},{\rm O}.1721{\mathcal{e}}^{i2\Pi \left({\rm O}.5111\right)},\\ {\rm O}.5111{\mathcal{e}}^{i2\Pi \left({\rm O}.6641\right)}\end{array}\right\},\\ \left\{{\rm O}.2331{\mathcal{e}}^{i2\Pi \left({\rm O}.3394\right)}\right\},\\ \left\{{\rm O}.6598{\mathcal{e}}^{i2\Pi \left({\rm O}.5530\right)},{\rm O}.7658{\mathcal{e}}^{i2\Pi \left({\rm O}.5530\right)}\right\}\end{array}\right)$$
${\widehat{{\varvec{\eta}}}}_{{\varvec{p}}}^{{\varvec{A}}-2}$	$$\left(\begin{array}{c}\left\{\begin{array}{c}{\rm O}.1279{\mathcal{e}}^{i2\Pi \left({\rm O}.7658\right)},{\rm O}.3394{\mathcal{e}}^{i2\Pi \left({\rm O}.5530\right)},\\ {\rm O}.6598{\mathcal{e}}^{i2\Pi \left({\rm O}.8684\right)}\end{array}\right\},\\ \left\{{\rm O}.1721{\mathcal{e}}^{i2\Pi \left({\rm O}.{\rm O}461\right)}\right\},\\ \left\{{\rm O}.0991{\mathcal{e}}^{i2\Pi \left({\rm O}.1721\right)},{\rm O}.0991{\mathcal{e}}^{i2\Pi \left({\rm O}.{\rm O}131\right)}\right\}\end{array}\right)$$	$$\left(\begin{array}{c}\left\{\begin{array}{c}{\rm O}.0131{\mathcal{e}}^{i2\Pi \left({\rm O}.5111\right)},{\rm O}.0991{\mathcal{e}}^{i2\Pi \left({\rm O}.2651\right)},\\ {\rm O}.3781{\mathcal{e}}^{i2\Pi \left({\rm O}.6641\right)}\end{array}\right\},\\ \left\{{\rm O}.4462{\mathcal{e}}^{i2\Pi \left({\rm O}.2331\right)}\right\},\\ \left\{{\rm O}.3394{\mathcal{e}}^{i2\Pi \left({\rm O}.4462\right)},{\rm O}.3394{\mathcal{e}}^{i2\Pi \left({\rm O}.1279\right)}\right\}\end{array}\right)$$
${\widehat{{\varvec{\eta}}}}_{{\varvec{p}}}^{{\varvec{A}}-3}$	$$\left(\begin{array}{c}\left\{\begin{array}{c}{\rm O}.2331{\mathcal{e}}^{i2\Pi \left({\rm O}.3394\right)},{\rm O}.1279{\mathcal{e}}^{i2\Pi \left({\rm O}.4462\right)},\\ {\rm O}.8684{\mathcal{e}}^{i2\Pi \left({\rm O}.5530\right)}\end{array}\right\},\\ \left\{{\rm O}.{\rm O}991{\mathcal{e}}^{i2\Pi \left({\rm O}.2651\right)}\right\},\\ \left\{{\rm O}.1721{\mathcal{e}}^{i2\Pi \left({\rm O}.0991\right)},{\rm O}.5111{\mathcal{e}}^{i2\Pi \left({\rm O}.{\rm O}991\right)}\right\}\end{array}\right)$$	$$\left(\begin{array}{c}\left\{\begin{array}{c}{\rm O}.0461{\mathcal{e}}^{i2\Pi \left({\rm O}.0991\right)},{\rm O}.0131{\mathcal{e}}^{i2\Pi \left({\rm O}.1721\right)},\\ {\rm O}.06641{\mathcal{e}}^{i2\Pi \left({\rm O}.2651\right)}\end{array}\right\},\\ \left\{{\rm O}.3394{\mathcal{e}}^{i2\Pi \left({\rm O}.5530\right)}\right\},\\ \left\{{\rm O}.4462{\mathcal{e}}^{i2\Pi \left({\rm O}.3394\right)},{\rm O}.7658{\mathcal{e}}^{i2\Pi \left({\rm O}.3394\right)}\right\}\end{array}\right)$$
${\widehat{{\varvec{\eta}}}}_{{\varvec{p}}}^{{\varvec{A}}-4}$	$$\left(\begin{array}{c}\left\{\begin{array}{c}{\rm O}.1279{\mathcal{e}}^{i2\Pi \left({\rm O}.5530\right)},{\rm O}.6598{\mathcal{e}}^{i2\Pi \left({\rm O}.3394\right)}\\ ,{\rm O}.6598{\mathcal{e}}^{i2\Pi \left({\rm O}.8684\right)}\end{array}\right\},\\ \left\{{\rm O}.5111{\mathcal{e}}^{i2\Pi \left({\rm O}.{\rm O}991\right)}\right\},\\ \left\{{\rm O}.6641{\mathcal{e}}^{i2\Pi \left({\rm O}.3781\right)},{\rm O}.8371{\mathcal{e}}^{i2\Pi \left({\rm O}.0991\right)}\right\}\end{array}\right)$$	$$\left(\begin{array}{c}\left\{\begin{array}{c}{\rm O}.0131{\mathcal{e}}^{i2\Pi \left({\rm O}.2651\right)},{\rm O}.3781{\mathcal{e}}^{i2\Pi \left({\rm O}.0991\right)},\\ {\rm O}.3781{\mathcal{e}}^{i2\Pi \left({\rm O}.6641\right)}\end{array}\right\},\\ \left\{{\rm O}.7658{\mathcal{e}}^{i2\Pi \left({\rm O}.3394\right)}\right\},\\ \left\{{\rm O}.8684{\mathcal{e}}^{i2\Pi \left({\rm O}.6598\right)},{\rm O}.9592{\mathcal{e}}^{i2\Pi \left({\rm O}.3394\right)}\right\}\end{array}\right)$$
${\widehat{{\varvec{\eta}}}}_{{\varvec{p}}}^{{\varvec{A}}-5}$	$$\left(\begin{array}{c}\left\{\begin{array}{c}{\rm O}.5530{\mathcal{e}}^{i2\Pi \left({\rm O}.6598\right)},{\rm O}.3394{\mathcal{e}}^{i2\Pi \left({\rm O}.4462\right)},\\ {\rm O}.2331{\mathcal{e}}^{i2\Pi \left({\rm O}.9592\right)}\end{array}\right\},\\ \left\{{\rm O}.2651{\mathcal{e}}^{i2\Pi ({\rm O}.1721)}\right\},\\ \left\{{\rm O}.8371{\mathcal{e}}^{i2\Pi \left({\rm O}.0991\right)},{\rm O}.2651{\mathcal{e}}^{i2\Pi \left({\rm O}.1721\right)}\right\}\end{array}\right)$$	$$\left(\begin{array}{c}\left\{\begin{array}{c}{\rm O}.2651{\mathcal{e}}^{i2\Pi \left({\rm O}.3781\right)},{\rm O}.0991{\mathcal{e}}^{i2\Pi \left({\rm O}.1721\right)}\\ ,{\rm O}.0461{\mathcal{e}}^{i2\Pi \left({\rm O}.8371\right)}\end{array}\right\},\\ \left\{{\rm O}.5530{\mathcal{e}}^{i2\Pi \left({\rm O}.4462\right)}\right\},\\ \left\{{\rm O}.9592{\mathcal{e}}^{i2\Pi \left({\rm O}.3394\right)},{\rm O}.5530{\mathcal{e}}^{i2\Pi \left({\rm O}.4462\right)}\right\}\end{array}\right)$$

Step 3: Evaluate the Score values of the above-aggregated information, see Table 3.

**Table 3:** CTSHF score information.

	CTSHFAAPAWA operator	CTSHFAAPAWG operator
${\widehat{{\varvec{\eta}}}}_{{\varvec{p}}}^{{\varvec{A}}-1}$	0.48089	− 0.34961
${\widehat{{\varvec{\eta}}}}_{{\varvec{p}}}^{{\varvec{A}}-2}$	0.55964	− 0.1567
${\widehat{{\varvec{\eta}}}}_{{\varvec{p}}}^{{\varvec{A}}-3}$	0.36595	− 0.38015
${\widehat{{\varvec{\eta}}}}_{{\varvec{p}}}^{{\varvec{A}}-4}$	0.27124	− 0.52319
${\widehat{{\varvec{\eta}}}}_{{\varvec{p}}}^{{\varvec{A}}-5}$	0.38314	− 0.39479

Step 4: Derive the ranking values according to the score function to examine the best one, see Table 4.

**Table 4:** CTSHF ranking values.

Methods	Ranking values
CTSHFAAWPA operator	${\widehat{\mathbb{H}}}_{p}^{A-2}>{\widehat{\mathbb{H}}}_{p}^{A-1}>{\widehat{\mathbb{H}}}_{p}^{A-5}>{\widehat{\mathbb{H}}}_{p}^{A-3}>{\widehat{\mathbb{H}}}_{p}^{A-4}$
CTSHFAAWPG operator	${\widehat{\mathbb{H}}}_{p}^{A-2}>{\widehat{\mathbb{H}}}_{p}^{A-1}{>\widehat{\mathbb{H}}}_{p}^{A-3}>{\widehat{\mathbb{H}}}_{p}^{A-5}>{\widehat{\mathbb{H}}}_{p}^{A-4}$

The most preferable and the most dominant optimal is ${\widehat{{\varvec{\eta}}}}_{{\varvec{p}}}^{{\varvec{A}}-2}$ according to the theory of CTSHFAAWPA operator and CTSHFAAWPG operator. Additionally, we evaluate the comparison between proposed operators with some existing operators to show the supremacy and validity of the proposed operators.

## Comparative analysis

In this section, we compare the proposed operators with our considered prevailing operators based on FSs and their extensions, because without comparison every paper has no worth. For comparing our ranking results, we are required to collect some existing operators and then try to evaluate our data with the help of existing operators for comparing it with our ranking results to show the validity of the proposed information, therefore, we select the following operators, such as: AAOs for HFSs^26^, AAOs for IFSs^27^, AAOs for PFSs^28,29^, AAOs for CPFSs^30^, AAOs for IFSs^31^, geometric AAOs for IFSs^32^, AAOs for PyFSs^33^, AAOs for QROFSs^34^, AAOs for TSFSs^35^, and AAOs for CTSFSs^36^. Using the data in Table 1, the comparative analysis is listed in Table 5.

**Table 5:** Comparative analysis of the proposed and existing techniques.

Method	Score value	Ranking values
Senapati et al.^26^	$$********$$	$$********$$
Ali et al.^27^	$$********$$	$$********$$
Senapati et al.^28^	$$********$$	$$********$$
Naeem et al.^29^	$$********$$	$$********$$
Ali et al.^30^	$$********$$	$$********$$
Senapati et al.^31^	$$********$$	$$********$$
Senapati et al.^32^	$$********$$	$$********$$
Hussain et al.^33^	$$********$$	$$********$$
Farid et al.^34^	$$********$$	$$********$$
Hussain et al.^35^	$$********$$	$$********$$
Ali and Naeem^36^	$$********$$	$$********$$
CTSHFAAWPA	0.4808,0.5596,0.3659,0.2712,0.3831	${\widehat{\mathbb{H}}}_{p}^{A-2}>{\widehat{\mathbb{H}}}_{p}^{A-1}>{\widehat{\mathbb{H}}}_{p}^{A-5}>{\widehat{\mathbb{H}}}_{p}^{A-3}>{\widehat{\mathbb{H}}}_{p}^{A-4}$
CTSHFAAWPG	− 0.349,− 0.156,− 0.38,− 0.523,− 0.394	${\widehat{\mathbb{H}}}_{p}^{A-2}>{\widehat{\mathbb{H}}}_{p}^{A-1}{>\widehat{\mathbb{H}}}_{p}^{A-3}>{\widehat{\mathbb{H}}}_{p}^{A-5}>{\widehat{\mathbb{H}}}_{p}^{A-4}$

The most preferable and the most dominant optimal is ${\widehat{{\varvec{\eta}}}}_{{\varvec{p}}}^{{\varvec{A}}-2}$ according to the theory of CTSHFAAWPA operator and CTSHFAAWPG operator. After the investigation in Table 5, we observed that the existing operators have no worth or capability to evaluate the data in Table 1 because these all operators are the special cases of the proposed operators.

## Conclusion

The major theme of this manuscript is listed below:

1. We evaluated the novel theory of the CTSHF set and its operational laws.
2. We investigated Aczel–Alsina operational laws for CTSHF information.
3. We derived the CTSHFAAWPA operator, CTSHFAAOWPA operator, CTSHFAAWPG operator, and CTSHFAAOWPG operator. Some properties are also investigated for the above operators.
4. We evaluated the problems of 3D seismic attributes analysis to mine planning under the consideration of the proposed operators, for this, we illustrated the problem of MADM technique for the exposed operators.
5. We demonstrated some examples for making the comparison between prevailing and proposed information to improve the worth of the derived operators.

### Limitations of the proposed theory

CTSHF set theory has a lot of advantages which are discussed in the introduction section, but it is also clear that, if someone provides the truth, abstinence, and falsity grades in the shape of rough sets like lower approximation and upper approximation spaces, then the CTSHF information has been failed. For this, we aim to propose the idea of complex T-spherical hesitant fuzzy rough set theory, which is a superior and more natural technique than existing ones.

### Future direction

In the future, we will evaluate some operators, such as Dombi operators, Hamacher operators, Einstein operators, and Frank operators based on CTSHF information and try to expose their application in artificial intelligence^41,42^, machine learning^43,44^, game theory^45^, neural networks^46,47^, and decision-making problems^48–51^ to evaluate the supremacy of the proposed operators.

## Data Availability

All data generated or analyzed during this study are included in this published article. As there are no supplementary files, with the manuscript.
